# Causal Association Between Sleep Disorders and Chronic Obstructive Pulmonary Disease: A Bidirectional Mendelian Randomization Analysis for Clinical Practice

**DOI:** 10.7759/cureus.95717

**Published:** 2025-10-29

**Authors:** Wenqiang Lu, Jianming Hu, Lihui Ding, Tao Feng, Le Li, Wentao Lei

**Affiliations:** 1 Respiratory Medicine, The First Hospital of Lanzhou University, Lanzhou, CHN

**Keywords:** colocalization analysis, copd, enrichment analysis, genome-wide association study (gwas), mendelian randomization, sleep disorders

## Abstract

Background: Sleep disorders have been associated with chronic obstructive pulmonary sisease (COPD) in observational studies, but causal inferences have not been confirmed. This bidirectional two-sample Mendelian randomization (MR) study, combined with colocalization and enrichment analyses, aims to investigate the potential causal relationship between sleep-associated phenotypes and COPD.

Methods: We conducted a two-sample bidirectional MR analysis using 10 gene variants associated with sleep phenotypes to explore the causal relationship between sleep disorders and COPD. Five methods for MR analysis were utilized. Additionally, sensitivity analyses were performed to evaluate the robustness of our findings. Enrichment and colocalization analyses were carried out to uncover the genetic mechanisms linking sleep phenotypes and COPD.

Results: The inverse variance weighted (IVW) method demonstrated that insomnia and daytime napping significantly increased COPD risk after multiple testing correction (insomnia: OR = 2.288, 95% CI: 1.537-3.407; p = 4.565e-05; daytime napping: OR = 1.577, 95% CI: 1.088-2.286; p = 0.016). A suggestive association was observed for short sleep duration (OR = 3.662, 95% CI: 1.277-10.499; p = 0.016). Notably, colocalization analysis confirmed a shared genetic locus between daytime napping and COPD (rs1561321), strengthening the causal evidence for this specific association. Conversely, COPD was suggestively associated with an increased risk of daytime napping. Enrichment analysis implicated pathways related to synaptic function for daytime napping and insomnia and calcium signaling for short sleep duration.

Conclusions: Our integrated genetic analysis provided suggestive evidence supporting potential causal roles of specific sleep disorders in COPD risk. Notably, the association between daytime napping and COPD was further strengthened by colocalization analysis, suggesting shared genetic mechanisms.

## Introduction

Chronic obstructive pulmonary disease (COPD) is a long-term respiratory disorder marked by ongoing airflow limitation. According to the Global Burden of Disease (GBD) study, COPD ranks as the third leading cause of death worldwide, affecting over 380 million individuals and significantly diminishing their quality of life and functionality status [1]. In addition to respiratory symptoms, COPD is often associated with systemic comorbidities, particularly sleep disorders prevalent [2]. Epidemiological studies indicate that approximately 40%-70% of COPD patients suffer from sleep disturbances, including insomnia, daytime sleepiness, or sleep apnea [3]. Poor sleep quality is significantly linked to an increased risk of acute exacerbations and mortality. Despite these associations, the causal relationship between sleep disorders and COPD remains unclear. Traditional observational studies are often confounded by factors such as smoking and age, and reverse causality - where nocturnal hypoxemia exacerbates sleep disturbances - complicates the interpretation results [4].

In such cases, Mendelian randomization (MR) has emerged as a powerful tool for causal inference, utilizing genetic variants as instrumental variables (IVs) [5]. Since genetic variants are randomly assigned at conception, MR analysis is less influenced by environmental factors confounders [6]. Additionally, since the occurrence and progression of diseases do not change fixed alleles, MR reduces bias from reverse causality [6]. Therefore, MR provides a robust approach to assessing the causal relationship between sleep traits and COPD, bypassing environmental confounding and reverse causality bias. To clarify whether sleep disorders are associated with COPD and to evaluate the directionality of such associations, we performed a two-sample bidirectional MR study using the most up-to-date genome-wide association studies (GWASs), combined with enrichment and colocalization analyses to systematically evaluate the causal associations between 10 sleep phenotypes and COPD as these sleep-related phenotypes can be targets for direct pharmacological treatment and lifestyle alterations, elucidating the impacts of sleep disorders on the progression and management of COPD [7].

Aims and objectives

This study aimed to systematically investigate the bidirectional causal relationships between sleep disorders and COPD using a comprehensive genetic approach. Our primary objective was to assess the causal effects of 10 clinically relevant and modifiable sleep phenotypes (chronotype, daytime napping, daytime sleepiness, insomnia, long and short sleep duration, sleep duration, snoring, morning person, and single-item chronotype) on COPD risk. These phenotypes were selected for their high prevalence [8,9], potential for direct intervention via pharmacological or lifestyle means [10], and availability of large-scale GWAS data [11]. Our secondary objectives were to (1) evaluate the reverse causal effect of COPD on these sleep traits; (2) perform colocalization analysis to identify shared genetic loci; and (3) conduct functional enrichment analysis to explore the underlying biological pathways linking significant sleep phenotypes to COPD.

## Materials and methods

Materials and methods

Study Design

**Figure 1:**
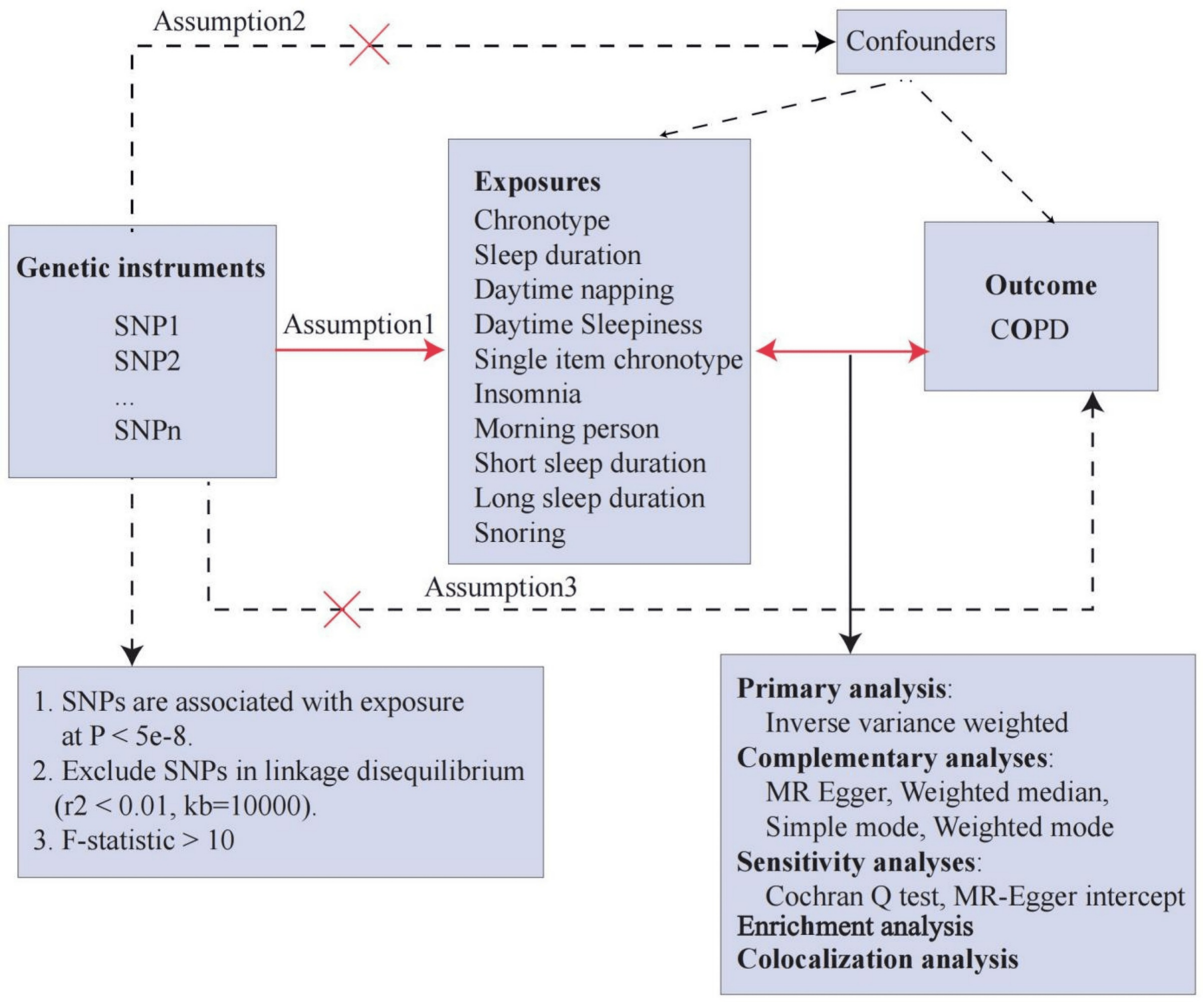
Study design of bidirectional Mendelian randomization analysis MR: Mendelian Randomization; MR Egger: MR Egger Regression; P-value: Probability; r²: Linkage Disequilibrium Correlation Coefficient); kb: Kilobase; F-statistic :Statistical test for model significance; Cochran Q test: Test for heterogeneity; SNP: single-nucleotide polymorphism

Figure 1 summarizes the study's design. The causal association between 10 sleep phenotypes and COPD was assessed through a bidirectional two-sample MR study using data from GWAS. MR analysis employs genetic variants as IVs to estimate the causal relationship between exposure and disease development. The genetic variants follow three assumptions: (1) reliably and robustly related to the exposure, (2) independent of risk factor-outcome confounders, and (3) influence the outcome only via the exposure. In addition to the MR analysis, we conducted a colocalization and functional enrichment analysis to clarify further the genetic mechanisms underlying the association between sleep phenotypes and COPD. All GWAS summary statistics used in the present study have been publicly available, and ethical approval was already obtained in the original studies [12].

Data Sources

GWAS data for chronotype and morning person were obtained from the GWAS Catalog. At the same time, the remaining sleep traits (daytime napping, daytime sleepiness, insomnia, long and short sleep duration, sleep duration, and snoring) were sourced from the UK Biobank [13]. The UK Biobank is a large-scale cohort study, enrolling over 500,000 participants aged 40-69 between 2006 and 2010. GWAS data for COPD were also retrieved from the GWAS Catalog [11,14-16]. Detailed information regarding the 10 sleep traits and COPD included in this study is presented in Table 1.

**Table 1:** Overview of the datasets used in the study GWAS: Genome-Wide Association Study; CV: Continuous Variable; DV: Binary Variable; PMID: PubMed Identifier; SNP: Single-Nucleotide Polymorphism; UK Biobank: United Kingdom Biobank; ICD: International Classification of Diseases; DOI: Digital Object Identifier

Phenotypes	Consortium	Category	Year of publication	Codes*,references or ICD codes	Sample size	Cases(n)	Controls(n)	DOI/URL
exposure								
Chronotype	GWAS Catalog	CV	2019	PMID: 30696823	449,734	-	-	https://ftp.ebi.ac.uk/pub/databases/gwas/summary_statistics/GCST007001-GCST008000/GCST007576/chronotype_raw_BOLT.output_HRC.only_plus.metrics_maf0.001_hwep1em12_info0.3.txt.gz
Daytime napping	UK Biobank	DV	2021	PMID: 33568662	452,633	196,895	255,738	https://personal.broadinstitute.org/ryank/Saxena_fullUKBB_Daytimenapping_summary_stats.zip
Daytime sleepiness	UK Biobank	DV	2019	PMID: 31409809	452,071	104,786	347,285	https://personal.broadinstitute.org/ryank/Saxena.fullUKBB.DaytimeSleepiness.sumstats.zip
Insomnia	UK Biobank	DV	2019	PMID: 30804566	453,379	108,357	345,022	https://personal.broadinstitute.org/ryank/Saxena_fullUKBB_Insomnia_summary_stats.zip
Long sleep duration	UK Biobank	DV	2019	PMID: 30846698	339,926	34,184	305,742	https://personal.broadinstitute.org/ryank/longsumstats.txt.zip
Short sleep duration	UK Biobank	DV	2019	PMID: 30846698	411,934	106,192	305,742	https://personal.broadinstitute.org/ryank/shortsumstats.txt.zip
Sleep duration	UKBiobank	CV	2019	PMID: 30846698	446,118	-	-	https://ftp.ebi.ac.uk/pub/databases/gwas/summary_statistics/GCST007001-GCST008000/GCST007561/Dashti_30846698_sleepdurationsumstats.txt.zip
Snoring	UK Biobank	DV	2018	-	361,194	-	-	https://broad-ukb-sumstats-us-east-1.s3.amazonaws.com/round2/additive-tsvs/1210.gwas.imputed_v3.both_sexes.tsv.bgz
Morning person	GWAS Catalog	DV	2019	PMID: 30696823	403,195	252,287	150,908	https://ftp.ebi.ac.uk/pub/databases/gwas/summary_statistics/GCST007001-GCST008000/GCST007565/morning_person_BOLT.output_HRC.only_plus.metrics_maf0.001_hwep1em12_info0.3_logORs.txt.gz
Single item chronotype	UKBiobank	CV	2020	PMID: 32579418	8433	-	-	https://personal.broadinstitute.org/ryank/summary_stat_gwas_singleitemchronotype.zip
outcome								
COPD	GWAS Catalog	-	2021	PMID: 34594059	468475	13530	454945	http://ftp.ebi.ac.uk/pub/databases/gwas/summary_statistics/GCST90018001-GCST90019000/GCST90018807

Genetic Instrument Selection

We selected IVs in a multi-step process designed to satisfy the three core MR assumptions. To ensure the independence of the IVs (assumption 2) and prevent bias from linkage disequilibrium, we performed clumping with a stringent linkage disequilibrium threshold of r² < 0.001 and a clumping window of 10 Mb. To satisfy the relevance assumption (assumption 1), we initially selected SNPs at a genome-wide significance threshold (p < 5×10⁻⁸), and evaluated instrument strength using the F-statistic, retaining only SNPs with F > 10 [17]. When insufficient SNPs were available, the threshold was relaxed to p < 5×10⁻⁶ [17]. Finally, to uphold the exclusion restriction criterion (assumption 3), we harmonized effect alleles and removed any SNPs directly associated with the COPD outcome (P < 5×10⁻⁸) [18].

Statistical analysis

Employing various types of MR approaches, including inverse variance weighted (IVW), MR Egger regression (MR-Egger), weighted mode, weighted median, and simple mode, we aimed to ascertain MR estimates of sleep disorders for COPD, following standardized effect allele analysis across GWAS for 10 sleep disorder phenotypes and COPD [19]. Utilizing IVW as our primary method for the meta-summarization of multi-loci effects, we proceeded under the idealized condition that each SNP was valid and possessed complete independence [19]. Following the extraction of estimates associating variants with sleep disorders or COPD, we aligned the direction of the forecast according to the effect alleles [19]. In scenarios with more than one SNP, we employed the IVW method, choosing a fixed-effect model for Cochran's Q test p values exceeding 0.05 or a random-effect meta-analysis for p values less than 0.05. Furthermore, various MR methods, such as MR-Egger and weighted median, were employed to evaluate the robustness of the study outcomes. The weighted median, calculated as the median of the distribution of individual SNP effect values ranked by weight, allows for less than 50% of SNPs to be invalid [19]. At the same time, MR-Egger accommodates potential pleiotropy in all SNPs, assuming that pleiotropic effects on the outcome are independent of the impact of exposure factors. Namely, MR-Egger facilitated the assessment of heterogeneity and pleiotropy across the entire set of SNPs, even when horizontal heterogeneity is present [19]. When no less than half of the SNPs were valid, weighted median and weighted model were used to estimate causal effects. The simple mode was conducted to demonstrate whether the observed genotype differences between micronutrients and sleep disorders were statistically significant.

Moreover, we utilized specialised sensitivity analyses to identify and adjust for any underlying heterogeneity and pleiotropy, meeting the essential assumptions of our MR study methodology [19]. To ascertain the heterogeneity of each IV, we employed the heterogeneity test, commonly referred to as Cochran’s Q test. To validate that no directional pleiotropy affects the linkage of micronutrients to sleep disorders, the p value of Cochran’s Q test should be more than 0.05. The choice of the IVW model was contingent on the presence of heterogeneity: the fixed effects model for nonsignificant p values (> 0.05) and the multiplicative random-effects model for significant p values (< 0.05). Moreover, MR-Egger was used to estimate potential biases caused by horizontal pleiotropy and invalid IVs. When the intercept of the regression lacked statistical significance (P < 0.05), the slope in MR-Egger regression could act as an estimate of the outcome caused by exposure effects in the causal relationship. To enhance the interpretability of our results, we conducted a leave-one-out analysis to identify the influence of individual SNPs associated with exposure, then reiterated the IVW analysis in a sequential process, each time omitting a single SNP. The significance of each SNP's impact on the IVW causal estimates was evaluated by conducting a leave-one-out analysis [19]. The study outcomes were quantified as OR (odds ratio), complete with a 95% confidence interval, and all p values were two-sided with a 5% level set for statistical significance [19].

The study outcomes were quantified as odds ratio (OR), complete with a 95% confidence interval, and all p values were two-sided with a 5% level set for statistical significance. Statistical analyses were performed using R (version 4.1.1; R Development Core Team, Vienna, Austria) and the TwoSampleMR package. P < 0.05 was chosen as the level of statistical significance.

To account for multiple testing across the 10 sleep phenotypes, we performed False Discovery Rate (FDR) correction using the Benjamini-Hochberg procedure. Associations with an FDR-corrected p value (q-value) < 0.05 were considered statistically significant, while those with a nominal p value < 0.05 but a q-value ≥ 0.05 were considered suggestive [19].

Colocalization Analysis

We performed colocalization analysis to determine whether the significant sleep phenotypes and COPD shared common causal genetic variants at specific genomic loci. The analysis was conducted using the coloc R package (version 5.2.3). For each sleep phenotype that showed significant causal effects on COPD in the primary MR analysis, we examined genomic regions extending 100 kb upstream and downstream of the lead variant identified in the harmonized MR results [20].

The analysis was conducted using a Bayesian framework that calculates five posterior probabilities: PPH0 (no causal variant for either trait), PPH1 (causal variant for the sleep phenotype only), PPH2 (causal variant for COPD only), PPH3 (two distinct causal variants in the region), and PPH4 (one shared causal variant). We used the following prior probabilities in the coloc.abf function: p1 = 1×10⁻⁴ (prior probability for sleep phenotype having a causal variant), p2 = 1×10⁻⁴ (prior probability for COPD having a causal variant), and p12 = 1×10⁻⁵ (prior probability for both traits sharing a causal variant) [20].

For each SNP region analyzed, we first extracted all variants within the 200 kb window from both the sleep phenotype and COPD GWAS summary statistics. The datasets were harmonized to ensure allele alignment using the harmonise_data function from the TwoSampleMR package (version 0.6.8). We incorporated study-specific parameters, including sample size and case proportions for both traits to properly account for statistical power [20].

A PPH4 value > 75% was considered strong evidence for colocalization, indicating a shared causal genetic variant between the sleep phenotype and COPD. To visualize the genetic associations in colocalized regions, we generated locus comparison plots using the locuscomparer R package, which displays the association p-values for both traits across the genomic region of interest [20].

All analyses were conducted using R version 4.4.1 with the following additional packages: dplyr (version 1.1.4) for data manipulation, data.table (version 1.17.8) for efficient data processing, and ggplot2 (version 3.5.1) for visualization.

Enrichment Analysis

Functional enrichment analysis was conducted to identify biological pathways implicated by the genes associated with the significant sleep phenotypes. The genes for this analysis were mapped from the lead SNPs of each sleep phenotype. Specifically, SNP annotation was performed using the SNPnexus tool, and the resulting gene symbols were converted to standardized ENTREZIDs using the bitr function from the clusterProfiler R package with the org.Hs.eg.db database. Enrichment analyses for Gene Ontology (GO) terms and Kyoto Encyclopedia of Genes and Genomes (KEGG) pathways were performed using the clusterProfiler package [21]. The Benjamini-Hochberg method was applied for p value adjustment in GO analysis, with a minimum gene set size of 1. The background gene set for GO analysis comprised all human genes in the org.Hs.eg.db database, while for KEGG analysis, the background consisted of all human genes in the KEGG database. A significance threshold of p < 0.05 was set, and the top 10 most significant terms from each category (Biological Process, Cellular Component, Molecular Function for GO; all pathways for KEGG) were visualized using the ggplot2 package [21].

## Results

Results of IV selection

In this study, we meticulously selected genetic IVs for ten sleep phenotypes, including chronotype, daytime napping, daytime sleepiness, insomnia, long sleep duration, short sleep duration, expected sleep duration, snoring, Morning person, and single-item chronotype. All data were derived from high-quality GWAS databases that underwent standardized quality control procedures. Screening results revealed that the number of single-nucleotide polymorphisms (SNPs) for the IVs ranged from 4 (for the single circadian rhythm variable) to 196 (for the circadian rhythm type). Specifically, there were 196 SNPs for chronotype, 107 for daytime napping, 38 for daytime sleepiness, 41 for insomnia, 11 for long sleep duration, 25 for short sleep duration, 74 for sleep duration, 31 for snoring, 148 for morning person, and four for the single-item chronotype variable. All IVs met the selection criteria, providing a robust basis for causal inference. The F-statistics for all genetic instruments exceeded 10 (Appendix, Table 3).

Effect of sleep disorders on COPD

**Figure 2:**
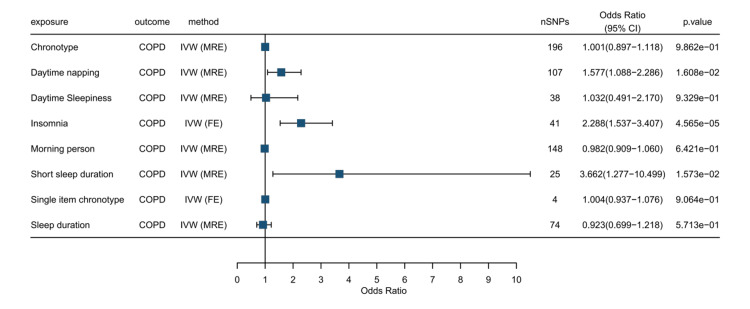
Forest plot showing the effect sizes (OR) and 95% confidence intervals (CI) for the causal effects of 10 sleep phenotypes on COPD IVW: Inverse Variance Weighting; MRE: Mendelian Randomization Egger; FE: Fixed Effect; nSNPs: Number of Single-Nucleotide Polymorphisms; Odds Ratio: A measure of association between an exposure and an outcome; CI: Confidence Interval; p-value: Probability Value; COPD: Chronic Obstructive Pulmonary Disease

**Figure 3:**
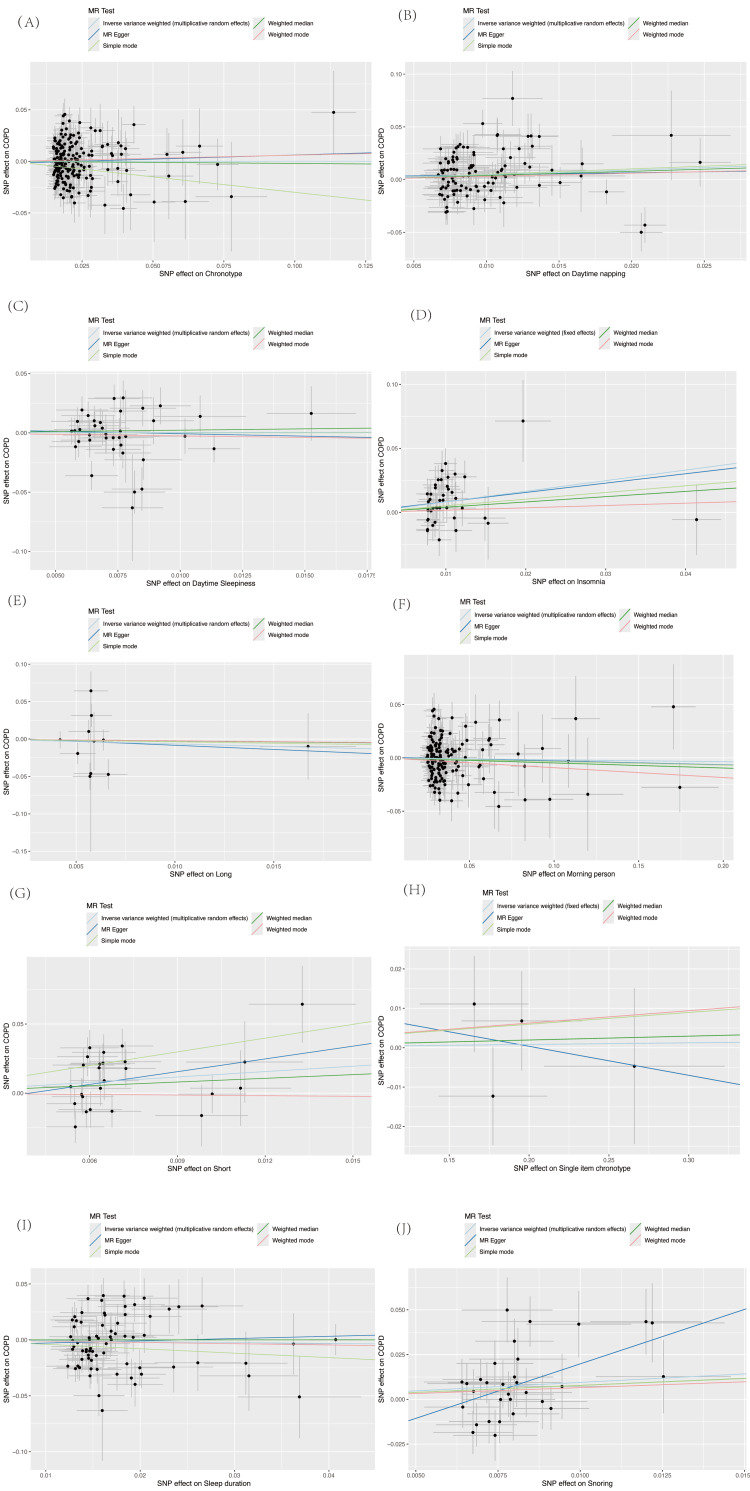
Scatter plot of the causal effects of 10 sleep phenotypes on COPD IVW: Inverse Variance Weighting; SNP: Single-Nucleotide Polymorphism; IVW Test: Inverse Variance Weighting Test; FE: Fixed Effect; MRE: Mendelian Randomization Egger; COPD: Chronic Obstructive Pulmonary Disease The figures show the Mendelian randomization (MR) results for the causal effects of sleep-related traits (via SNP effects) on COPD risk. (A) Scatter plot of MR analysis for the causal effect of chronotype on COPD. (B) Scatter plot of MR analysis for the causal effect of daytime napping on COPD. (C) Scatter plot of MR analysis for the causal effect of daytime sleepiness on COPD. (D) Scatter plot of MR analysis for the causal effect of insomnia on COPD. (E) Scatter plot of MR analysis for the causal effect of long sleep duration on COPD. (F) Scatter plot of MR analysis for the causal effect of morning person on COPD. (G) Scatter plot of MR analysis for the causal effect of short sleep duration on COPD. (H) Scatter plot of MR analysis for the causal effect of single-item chronotype on COPD. (I) Scatter plot of MR analysis for the causal effect of sleep duration on COPD. (J) Scatter plot of MR analysis for the causal effect of snoring on COPD

This study systematically assessed the causal relationships between 10 sleep phenotypes and COPD. The results indicated that insomnia and daytime napping were significantly associated with an increased risk of COPD after FDR correction for multiple testing (insomnia: OR = 2.288, 95% CI: 1.537-3.407, p = 4.57e-05, FDR q = 0.0003; daytime napping: OR = 1.577, 95% CI: 1.088-2.286, p = 0.016, FDR q = 0.033). Short sleep duration showed a suggestive association with COPD that did not survive multiple testing correction (OR = 3.662, 95% CI: 1.277-10.499, p = 0.016, FDR q = 0.032). Figures 2-3 (and Appendix, Table 4) show the five methods and results of our MR analysis.

Results of reverse MR analysis

**Figure 4:**
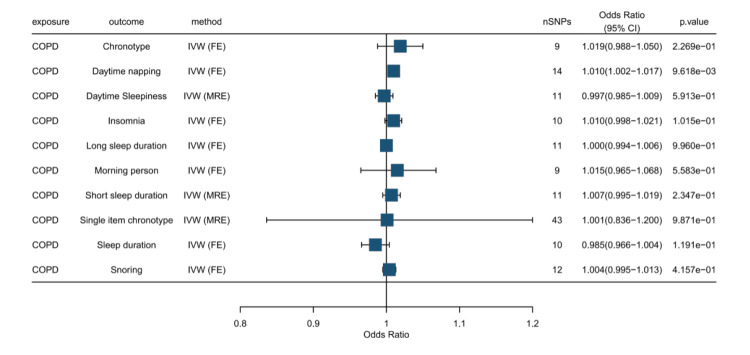
Forest plot illustrating the causal effect of COPD on sleep disorders COPD: Chronic Obstructive Pulmonary Disease; IVW: Inverse Variance Weighting; SNP: Single-Nucleotide Polymorphism; MRE: Mendelian Randomization Egger; MR: Mendelian Randomization; MR Test: Mendelian Randomization Test; IVW Test: Inverse Variance Weighting Test; FE: Fixed Effect

In this study's reverse MR analysis, COPD was the exposure variable, and 10 sleep disorder phenotypes were the outcomes. The results showed a nominally significant positive causal relationship between COPD and daytime napping (OR = 1.010, 95% CI: 1.002-1.017; p = 0.0096). However, this association did not survive correction for multiple testing (FDR q = 0.069) and should therefore be interpreted as suggestive. No significant or suggestive associations were observed between COPD and the other sleep disorder phenotypes after FDR correction (all p > 0.05 and q > 0.05). Figure 4 (and Appendix, Table 5) shows the five methods and results of our MR analysis.

Sensitivity analysis

To assess potential violations of the MR assumptions, we conducted several sensitivity analyses on both forward and reverse MR analyses to verify the robustness of the results. For the forward MR sensitivity analysis (Table 2), Cochran’s Q test revealed significant heterogeneity (p < 0.05) in the effects of chronotype, daytime napping, daytime sleepiness, sleep duration, snoring, and morning person on COPD. No significant heterogeneity (p > 0.05) was observed for the remaining phenotypes. Notably, for the significant forward MR results, the random-effects IVW model was appropriately applied for insomnia and short sleep duration due to heterogeneity, while the association for daytime napping was robust across both fixed-effects and random-effects models. In the reverse MR analysis (Appendix, Table 6), considerable heterogeneity (p < 0.05) was observed for the effects of COPD on daytime napping, daytime sleepiness, short sleep duration, and sleep duration. The presence of heterogeneity in the reverse analysis of daytime napping warrants caution in interpreting this suggestive association. MR-Egger regression analysis indicated significant horizontal pleiotropy for snoring in the forward direction (p < 0.05), while no considerable pleiotropy was found for the other sleep disorder phenotypes (p > 0.05), including the significant and suggestive associations of primary interest. To further exclude the potential impact of heterogeneity and pleiotropy on the results, we performed funnel plot analysis and leave-one-out analysis. The results are shown in Figure 7 (Appendix). For the primary significant findings (insomnia and daytime napping on COPD), the leave-one-out analysis confirmed that no single SNP was driving the causal estimate, and the funnel plots showed general symmetry. Regardless of the method used, the direction of the causal effects for these key associations remained consistent, supporting the robustness of the observed relationships.

**Table 2:** Summary of sensitivity analysis of the causal effect of 10 sleep phenotypes on COPD This table presents the results of a sensitivity analysis for Mendelian randomization (MR) examining the causal effect of 10 sleep phenotypes on COPD. The analysis assesses heterogeneity and horizontal pleiotropy for each sleep phenotype using two methods: IVW and MR Egger. The table reports heterogeneity statistics (Q, Q_df, P, I²) and the intercept, standard error (Se), and p-value for horizontal pleiotropy to assess the potential for confounding bias. COPD: Chronic Obstructive Pulmonary Disease; IVW: Inverse Variance Weighting; MRE: Mendelian Randomization Egger

Exposure	Outcome	Heterogeneity					Horizontal pleiotropy		
		Method	Q	Q_df	P	I2	Intercept	Se	P
Chronotype	COPD	IVW	286.4001	195	2.20000E-05	31.9134272	-0.001743155	0.0039	0.654708
		MR Egger	286.1042	194	1.90000E-05	32.1925347			
Daytime napping	COPD	IVW	201.8569	106	0.00000E+00	47.4875617	0.002592329	0.0063	0.682391
		MR Egger	201.5338	105	0.00000E+00	47.8995463			
Daytime Sleepiness	COPD	IVW	57.59121	37	0.016631254	35.7540859	0.003342121	0.0133	0.803334
		MR Egger	57.4907	36	0.012901123	37.3811744			
Insomnia	COPD	IVW	52.95902	40	0.082354462	24.4699026	0.001011549	0.0082	0.902442
		MR Egger	52.93836	39	0.067422612	26.3294119			
Long sleep duration	COPD	IVW	25.46209	10	0.004535139	60.725928	0.002791035	0.0334	0.935264
		MR Egger	25.44237	9	0.00251889	64.6259372			
Short sleep duration	COPD	IVW	42.13169	24	0.012473436	43.0357586	-0.012148497	0.0176	0.496554
		MR Egger	41.27512	23	0.011012826	44.2763522			
Sleep duration	COPD	IVW	155.6987	73	0.00000E+00	53.1145838	-0.004877415	0.0092	0.596576
		MR Egger	155.0898	72	0.00000E+00	53.5752835			
Snoring	COPD	IVW	46.89156	30	0.025496772	36.0226062	-0.041047404	0.0181	0.030993
		MR Egger	39.83023	29	0.086751197	27.1909769			
Morning person	COPD	IVW	228.5426	147	1.9000E-05	35.6794007	0.000679228	0.0041	0.867197
		MR Egger	228.4987	146	1.50000E-05	36.1046776			
Single-item chronotype	COPD	IVW	2.145523	3	0.542758157	-39.826037	0.015084399	0.0432	0.760228
		MR Egger	2.022176	2	0.363822944	1.09663521			

Colocalization analysis

**Figure 5:**
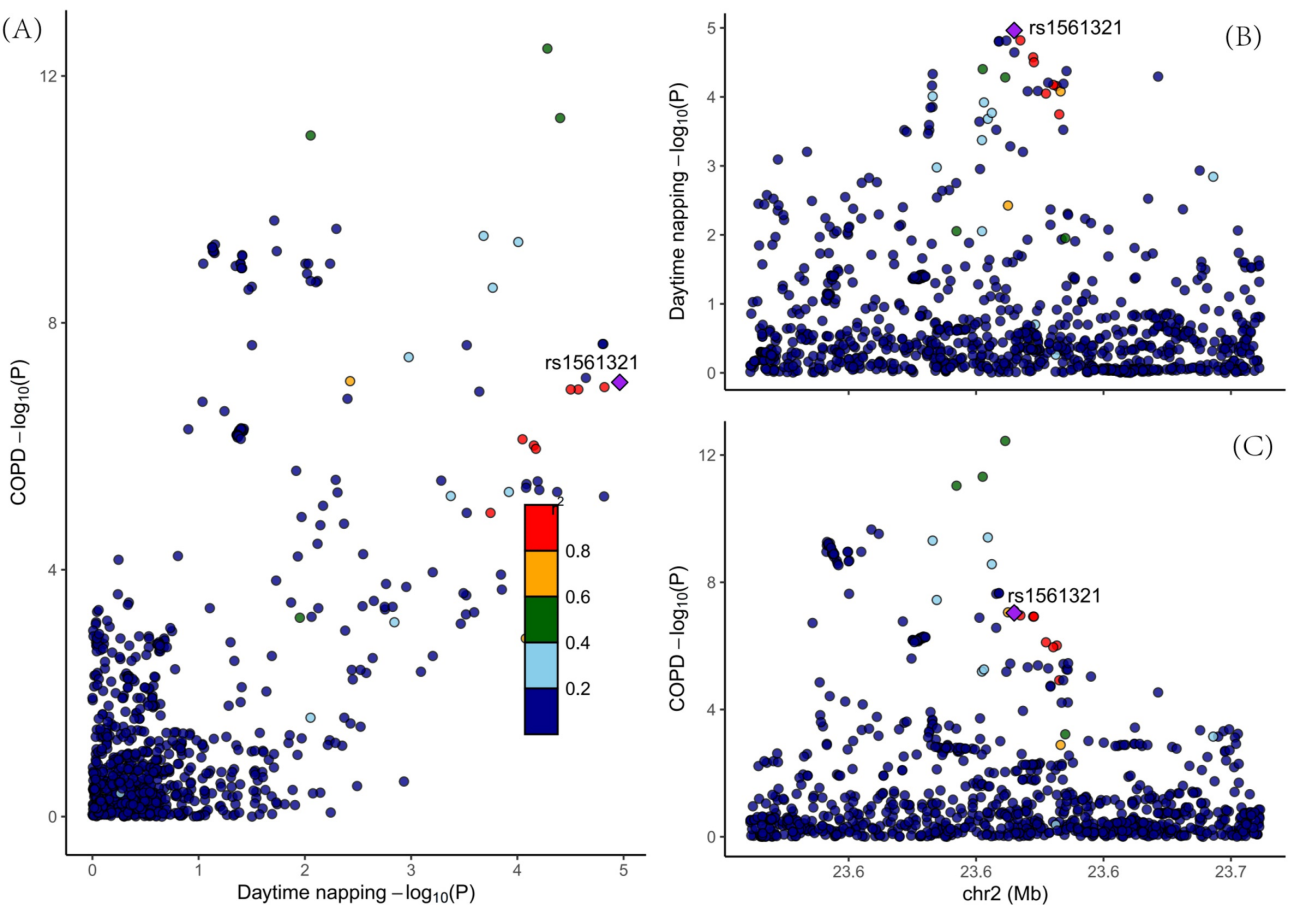
Colocalization analysis of daytime napping and COPD at the rs1561321 locus on chromosome 2 (A): "Genetic Association of Daytime Napping at the rs1561321 Locus on Chromosome 2". (B): "Colocalization Probability for Daytime Napping at the rs1561321 Locus". (C): "Colocalization Probability for COPD at the rs1561321 Locus". P (p-value); rs1561321 (a specific identifier for a single-nucleotide polymorphism) chr2: Chromosome 2; Mb: Megabase, a unit of length for measuring DNA sequences; COPD: Chronic Obstructive Pulmonary Disease

Crucially, colocalization analysis (Figure 5) identified a shared genetic signal only for daytime napping and COPD (rs1561321, PPH4 = 0.99). The lack of colocalization for insomnia and short sleep duration suggests that the MR associations for these traits may be driven by distinct, nearby causal variants or more complex pleiotropic mechanisms, rather than a single shared causal pathway. This finding tempers the causal interpretation for these specific sleep phenotypes (Appendix, Table 7).

Enrichment analysis

**Figure 6:**
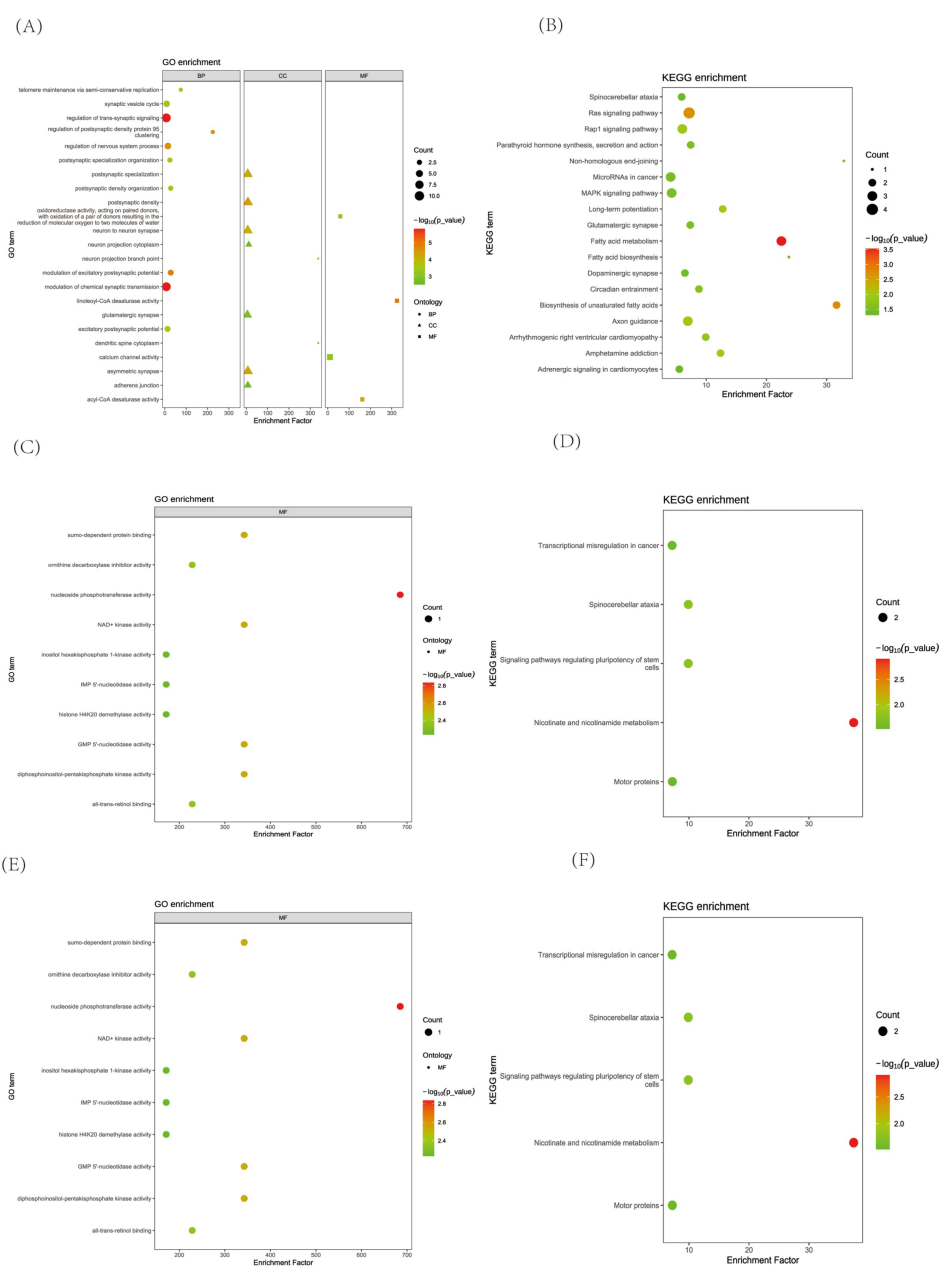
Functional enrichment analysis of sleep disorder phenotype significance results and COPD (A) GO enrichment analysis of daytime napping-related genes. (B) KEGG enrichment analysis of daytime napping-related genes. (C) GO enrichment analysis of insomnia-related genes. (D) KEGG enrichment analysis of insomnia-related genes. (E) GO enrichment analysis of short sleep duration-related genes. (F) KEGG enrichment analysis of short sleep duration-related genes KEGG: Kyoto Encyclopedia of Genes and Genomes; MF: Molecular Function; CC: Cellular Component; BP: Biological Process; FDR: False Discovery Rate; COPD: Chronic Obstructive Pulmonary Disease

**Figure 7:**
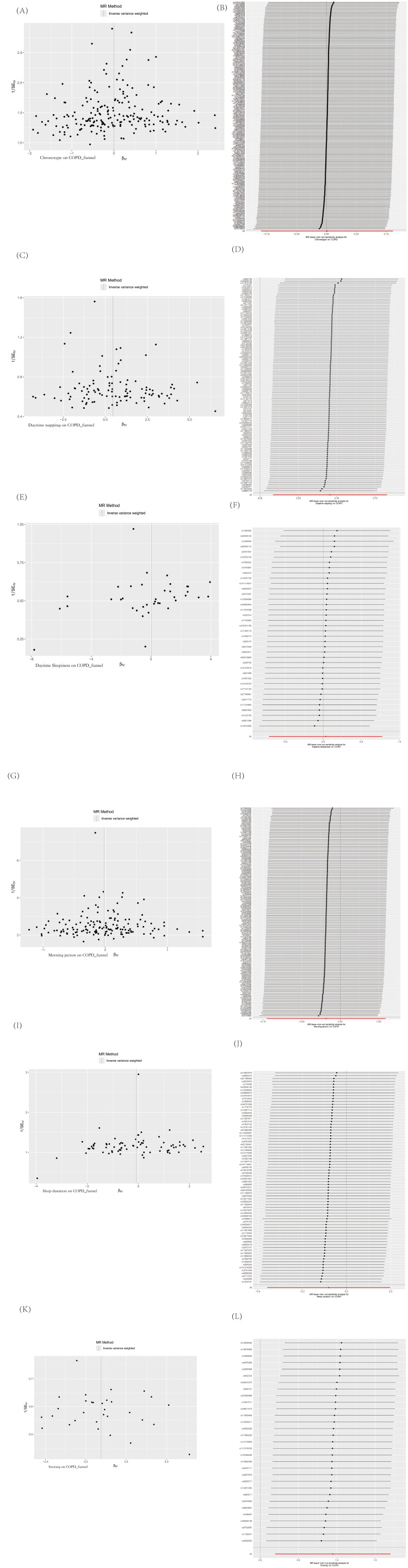
Sensitivity analyses Shows funnel plots and leave-one-out analyses for assessing the robustness of causal effects between sleep phenotypes and COPD, addressing heterogeneity and pleiotropy.

Enrichment analysis was performed to identify molecular pathways potentially involved in the pathogenesis of COPD associated with insomnia, daytime napping, and short sleep duration (Figure 6). GO analysis identified 22 biological processes (BP), seven cellular components (CC), and nine molecular functions (MF) with enrichment observed in pathways related to synaptic transmission and protein aggregation. Kyoto Encyclopedia of Genes and Genomes (KEGG) analysis identified nine enriched neural signal transduction and cellular signaling pathways. Daytime napping showed enrichment in the "synaptic vesicle cycle" and "synapse-associated protein 95 aggregations" pathways according to GO analysis (Figure 6A), with KEGG analysis (Figure 6B) revealing enrichment in the "glutamatergic synapse" and "MAPK signaling pathway." Insomnia was enriched in the "synaptic density" and "postsynaptic specialization" pathways based on GO analysis (Figure 6C), while KEGG analysis (Figure 6D) identified enrichment in the "long-term potentiation" and "glutamatergic synapse" pathways. Finally, short sleep duration was associated with enrichment in the "calcium channel activity" and "glutamatergic synapse" pathways, as indicated by GO analysis (Figure 6E) and KEGG analysis (Figure 6F).

## Discussion

Our study employed an integrated genetic approach to investigate the complex interplay between sleep disorders and COPD. The principal findings suggest a potential causal role of insomnia and daytime napping in the development of COPD, with the association for daytime napping being further reinforced by colocalization analysis. We also observed a suggestive link for short sleep duration and evidence indicating that COPD may increase the risk of daytime napping.

The divergent results from our colocalization analysis provide crucial nuance to the causal interpretation. For daytime napping, the strong colocalization signal (PPH4 > 0.99) at the rs1561321 locus suggests that the MR association is driven by a shared genetic mechanism, greatly strengthening the evidence for a direct causal relationship. In contrast, the lack of colocalization for insomnia and short sleep duration, despite significant MR results, suggests an alternative genetic architecture. For these phenotypes, the causal estimates may be influenced by horizontal pleiotropy, where the genetic variants affect COPD through biological pathways separate from their role in sleep regulation. This key distinction underscores that, while all three sleep phenotypes are genetically associated with COPD, the underlying nature of these relationships may differ fundamentally.

The results of this study are consistent with existing literature. Li et al. [22] reported a significant association between insomnia and COPD, suggesting that insomnia may increase the risk of acute exacerbations in COPD patients by affecting sleep quality and triggering inflammatory responses. Leng et al. [23] found a strong correlation between daytime napping and COPD, proposing that daytime napping could be an early indicator of respiratory diseases such as COPD. A cross-sectional study [24] also indicated that short sleep duration is an independent risk factor for COPD. Recent MR studies [25] have suggested that obstructive sleep apnea (OSA) may protect against COPD. These non-significant results indicate that future studies should expand sample sizes and employ more precise measurement methods to explore potential associations between these phenotypes and COPD.

The reverse MR results suggest that COPD may increase the risk of daytime napping. This finding is consistent with existing literature. Mohamed et al. [26] found that daytime sleepiness and frequent napping are common among COPD patients, with napping duration proportional to the disease severity. Additionally, Clímaco [27] noted that patients with chronic hypoxia are more likely to require daytime naps to cope with excessive fatigue. An observational study [28] found that COPD patients often exhibit circadian rhythm disturbances, exacerbating daytime sleepiness. Hynninen et al. [29] emphasized the high prevalence of abnormal sleep duration and insomnia in COPD patients, noting that about 30% of COPD patients experience severe insomnia symptoms, with sleep quality closely related to the degree of pulmonary function decline. McNicholas et al. [3] observed that snoring and OSA are common among COPD patients and worsen dyspnea, affecting sleep quality. These studies collectively reveal the significant impact of COPD on various sleep phenotypes and suggest potential therapeutic interventions to alleviate sleep disorders in COPD patients.

The functional enrichment analysis offers insights into potential neurobiological mechanisms linking these sleep phenotypes to COPD. Daytime napping was enriched in the synaptic vesicle cycle and synapse-associated protein 95 aggregation pathways; insomnia was linked to synaptic density and postsynaptic specialization; and short sleep duration was associated with calcium channel activity and glutamatergic synapse pathways. These findings converge on dysregulation of synaptic signaling, suggesting that the genetic overlap between sleep disorders and COPD may involve shared vulnerabilities in neural communication and excitability, which could be influenced by the systemic inflammation and chronic hypoxia characteristic of COPD [30].

Strengths and limitations

Our study has several strengths, including the use of a bidirectional MR design to minimize confounding and reverse causality, the utilization of large-scale GWAS data to enhance statistical power, and the integration of colocalization and enrichment analyses to probe shared genetic mechanisms and underlying biology. However, several limitations must be considered when interpreting our findings. First, the strength of genetic evidence varies across the sleep phenotypes. While insomnia and daytime napping remained significant after rigorous multiple testing correction, the association with short sleep duration was suggestive and did not meet the FDR-corrected significance threshold. Moreover, and crucially, colocalization analysis provided strong evidence for a shared genetic variant only for daytime napping and COPD. The lack of colocalization for insomnia and short sleep duration indicates that the observed MR associations for these traits might be driven by horizontal pleiotropy or distinct, closely linked causal variants, thereby tempering definitive causal claims for them. Second, although we employed robust methods for IV selection and sensitivity analyses, residual bias from weak instruments or undetected pleiotropy cannot be entirely ruled out in MR studies. Third, the reliance on self-reported or registry-based definitions for both sleep phenotypes and COPD could introduce misclassification bias. Fourth, as the constituent GWAS were primarily based on individuals of European ancestry, the generalizability of our findings to other populations requires further investigation. Finally, as noted by reviewers, our genetic study design could not directly incorporate or adjust for key environmental risk factors of COPD, such as smoking history or air pollution exposure. Future research integrating individual-level data is warranted to explore the interplay between genetic predisposition to sleep disorders and these critical environmental factors in the pathogenesis of COPD.

## Conclusions

In conclusion, our integrated genetic investigation provides robust but nuanced evidence regarding the sleep-COPD relationship. We identify strong genetic support for a causal link between daytime napping and COPD, bolstered by colocalization analysis, and suggestive evidence for a role of insomnia. The shared neurobiological pathways uncovered offer new mechanistic insights. These findings underscore the clinical relevance of sleep health, particularly the management of daytime napping, in COPD care. They also highlight the distinct genetic underpinnings of different sleep phenotypes, calling for further research to clarify their specific roles in COPD pathogenesis.

## Appendices

**Table 3:** MR_SNP_Selection This table summarizes data for single nucleotide polymorphism (SNP) selection in Mendelian randomization (MR) analysis, involving 10 sleep types. It includes SNP identifiers, chromosomal positions, allele info, effect sizes, sample sizes, associated sleep exposures, statistical metrics (e.g., p-values, beta values), and selection-related markers to identify SNPs meeting MR requirements.

SNP	chr.exposure	pos.exposure	effect_allele.exposure	other_allele.exposure	eaf.exposure	beta.exposure	se.exposure	pval.exposure	exposure	mr_keep.exposure	pval_origin.exposure	id.exposure	samplesize.exposure
rs61773390	1	7884525	G	T	0.803561	-0.0340792	0.00337553	1.20E-23	Chronotype	TRUE	reported	NtPGiJ	449734
rs113387037	1	7978235	G	A	0.9386	0.0330468	0.00569033	1.10E-08	Chronotype	TRUE	reported	NtPGiJ	449734
rs476430	1	14532613	T	A	0.426783	-0.0155354	0.00271457	1.00E-08	Chronotype	TRUE	reported	NtPGiJ	449734
rs17448682	1	15966713	C	T	0.768048	-0.0221528	0.00318247	2.20E-12	Chronotype	TRUE	reported	NtPGiJ	449734
rs3934192	1	20026472	A	G	0.32827	0.0181089	0.00285642	7.30E-10	Chronotype	TRUE	reported	NtPGiJ	449734
rs3816454	1	21191416	T	G	0.374768	0.020082	0.00278156	4.90E-13	Chronotype	TRUE	reported	NtPGiJ	449734
rs12140153	1	62579891	G	T	0.904737	0.0340109	0.00468803	1.60E-13	Chronotype	TRUE	reported	NtPGiJ	449734
rs2744915	1	71471781	G	A	0.61016	0.014967	0.00276129	4.50E-08	Chronotype	TRUE	reported	NtPGiJ	449734
rs7547493	1	77775233	A	G	0.821984	-0.0376299	0.00350319	3.90E-27	Chronotype	TRUE	reported	NtPGiJ	449734
rs6658041	1	79387676	G	A	0.40008	-0.0152482	0.00274124	1.70E-08	Chronotype	TRUE	reported	NtPGiJ	449734
rs11588913	1	79963816	G	A	0.601681	0.0154525	0.00273856	1.50E-08	Chronotype	TRUE	reported	NtPGiJ	449734
rs112386754	1	91083792	G	A	0.886438	0.0231349	0.00425307	2.50E-08	Chronotype	TRUE	reported	NtPGiJ	449734
rs72720396	1	91191582	A	G	0.769656	-0.0271261	0.00318521	8.40E-18	Chronotype	TRUE	reported	NtPGiJ	449734
rs4949980	1	96621739	A	G	0.932393	-0.0321893	0.00539402	2.70E-09	Chronotype	TRUE	reported	NtPGiJ	449734
rs11165756	1	97473587	C	A	0.809522	0.0215941	0.00341918	1.50E-10	Chronotype	TRUE	reported	NtPGiJ	449734
rs17575798	1	110086451	G	A	0.807172	0.0225553	0.00339377	4.20E-11	Chronotype	TRUE	reported	NtPGiJ	449734
rs11587758	1	150327106	G	A	0.604485	-0.0241103	0.0027375	5.50E-19	Chronotype	TRUE	reported	NtPGiJ	449734
rs607518	1	150954671	G	A	0.781351	-0.0183847	0.00324623	9.90E-09	Chronotype	TRUE	reported	NtPGiJ	449734
rs75650221	1	174421994	C	T	0.961622	-0.0421207	0.00699783	1.40E-09	Chronotype	TRUE	reported	NtPGiJ	449734
rs975025	1	179338327	C	T	0.922916	0.0321062	0.00502525	1.30E-10	Chronotype	TRUE	reported	NtPGiJ	449734
rs509476	1	182573227	T	C	0.0301569	0.1137	0.0078575	5.70E-47	Chronotype	TRUE	reported	NtPGiJ	449734
rs13011556	2	4651923	C	G	0.761404	-0.0227424	0.00316075	1.10E-12	Chronotype	TRUE	reported	NtPGiJ	449734
rs2712056	2	23959131	C	T	0.815336	-0.022175	0.00345992	5.10E-11	Chronotype	TRUE	reported	NtPGiJ	449734
rs848552	2	36700580	C	G	0.47228	-0.0153219	0.00268579	7.00E-09	Chronotype	TRUE	reported	NtPGiJ	449734
rs75120545	2	44271496	C	T	0.969821	-0.0613917	0.00828587	1.10E-13	Chronotype	TRUE	reported	NtPGiJ	449734
rs786406	2	44735953	A	G	0.297873	-0.0235784	0.00293536	1.90E-16	Chronotype	TRUE	reported	NtPGiJ	449734
rs12713014	2	48986474	A	G	0.942045	0.0353634	0.00579576	1.10E-09	Chronotype	TRUE	reported	NtPGiJ	449734
rs10495976	2	49750698	A	T	0.609779	-0.0178512	0.00276698	5.10E-11	Chronotype	TRUE	reported	NtPGiJ	449734
rs111626088	2	50465217	C	A	0.924378	-0.0313664	0.00509283	6.60E-10	Chronotype	TRUE	reported	NtPGiJ	449734
rs10193431	2	53725767	T	C	0.525892	0.0163321	0.00268854	1.10E-09	Chronotype	TRUE	reported	NtPGiJ	449734
rs10175975	2	59429807	C	T	0.819526	-0.0228441	0.00351376	1.80E-10	Chronotype	TRUE	reported	NtPGiJ	449734
rs359250	2	60480973	G	T	0.363664	-0.0168642	0.0027885	9.20E-10	Chronotype	TRUE	reported	NtPGiJ	449734
rs812925	2	61680993	C	G	0.648756	-0.0205641	0.00280786	6.90E-13	Chronotype	TRUE	reported	NtPGiJ	449734
rs113851554	2	66750564	G	T	0.942753	0.0357353	0.00598996	1.00E-09	Chronotype	TRUE	reported	NtPGiJ	449734
rs4852864	2	69818033	T	C	0.592891	-0.0149792	0.00272573	4.90E-08	Chronotype	TRUE	reported	NtPGiJ	449734
rs2706762	2	70488470	C	T	0.850258	0.0219218	0.00375432	4.00E-09	Chronotype	TRUE	reported	NtPGiJ	449734
rs2422413	2	76343577	T	C	0.622358	-0.0162894	0.0027689	3.60E-09	Chronotype	TRUE	reported	NtPGiJ	449734
rs10520176	2	77217310	T	C	0.50187	0.0231498	0.00268932	1.40E-17	Chronotype	TRUE	reported	NtPGiJ	449734
rs4316965	2	77887421	T	C	0.373748	-0.0159238	0.00278976	1.20E-08	Chronotype	TRUE	reported	NtPGiJ	449734
rs28380327	2	144232491	A	T	0.629124	0.0205097	0.00277698	2.90E-13	Chronotype	TRUE	reported	NtPGiJ	449734
rs1947198	2	149390435	C	T	0.877845	-0.0241964	0.00409707	1.80E-09	Chronotype	TRUE	reported	NtPGiJ	449734
rs197273	2	161894663	A	G	0.470208	0.0158713	0.00269376	1.30E-08	Chronotype	TRUE	reported	NtPGiJ	449734
rs11677484	2	191578172	G	T	0.744305	-0.0185564	0.00309256	1.70E-09	Chronotype	TRUE	reported	NtPGiJ	449734
rs1064213	2	198950240	G	A	0.522312	-0.019141	0.00268602	1.30E-12	Chronotype	TRUE	reported	NtPGiJ	449734
rs184033703	2	206956138	G	A	0.942021	-0.0377937	0.00576165	8.10E-11	Chronotype	TRUE	reported	NtPGiJ	449734
rs960783	2	239149570	C	T	0.955376	0.060508	0.00654133	2.90E-20	Chronotype	TRUE	reported	NtPGiJ	449734
rs116298301	2	239177686	C	T	0.971966	-0.0664317	0.00832197	4.20E-16	Chronotype	TRUE	reported	NtPGiJ	449734
rs57435966	2	239311369	C	T	0.912868	0.0549152	0.0047647	2.60E-30	Chronotype	TRUE	reported	NtPGiJ	449734
rs62182135	2	240267305	C	A	0.66833	0.0179439	0.00284795	3.70E-10	Chronotype	TRUE	reported	NtPGiJ	449734
rs149611468	3	8817423	T	C	0.988036	0.0776374	0.0125983	8.50E-10	Chronotype	TRUE	reported	NtPGiJ	449734
rs6442446	3	14394752	A	G	0.290534	0.0174738	0.00297308	3.10E-09	Chronotype	TRUE	reported	NtPGiJ	449734
rs6577620	3	18177308	C	T	0.547454	0.0170777	0.00271211	2.30E-10	Chronotype	TRUE	reported	NtPGiJ	449734
rs7428484	3	23330101	A	G	0.673986	0.0166064	0.00286097	3.60E-09	Chronotype	TRUE	reported	NtPGiJ	449734
rs13059636	3	24935790	A	G	0.527951	-0.0182698	0.00270236	1.60E-11	Chronotype	TRUE	reported	NtPGiJ	449734
rs114848860	3	36859494	A	T	0.975537	-0.0555837	0.00872612	3.60E-10	Chronotype	TRUE	reported	NtPGiJ	449734
rs113441737	3	46764220	G	A	0.921588	-0.0279515	0.00510592	1.70E-08	Chronotype	TRUE	reported	NtPGiJ	449734
rs11712056	3	49914397	T	C	0.556296	0.0200896	0.00270384	1.60E-13	Chronotype	TRUE	reported	NtPGiJ	449734
rs13316611	3	63564385	G	T	0.743413	-0.0178305	0.0030789	1.70E-08	Chronotype	TRUE	reported	NtPGiJ	449734
rs11924337	3	77321570	G	C	0.727554	-0.0190701	0.00301826	2.20E-10	Chronotype	TRUE	reported	NtPGiJ	449734
rs957501	3	85791657	T	A	0.336064	0.0154211	0.00284661	3.00E-08	Chronotype	TRUE	reported	NtPGiJ	449734
rs1800828	3	113891549	C	G	0.746407	0.0176235	0.00308462	1.70E-08	Chronotype	TRUE	reported	NtPGiJ	449734
rs72966564	3	123149816	C	T	0.750914	0.0174371	0.00312692	2.50E-08	Chronotype	TRUE	reported	NtPGiJ	449734
rs13065394	3	132971327	G	T	0.711537	0.017504	0.00296404	3.00E-09	Chronotype	TRUE	reported	NtPGiJ	449734
rs1871516	3	138132434	C	T	0.330978	-0.0158288	0.00285183	2.00E-08	Chronotype	TRUE	reported	NtPGiJ	449734
rs2102506	3	156342849	G	A	0.360336	0.0174762	0.00281907	1.20E-09	Chronotype	TRUE	reported	NtPGiJ	449734
rs9831488	3	160755062	A	G	0.649533	-0.0183592	0.00284523	8.40E-11	Chronotype	TRUE	reported	NtPGiJ	449734
rs3850174	3	172364093	T	A	0.742891	0.0194471	0.00309938	6.80E-10	Chronotype	TRUE	reported	NtPGiJ	449734
rs4490386	3	182256996	G	A	0.692499	0.0180125	0.00291001	1.70E-09	Chronotype	TRUE	reported	NtPGiJ	449734
rs2239626	3	185998884	T	C	0.695411	-0.0202758	0.00292696	2.30E-12	Chronotype	TRUE	reported	NtPGiJ	449734
rs7617588	3	186079990	T	C	0.860627	-0.0237263	0.00387134	1.50E-09	Chronotype	TRUE	reported	NtPGiJ	449734
rs231398	4	2832189	G	A	0.837853	0.0207867	0.00367183	8.40E-09	Chronotype	TRUE	reported	NtPGiJ	449734
rs28634184	4	62873419	C	T	0.749137	0.0176363	0.00310689	2.80E-08	Chronotype	TRUE	reported	NtPGiJ	449734
rs7691121	4	83274208	C	G	0.767817	0.0220069	0.00317772	5.60E-12	Chronotype	TRUE	reported	NtPGiJ	449734
rs4339281	4	118315560	A	G	0.873018	0.0223188	0.00404047	3.20E-08	Chronotype	TRUE	reported	NtPGiJ	449734
rs4241964	4	137053959	T	G	0.524911	-0.020735	0.00270449	1.10E-14	Chronotype	TRUE	reported	NtPGiJ	449734
rs10067113	5	26016363	C	T	0.380901	-0.016251	0.00277635	6.30E-09	Chronotype	TRUE	reported	NtPGiJ	449734
rs10058356	5	35220404	C	T	0.301192	0.0169817	0.00293612	6.80E-09	Chronotype	TRUE	reported	NtPGiJ	449734
rs7701529	5	63861475	A	T	0.237864	-0.0188448	0.00317247	3.60E-09	Chronotype	TRUE	reported	NtPGiJ	449734
rs12523282	5	87703496	T	C	0.747357	0.0212671	0.00308978	5.80E-12	Chronotype	TRUE	reported	NtPGiJ	449734
rs56947091	5	88160354	C	T	0.488674	-0.0191881	0.00268737	6.00E-13	Chronotype	TRUE	reported	NtPGiJ	449734
rs1976423	5	104042643	A	C	0.504045	-0.0189056	0.00269094	2.60E-12	Chronotype	TRUE	reported	NtPGiJ	449734
rs286805	5	107460819	A	T	0.476954	0.0143071	0.00269548	4.50E-08	Chronotype	TRUE	reported	NtPGiJ	449734
rs1027742	5	115904410	A	G	0.737765	0.0183119	0.00306482	2.80E-09	Chronotype	TRUE	reported	NtPGiJ	449734
rs4246036	5	152205430	C	T	0.292331	0.0237433	0.00295896	2.40E-15	Chronotype	TRUE	reported	NtPGiJ	449734
rs7735794	5	175339984	G	A	0.775911	-0.0201147	0.00340433	4.70E-09	Chronotype	TRUE	reported	NtPGiJ	449734
rs651001	6	11569402	G	A	0.569703	0.0155881	0.0027154	1.10E-08	Chronotype	TRUE	reported	NtPGiJ	449734
rs9395520	6	13183523	C	T	0.694567	-0.0231272	0.00291912	2.10E-15	Chronotype	TRUE	reported	NtPGiJ	449734
rs521977	6	31836827	T	G	0.322419	0.0159787	0.00286438	2.10E-08	Chronotype	TRUE	reported	NtPGiJ	449734
rs4714475	6	41509901	G	T	0.754058	0.0190271	0.00317856	1.60E-09	Chronotype	TRUE	reported	NtPGiJ	449734
rs3857599	6	50938247	C	A	0.836962	-0.0218522	0.00363894	1.40E-09	Chronotype	TRUE	reported	NtPGiJ	449734
rs1555531	6	54425924	G	A	0.889215	-0.0283742	0.00426902	1.90E-11	Chronotype	TRUE	reported	NtPGiJ	449734
rs2653349	6	55142337	A	G	0.214552	0.0387249	0.00327448	5.20E-32	Chronotype	TRUE	reported	NtPGiJ	449734
rs7743404	6	55215190	A	C	0.458185	-0.016019	0.002696	1.50E-09	Chronotype	TRUE	reported	NtPGiJ	449734
rs9476307	6	57749349	A	G	0.58946	-0.0159195	0.00273629	7.00E-09	Chronotype	TRUE	reported	NtPGiJ	449734
rs2881955	6	72479263	C	T	0.72122	-0.0161216	0.00300229	4.30E-08	Chronotype	TRUE	reported	NtPGiJ	449734
rs60616179	6	110244765	A	G	0.945102	0.0375809	0.00594669	1.10E-10	Chronotype	TRUE	reported	NtPGiJ	449734
rs827749	6	153129663	T	C	0.672364	-0.0236615	0.00286775	1.00E-16	Chronotype	TRUE	reported	NtPGiJ	449734
rs9364767	6	165221821	T	G	0.446852	0.0158538	0.00269933	5.40E-09	Chronotype	TRUE	reported	NtPGiJ	449734
rs9348050	6	166263488	T	C	0.48949	0.016638	0.00269048	7.50E-10	Chronotype	TRUE	reported	NtPGiJ	449734
rs10280205	7	24082063	T	C	0.691748	0.0174497	0.00291239	1.40E-09	Chronotype	TRUE	reported	NtPGiJ	449734
rs17161045	7	32261458	T	C	0.630545	0.0206395	0.0028029	7.20E-14	Chronotype	TRUE	reported	NtPGiJ	449734
rs56049037	7	32947201	G	A	0.712303	0.0207567	0.00297982	7.30E-12	Chronotype	TRUE	reported	NtPGiJ	449734
rs6967481	7	50642701	C	T	0.503437	-0.0206433	0.00269925	6.90E-15	Chronotype	TRUE	reported	NtPGiJ	449734
rs2968511	7	71810805	C	G	0.695292	-0.0174599	0.00291928	4.90E-09	Chronotype	TRUE	reported	NtPGiJ	449734
rs4729303	7	96474878	C	T	0.186849	-0.0246891	0.00344383	1.10E-12	Chronotype	TRUE	reported	NtPGiJ	449734
rs202157	7	101637753	C	T	0.298716	0.0255486	0.0029433	4.10E-18	Chronotype	TRUE	reported	NtPGiJ	449734
rs77655131	7	102086552	C	T	0.874196	0.0273926	0.00408599	1.10E-11	Chronotype	TRUE	reported	NtPGiJ	449734
rs4729854	7	102383663	T	A	0.516562	0.0287186	0.00275036	3.30E-25	Chronotype	TRUE	reported	NtPGiJ	449734
rs2189008	7	114123308	C	T	0.507411	-0.0155929	0.00269632	2.00E-08	Chronotype	TRUE	reported	NtPGiJ	449734
rs1113295	7	115678646	T	G	0.440869	0.0156591	0.00270408	6.80E-09	Chronotype	TRUE	reported	NtPGiJ	449734
rs2971970	7	133643778	T	G	0.218075	0.0188272	0.00325601	6.00E-09	Chronotype	TRUE	reported	NtPGiJ	449734
rs35524253	8	4823608	G	A	0.642782	-0.015862	0.00281259	5.80E-09	Chronotype	TRUE	reported	NtPGiJ	449734
rs11786306	8	8208759	G	C	0.646022	-0.0183309	0.00283083	8.70E-11	Chronotype	TRUE	reported	NtPGiJ	449734
rs1994058	8	32015145	G	A	0.125279	-0.0238971	0.00408646	1.90E-09	Chronotype	TRUE	reported	NtPGiJ	449734
rs12541362	8	33584661	A	T	0.649758	-0.0213568	0.00282446	3.50E-14	Chronotype	TRUE	reported	NtPGiJ	449734
rs2980378	8	35185529	G	A	0.592152	-0.0156339	0.00273366	1.80E-08	Chronotype	TRUE	reported	NtPGiJ	449734
rs769066	8	53145698	T	C	0.816519	-0.0219436	0.00348289	3.20E-10	Chronotype	TRUE	reported	NtPGiJ	449734
rs4321976	8	76614958	T	C	0.77856	0.0232959	0.00324034	3.10E-13	Chronotype	TRUE	reported	NtPGiJ	449734
rs3100052	8	101967139	A	G	0.387351	0.0170795	0.00276058	6.90E-10	Chronotype	TRUE	reported	NtPGiJ	449734
rs3808477	8	116670347	C	T	0.718601	-0.0215613	0.00299116	2.80E-13	Chronotype	TRUE	reported	NtPGiJ	449734
rs10758942	9	8145891	A	G	0.357303	0.0167001	0.00280506	2.30E-09	Chronotype	TRUE	reported	NtPGiJ	449734
rs10976942	9	8310813	C	A	0.91702	-0.0282753	0.00487069	7.00E-09	Chronotype	TRUE	reported	NtPGiJ	449734
rs6477309	9	8450638	C	T	0.334608	-0.0174399	0.00286276	8.00E-10	Chronotype	TRUE	reported	NtPGiJ	449734
rs308521	9	37367094	T	C	0.602627	0.0199718	0.00275427	3.70E-13	Chronotype	TRUE	reported	NtPGiJ	449734
rs62553781	9	76679777	C	T	0.965171	0.0504004	0.00734656	1.20E-11	Chronotype	TRUE	reported	NtPGiJ	449734
rs295268	9	86429305	T	C	0.744325	-0.0165883	0.00307939	4.10E-08	Chronotype	TRUE	reported	NtPGiJ	449734
rs10818834	9	126317324	T	C	0.732704	0.0188673	0.00304915	1.10E-09	Chronotype	TRUE	reported	NtPGiJ	449734
rs4838161	9	127079253	T	G	0.560809	0.0161104	0.00270516	2.20E-09	Chronotype	TRUE	reported	NtPGiJ	449734
rs10988239	9	131943440	C	T	0.48728	0.0186334	0.00273304	1.60E-11	Chronotype	TRUE	reported	NtPGiJ	449734
rs7628	9	139318071	A	G	0.50227	-0.016	0.00268565	1.10E-09	Chronotype	TRUE	reported	NtPGiJ	449734
rs28458909	9	140257189	C	T	0.876368	0.0432803	0.00407749	2.60E-26	Chronotype	TRUE	reported	NtPGiJ	449734
rs4948547	10	60568514	C	A	0.255795	0.0190042	0.00307909	1.20E-09	Chronotype	TRUE	reported	NtPGiJ	449734
rs10823233	10	70351903	T	C	0.452113	-0.0151686	0.00270212	4.30E-08	Chronotype	TRUE	reported	NtPGiJ	449734
rs3808964	10	125426627	G	T	0.365271	-0.0157096	0.00279601	2.70E-08	Chronotype	TRUE	reported	NtPGiJ	449734
rs72632979	11	16615883	A	G	0.82849	0.021581	0.00357606	1.20E-09	Chronotype	TRUE	reported	NtPGiJ	449734
rs12798330	11	19184916	A	G	0.962009	0.0388668	0.00702635	4.40E-08	Chronotype	TRUE	reported	NtPGiJ	449734
rs10742179	11	27650524	A	G	0.260177	0.0182967	0.00306465	1.50E-09	Chronotype	TRUE	reported	NtPGiJ	449734
rs640245	11	30393360	C	T	0.516644	-0.0182658	0.00269872	9.70E-12	Chronotype	TRUE	reported	NtPGiJ	449734
rs11032362	11	33759092	G	A	0.909237	-0.0395538	0.00467579	4.80E-17	Chronotype	TRUE	reported	NtPGiJ	449734
rs34239319	11	43902681	G	T	0.897879	-0.0280114	0.00443834	3.90E-10	Chronotype	TRUE	reported	NtPGiJ	449734
rs3168135	11	58386177	G	A	0.760045	0.0207725	0.0031468	2.90E-11	Chronotype	TRUE	reported	NtPGiJ	449734
rs11227452	11	66059105	G	A	0.828358	-0.0194632	0.00358768	4.60E-08	Chronotype	TRUE	reported	NtPGiJ	449734
rs4237555	11	92725803	C	T	0.473046	-0.0164771	0.00269332	6.00E-10	Chronotype	TRUE	reported	NtPGiJ	449734
rs4936291	11	114009982	A	G	0.610748	-0.0179295	0.00283312	3.80E-10	Chronotype	TRUE	reported	NtPGiJ	449734
rs577924	11	122135107	C	T	0.464897	-0.0155309	0.00270069	1.00E-08	Chronotype	TRUE	reported	NtPGiJ	449734
rs74357745	11	122811822	A	G	0.878807	0.026546	0.00412156	8.70E-11	Chronotype	TRUE	reported	NtPGiJ	449734
rs7943634	11	126734319	C	T	0.690715	0.016455	0.00291003	1.40E-08	Chronotype	TRUE	reported	NtPGiJ	449734
rs10772978	12	17038538	T	A	0.644659	0.0164843	0.00280771	3.50E-09	Chronotype	TRUE	reported	NtPGiJ	449734
rs12811046	12	24066937	A	G	0.553343	0.0169679	0.00270358	6.20E-10	Chronotype	TRUE	reported	NtPGiJ	449734
rs7308565	12	34018179	C	T	0.599391	-0.0203591	0.00276787	2.00E-13	Chronotype	TRUE	reported	NtPGiJ	449734
rs11181153	12	38289817	C	T	0.413964	0.0240619	0.00273011	1.20E-18	Chronotype	TRUE	reported	NtPGiJ	449734
rs7313852	12	38908389	G	A	0.564964	0.029649	0.00271587	2.70E-27	Chronotype	TRUE	reported	NtPGiJ	449734
rs11183201	12	46170982	T	C	0.49221	-0.0179025	0.0026954	4.60E-11	Chronotype	TRUE	reported	NtPGiJ	449734
rs55947153	12	54683639	T	A	0.588479	0.0161167	0.00273752	1.10E-09	Chronotype	TRUE	reported	NtPGiJ	449734
rs77384811	12	63497398	G	C	0.843254	0.0247483	0.00372778	2.80E-11	Chronotype	TRUE	reported	NtPGiJ	449734
rs7959983	12	90452978	T	C	0.595918	-0.0195258	0.00272849	9.50E-13	Chronotype	TRUE	reported	NtPGiJ	449734
rs7304278	12	106989915	A	G	0.274848	-0.020255	0.00302264	2.20E-11	Chronotype	TRUE	reported	NtPGiJ	449734
rs741334	12	112985328	A	G	0.711693	0.0164509	0.0029772	2.40E-08	Chronotype	TRUE	reported	NtPGiJ	449734
rs80097534	12	121029604	G	T	0.901458	0.0281025	0.00455453	9.30E-10	Chronotype	TRUE	reported	NtPGiJ	449734
rs9597250	13	56303709	C	A	0.810212	0.0213068	0.00343801	6.90E-10	Chronotype	TRUE	reported	NtPGiJ	449734
rs9573971	13	77568732	A	G	0.9661	0.0727759	0.00743443	3.60E-22	Chronotype	TRUE	reported	NtPGiJ	449734
rs35687504	13	77688544	C	A	0.809996	0.0201251	0.00346773	7.20E-09	Chronotype	TRUE	reported	NtPGiJ	449734
rs17267683	13	94057870	C	G	0.76125	0.0197414	0.00316651	2.70E-10	Chronotype	TRUE	reported	NtPGiJ	449734
rs9521184	13	109782568	T	C	0.48682	0.0148855	0.00270389	3.00E-08	Chronotype	TRUE	reported	NtPGiJ	449734
rs1952923	14	40982168	G	A	0.260667	0.016877	0.00305963	3.60E-08	Chronotype	TRUE	reported	NtPGiJ	449734
rs698015	14	57269825	C	T	0.352507	-0.0161876	0.0028566	8.80E-09	Chronotype	TRUE	reported	NtPGiJ	449734
rs7148842	14	74255556	C	T	0.611932	0.0153777	0.00278312	1.80E-08	Chronotype	TRUE	reported	NtPGiJ	449734
rs10149448	14	84253271	A	G	0.603599	0.0164465	0.00275282	2.70E-09	Chronotype	TRUE	reported	NtPGiJ	449734
rs7144406	14	98659847	A	G	0.790526	0.0184778	0.00331646	2.20E-08	Chronotype	TRUE	reported	NtPGiJ	449734
rs12432176	14	101021218	C	A	0.620705	-0.0161131	0.00278357	6.50E-09	Chronotype	TRUE	reported	NtPGiJ	449734
rs59986227	15	48009263	C	G	0.74221	-0.0178401	0.00310217	8.30E-09	Chronotype	TRUE	reported	NtPGiJ	449734
rs11641239	16	23124193	C	T	0.711428	-0.0172486	0.00296753	1.10E-08	Chronotype	TRUE	reported	NtPGiJ	449734
rs9932577	16	24512509	C	A	0.495067	0.0166195	0.00271482	6.80E-10	Chronotype	TRUE	reported	NtPGiJ	449734
rs12927162	16	52684916	A	G	0.721593	0.0286498	0.00300067	6.80E-22	Chronotype	TRUE	reported	NtPGiJ	449734
rs1421085	16	53800954	T	C	0.596882	-0.0275621	0.00274075	1.50E-23	Chronotype	TRUE	reported	NtPGiJ	449734
rs2550298	16	56367969	C	T	0.622827	0.0239989	0.0027767	3.60E-18	Chronotype	TRUE	reported	NtPGiJ	449734
rs8063159	16	60627491	C	A	0.610918	-0.0192263	0.00275742	1.50E-12	Chronotype	TRUE	reported	NtPGiJ	449734
rs17604349	16	72210865	G	A	0.820797	0.0257422	0.00351443	1.70E-13	Chronotype	TRUE	reported	NtPGiJ	449734
rs2518022	17	8057367	T	C	0.0836026	0.0403075	0.00485089	4.80E-17	Chronotype	TRUE	reported	NtPGiJ	449734
rs3760185	17	17401736	C	T	0.751745	0.0241586	0.0031496	2.60E-14	Chronotype	TRUE	reported	NtPGiJ	449734
rs226090	17	34034059	C	T	0.168356	0.0208783	0.00359523	4.10E-09	Chronotype	TRUE	reported	NtPGiJ	449734
rs35653190	17	46077967	C	T	0.771675	0.0182444	0.00322514	1.20E-08	Chronotype	TRUE	reported	NtPGiJ	449734
rs28647842	17	50127211	G	C	0.477366	0.0160844	0.00269439	2.00E-09	Chronotype	TRUE	reported	NtPGiJ	449734
rs72829706	17	54173733	A	G	0.960574	0.0407112	0.00693598	5.10E-09	Chronotype	TRUE	reported	NtPGiJ	449734
rs17682747	17	61181112	G	A	0.767686	-0.0184215	0.00319016	7.20E-09	Chronotype	TRUE	reported	NtPGiJ	449734
rs2949923	17	65475228	A	G	0.538377	-0.016206	0.00270197	2.10E-09	Chronotype	TRUE	reported	NtPGiJ	449734
rs2580160	18	1816036	A	G	0.552952	0.0153499	0.00273342	1.70E-08	Chronotype	TRUE	reported	NtPGiJ	449734
rs62082401	18	5186019	C	G	0.80901	-0.0256287	0.00342253	1.60E-14	Chronotype	TRUE	reported	NtPGiJ	449734
rs10460095	18	22624708	G	A	0.427893	-0.0181914	0.0027251	1.40E-11	Chronotype	TRUE	reported	NtPGiJ	449734
rs4239386	18	31664710	T	A	0.665104	0.0218962	0.00285562	1.00E-14	Chronotype	TRUE	reported	NtPGiJ	449734
rs12969848	18	38152835	C	T	0.470141	-0.0218632	0.00270301	1.30E-15	Chronotype	TRUE	reported	NtPGiJ	449734
rs9962650	18	53574043	C	G	0.576681	-0.0173129	0.00272797	1.60E-10	Chronotype	TRUE	reported	NtPGiJ	449734
rs9964420	18	56824041	C	A	0.695492	0.0284801	0.00293643	8.60E-22	Chronotype	TRUE	reported	NtPGiJ	449734
rs56076457	18	60238668	C	T	0.471265	-0.0162984	0.00269462	1.30E-09	Chronotype	TRUE	reported	NtPGiJ	449734
rs10402849	19	2695661	C	T	0.798647	-0.0192609	0.00335946	8.90E-09	Chronotype	TRUE	reported	NtPGiJ	449734
rs36055559	19	5799433	G	A	0.827204	0.0210912	0.00372199	1.50E-08	Chronotype	TRUE	reported	NtPGiJ	449734
rs1053007	19	10754400	A	G	0.367522	-0.0153999	0.00278963	2.40E-08	Chronotype	TRUE	reported	NtPGiJ	449734
rs9636202	19	18449238	G	A	0.733565	0.0183734	0.00305534	7.50E-10	Chronotype	TRUE	reported	NtPGiJ	449734
rs11670534	19	47003906	C	T	0.834382	0.0209755	0.00362802	8.50E-09	Chronotype	TRUE	reported	NtPGiJ	449734
rs12462111	19	49171306	C	T	0.535169	0.0158171	0.00273479	2.20E-08	Chronotype	TRUE	reported	NtPGiJ	449734
rs78095690	20	16239683	T	C	0.564376	-0.0163477	0.00272137	1.40E-09	Chronotype	TRUE	reported	NtPGiJ	449734
rs6131942	20	17348608	A	G	0.419965	-0.0175437	0.00273235	9.40E-11	Chronotype	TRUE	reported	NtPGiJ	449734
rs2072727	20	43538733	T	C	0.435988	0.016352	0.00271514	8.50E-10	Chronotype	TRUE	reported	NtPGiJ	449734
rs139911	22	40704052	C	T	0.423865	0.0233915	0.00273231	2.30E-17	Chronotype	TRUE	reported	NtPGiJ	449734
rs4822107	22	42704834	G	A	0.494278	-0.015528	0.00270621	9.60E-09	Chronotype	TRUE	reported	NtPGiJ	449734
SNP	chr.exposure	pos.exposure	effect_allele.exposure	other_allele.exposure	eaf.exposure	beta.exposure	se.exposure	pval.exposure	samplesize.exposure	exposure	mr_keep.exposure	pval_origin.exposure	id.exposure
rs301817	1	8503379	C	A	0.4106	-0.00734402	0.00123666	1.60E-09	452633	Daytime napping	TRUE	reported	2E2GmH
rs34751289	1	32074069	T	TA	0.373268	0.0072472	0.00125782	5.60E-09	452633	Daytime napping	TRUE	reported	2E2GmH
rs2786547	1	33342304	C	T	0.823104	0.0107673	0.00158775	1.00E-11	452633	Daytime napping	TRUE	reported	2E2GmH
rs11383197	1	34998659	A	AG	0.324624	-0.00719036	0.00129454	2.70E-08	452633	Daytime napping	TRUE	reported	2E2GmH
rs35549521	1	46028185	C	CT	0.569893	0.00737113	0.00127693	1.50E-08	452633	Daytime napping	TRUE	reported	2E2GmH
rs41313250	1	62483623	A	G	0.879238	0.013072	0.00191501	8.50E-12	452633	Daytime napping	TRUE	reported	2E2GmH
rs12140153	1	62579891	G	T	0.904603	0.0247147	0.00211564	2.40E-31	452633	Daytime napping	TRUE	reported	2E2GmH
rs2893323	1	95783037	G	A	0.645942	-0.0069416	0.00126675	3.30E-08	452633	Daytime napping	TRUE	reported	2E2GmH
rs1931175	1	96928273	C	G	0.616172	-0.00776325	0.00124756	4.60E-10	452633	Daytime napping	TRUE	reported	2E2GmH
rs1843815	1	98589715	A	T	0.454402	-0.00727388	0.00121793	3.30E-09	452633	Daytime napping	TRUE	reported	2E2GmH
rs12031519	1	162876200	A	G	0.888258	0.0112838	0.00194275	5.50E-09	452633	Daytime napping	TRUE	reported	2E2GmH
rs35039375	1	171516863	A	G	0.908168	-0.0136431	0.00210973	8.70E-11	452633	Daytime napping	TRUE	reported	2E2GmH
rs200435329	1	174316633	G	T	0.762084	-0.00986281	0.0014379	9.90E-12	452633	Daytime napping	TRUE	reported	2E2GmH
rs2250377	1	201860626	A	G	0.338883	0.0131561	0.00127975	1.40E-24	452633	Daytime napping	TRUE	reported	2E2GmH
rs13033444	2	23611438	A	G	0.717195	-0.00974179	0.00135018	3.60E-13	452633	Daytime napping	TRUE	reported	2E2GmH
rs56180058	2	25337155	C	T	0.839025	0.00942616	0.00165866	1.30E-08	452633	Daytime napping	TRUE	reported	2E2GmH
rs9309116	2	44557919	C	T	0.347169	-0.00737914	0.00127277	3.90E-09	452633	Daytime napping	TRUE	reported	2E2GmH
rs13023587	2	49413860	C	G	0.491428	0.00744772	0.00121195	6.50E-10	452633	Daytime napping	TRUE	reported	2E2GmH
rs350785	2	52934600	T	C	0.115476	0.0126479	0.00190975	1.50E-11	452633	Daytime napping	TRUE	reported	2E2GmH
rs17049683	2	58905715	A	G	0.666681	-0.00795171	0.00129066	4.10E-10	452633	Daytime napping	TRUE	reported	2E2GmH
rs11125776	2	59478517	T	G	0.854478	0.0116502	0.00172495	1.50E-11	452633	Daytime napping	TRUE	reported	2E2GmH
rs9287862	2	151500518	C	T	0.0867737	0.0122526	0.00215162	2.00E-08	452633	Daytime napping	TRUE	reported	2E2GmH
rs62189006	2	162573586	A	G	0.907199	0.0120943	0.0020982	8.30E-09	452633	Daytime napping	TRUE	reported	2E2GmH
rs80163246	2	164574344	T	C	0.882545	-0.0119129	0.00188438	1.40E-10	452633	Daytime napping	TRUE	reported	2E2GmH
rs35144585	2	169092428	T	A	0.870708	0.0103105	0.00182249	1.20E-08	452633	Daytime napping	TRUE	reported	2E2GmH
rs3732085	2	206831860	C	A	0.64248	0.00720767	0.00126622	1.50E-08	452633	Daytime napping	TRUE	reported	2E2GmH
rs7422655	2	208941004	C	T	0.263115	0.00783348	0.00137827	1.10E-08	452633	Daytime napping	TRUE	reported	2E2GmH
rs908442	2	236800163	A	T	0.591346	0.0102781	0.00123558	1.40E-16	452633	Daytime napping	TRUE	reported	2E2GmH
rs77154532	3	19377311	A	G	0.641674	0.00769324	0.0012705	1.40E-09	452633	Daytime napping	TRUE	reported	2E2GmH
rs936944	3	44385814	G	A	0.162179	0.00913528	0.00164374	3.20E-08	452633	Daytime napping	TRUE	reported	2E2GmH
rs76824303	3	62459819	A	C	0.900584	0.0117977	0.00207749	1.70E-08	452633	Daytime napping	TRUE	reported	2E2GmH
rs9883093	3	82774831	T	G	0.366775	-0.007341	0.00125779	4.50E-09	452633	Daytime napping	TRUE	reported	2E2GmH
rs1601440	3	84665393	C	T	0.278622	0.00912653	0.00135575	1.90E-11	452633	Daytime napping	TRUE	reported	2E2GmH
rs2699869	3	133032892	A	C	0.454128	0.00678084	0.00121774	3.10E-08	452633	Daytime napping	TRUE	reported	2E2GmH
rs150538418	3	138064170	G	GT	0.784017	0.00888893	0.00151925	7.20E-09	452633	Daytime napping	TRUE	reported	2E2GmH
rs1001817	3	183995341	C	T	0.507212	0.00775426	0.00121418	1.70E-10	452633	Daytime napping	TRUE	reported	2E2GmH
rs9998136	4	79550425	C	G	0.25275	-0.00881596	0.00140269	4.40E-10	452633	Daytime napping	TRUE	reported	2E2GmH
rs13150944	4	85408000	A	G	0.310614	-0.00860076	0.0013218	6.80E-11	452633	Daytime napping	TRUE	reported	2E2GmH
rs140008192	4	152967845	G	GTA	0.567725	-0.00717917	0.00132316	4.30E-08	452633	Daytime napping	TRUE	reported	2E2GmH
rs4356873	4	164216739	T	C	0.760004	-0.00775689	0.00142205	4.50E-08	452633	Daytime napping	TRUE	reported	2E2GmH
rs76536937	4	170222592	T	TA	0.518858	0.00753249	0.00124291	8.00E-10	452633	Daytime napping	TRUE	reported	2E2GmH
rs12657723	5	62760828	C	T	0.678317	-0.00818319	0.00129849	2.00E-10	452633	Daytime napping	TRUE	reported	2E2GmH
rs6452787	5	87712831	A	G	0.536153	0.00772931	0.0012169	2.00E-10	452633	Daytime napping	TRUE	reported	2E2GmH
rs2943023	5	89597733	C	T	0.580197	0.00710422	0.00123055	7.40E-09	452633	Daytime napping	TRUE	reported	2E2GmH
rs1898673	5	102293380	G	C	0.670857	-0.0101216	0.00129119	6.70E-15	452633	Daytime napping	TRUE	reported	2E2GmH
rs2431108	5	103947968	T	C	0.671277	-0.0129827	0.00129028	7.70E-24	452633	Daytime napping	TRUE	reported	2E2GmH
rs4357022	5	106311039	T	G	0.535844	-0.00682396	0.00121553	1.40E-08	452633	Daytime napping	TRUE	reported	2E2GmH
rs388016	5	106861892	A	G	0.666763	0.00738134	0.00128736	6.10E-09	452633	Daytime napping	TRUE	reported	2E2GmH
rs2099810	5	112030173	A	G	0.50307	0.00769192	0.00121447	2.80E-10	452633	Daytime napping	TRUE	reported	2E2GmH
rs10875606	5	143769614	C	A	0.315767	0.00737707	0.0013077	1.30E-08	452633	Daytime napping	TRUE	reported	2E2GmH
rs10875622	5	146693062	G	A	0.424635	-0.0104621	0.0012291	1.30E-17	452633	Daytime napping	TRUE	reported	2E2GmH
rs73817091	5	159042652	C	T	0.957533	-0.0165127	0.00302224	3.80E-08	452633	Daytime napping	TRUE	reported	2E2GmH
rs11967137	6	28199764	A	G	0.805519	0.00927928	0.00153285	1.70E-09	452633	Daytime napping	TRUE	reported	2E2GmH
rs34262487	6	36224315	C	A	0.927997	0.0145107	0.00235463	1.20E-09	452633	Daytime napping	TRUE	reported	2E2GmH
rs6919087	6	37699156	T	G	0.6876	0.0107806	0.0013114	1.00E-16	452633	Daytime napping	TRUE	reported	2E2GmH
rs4236060	6	38470087	C	T	0.727719	-0.00882528	0.00137545	8.80E-11	452633	Daytime napping	TRUE	reported	2E2GmH
rs927184	6	51829890	T	A	0.33853	-0.00718519	0.00129258	3.50E-08	452633	Daytime napping	TRUE	reported	2E2GmH
rs7743188	6	54773639	C	T	0.903761	0.0128648	0.00206234	2.70E-10	452633	Daytime napping	TRUE	reported	2E2GmH
rs2653349	6	55142337	A	G	0.214777	0.0165942	0.00147827	3.40E-29	452633	Daytime napping	TRUE	reported	2E2GmH
rs113493369	6	57698806	C	CA	0.529714	0.00734219	0.00122966	2.80E-09	452633	Daytime napping	TRUE	reported	2E2GmH
rs1546977	6	84314423	A	G	0.560345	-0.00827718	0.00122321	1.00E-11	452633	Daytime napping	TRUE	reported	2E2GmH
rs9389556	6	100079774	C	G	0.739053	-0.00841517	0.00138285	9.70E-10	452633	Daytime napping	TRUE	reported	2E2GmH
rs785145	6	114650655	T	G	0.568545	-0.0069889	0.00122527	1.00E-08	452633	Daytime napping	TRUE	reported	2E2GmH
rs614987	6	124920871	A	C	0.385795	-0.0110217	0.00124925	7.50E-19	452633	Daytime napping	TRUE	reported	2E2GmH
rs140506252	6	155129280	A	T	0.977522	0.0227261	0.0040886	4.30E-08	452633	Daytime napping	TRUE	reported	2E2GmH
rs9460110	6	170621249	T	C	0.630218	-0.00735568	0.00125751	8.40E-09	452633	Daytime napping	TRUE	reported	2E2GmH
rs35851551	7	31330785	A	G	0.898496	0.011194	0.00202654	3.50E-08	452633	Daytime napping	TRUE	reported	2E2GmH
rs10257273	7	99188589	A	T	0.85069	0.0107366	0.00170413	8.90E-10	452633	Daytime napping	TRUE	reported	2E2GmH
rs113960908	7	106931292	G	GGT	0.772934	-0.00891319	0.00145069	1.00E-09	452633	Daytime napping	TRUE	reported	2E2GmH
rs13263535	8	1175131	G	T	0.531464	0.00687135	0.00121641	1.60E-08	452633	Daytime napping	TRUE	reported	2E2GmH
rs351776	8	28191306	A	C	0.450482	-0.00755367	0.00122072	8.40E-10	452633	Daytime napping	TRUE	reported	2E2GmH
rs2059639	8	67381858	C	T	0.479443	0.0066823	0.0012172	2.40E-08	452633	Daytime napping	TRUE	reported	2E2GmH
rs285815	8	106108303	T	A	0.455535	0.00753009	0.00121983	5.80E-10	452633	Daytime napping	TRUE	reported	2E2GmH
rs7814873	8	142213997	C	T	0.382994	0.00720972	0.00126385	1.50E-08	452633	Daytime napping	TRUE	reported	2E2GmH
rs10811438	9	20962282	G	C	0.60658	0.00721671	0.0012428	3.20E-09	452633	Daytime napping	TRUE	reported	2E2GmH
rs62560863	9	34081331	C	T	0.899684	-0.0113172	0.0020241	2.50E-08	452633	Daytime napping	TRUE	reported	2E2GmH
rs17502738	9	37441650	T	C	0.804111	0.00870459	0.00153004	2.00E-08	452633	Daytime napping	TRUE	reported	2E2GmH
rs1415218	9	73504992	T	C	0.281328	0.00820697	0.00135243	1.20E-09	452633	Daytime napping	TRUE	reported	2E2GmH
rs13284688	9	81727018	T	C	0.793428	-0.0150717	0.00149894	1.70E-23	452633	Daytime napping	TRUE	reported	2E2GmH
rs141957346	9	86367994	A	AT	0.412958	-0.00812867	0.00124433	7.10E-11	452633	Daytime napping	TRUE	reported	2E2GmH
rs971415	9	108846445	A	G	0.877622	0.0110652	0.00185108	1.20E-09	452633	Daytime napping	TRUE	reported	2E2GmH
rs4604518	9	120555845	G	A	0.546474	0.00691205	0.00122091	1.40E-08	452633	Daytime napping	TRUE	reported	2E2GmH
rs12346996	9	131840856	T	C	0.271929	0.00796106	0.00136459	5.00E-09	452633	Daytime napping	TRUE	reported	2E2GmH
rs11258652	10	13868855	C	A	0.763185	0.0104004	0.00142918	3.70E-13	452633	Daytime napping	TRUE	reported	2E2GmH
rs224111	10	64552010	G	A	0.612306	0.00798411	0.00124912	1.60E-10	452633	Daytime napping	TRUE	reported	2E2GmH
rs10840017	11	8335754	A	G	0.767369	0.00894142	0.00148101	2.00E-09	452633	Daytime napping	TRUE	reported	2E2GmH
rs10835420	11	28866669	T	A	0.750826	0.00889802	0.00140206	1.70E-10	452633	Daytime napping	TRUE	reported	2E2GmH
rs174541	11	61565908	T	C	0.639549	-0.00988214	0.00126293	4.40E-15	452633	Daytime napping	TRUE	reported	2E2GmH
rs271057	11	92769748	C	T	0.248427	0.00866783	0.00140414	6.50E-10	452633	Daytime napping	TRUE	reported	2E2GmH
rs11224896	11	101479583	T	C	0.889747	0.0111605	0.00193872	1.10E-08	452633	Daytime napping	TRUE	reported	2E2GmH
rs2417268	12	13493906	A	T	0.559809	-0.00752263	0.00123523	1.20E-09	452633	Daytime napping	TRUE	reported	2E2GmH
rs35011311	12	38616581	G	T	0.734419	0.009082	0.00138249	4.40E-11	452633	Daytime napping	TRUE	reported	2E2GmH
rs60222088	12	108334477	C	A	0.852733	0.0112358	0.00172293	6.40E-11	452633	Daytime napping	TRUE	reported	2E2GmH
rs11615756	12	117942076	C	T	0.595389	-0.0182673	0.00123883	1.40E-49	452633	Daytime napping	TRUE	reported	2E2GmH
rs2769916	13	107820389	G	A	0.311383	-0.00875328	0.00131344	1.70E-11	452633	Daytime napping	TRUE	reported	2E2GmH
rs4983329	14	29218473	A	C	0.555832	0.00767251	0.0012207	3.10E-10	452633	Daytime napping	TRUE	reported	2E2GmH
rs10149986	14	29721044	T	G	0.814802	-0.0109384	0.00156608	4.40E-12	452633	Daytime napping	TRUE	reported	2E2GmH
rs2370926	14	79602542	T	C	0.633526	0.00813213	0.00126359	1.70E-10	452633	Daytime napping	TRUE	reported	2E2GmH
rs11071755	15	63793936	G	A	0.574922	0.00711401	0.00122819	5.20E-09	452633	Daytime napping	TRUE	reported	2E2GmH
rs17158413	15	83235408	G	A	0.762721	-0.00925014	0.00142955	4.40E-11	452633	Daytime napping	TRUE	reported	2E2GmH
rs10152428	15	93510243	G	C	0.270235	-0.00780688	0.00136996	1.10E-08	452633	Daytime napping	TRUE	reported	2E2GmH
rs60920123	16	10134637	G	A	0.566808	0.00763778	0.00122697	4.50E-10	452633	Daytime napping	TRUE	reported	2E2GmH
rs9939355	16	23837048	C	T	0.442329	0.00726404	0.00122438	4.50E-09	452633	Daytime napping	TRUE	reported	2E2GmH
rs3986805	16	28350059	A	G	0.585408	-0.00719435	0.00123765	8.70E-09	452633	Daytime napping	TRUE	reported	2E2GmH
rs11860072	16	56193408	C	T	0.552791	0.00897773	0.00123952	2.80E-13	452633	Daytime napping	TRUE	reported	2E2GmH
rs13330072	16	69402831	A	G	0.713047	-0.00791082	0.00134123	4.80E-09	452633	Daytime napping	TRUE	reported	2E2GmH
rs12451365	17	35595368	T	C	0.795362	-0.010628	0.00150388	1.50E-12	452633	Daytime napping	TRUE	reported	2E2GmH
rs4793173	17	43127715	A	C	0.37067	0.00803948	0.00125702	2.70E-10	452633	Daytime napping	TRUE	reported	2E2GmH
rs385199	17	43686419	A	C	0.772138	0.0209131	0.0014497	7.50E-47	452633	Daytime napping	TRUE	reported	2E2GmH
rs55938136	17	43798360	A	G	0.775462	0.0206586	0.00145316	1.70E-45	452633	Daytime napping	TRUE	reported	2E2GmH
rs2532326	17	44355778	G	T	0.488033	0.00826498	0.00135583	1.30E-09	452633	Daytime napping	TRUE	reported	2E2GmH
rs112520848	17	64308310	G	C	0.614116	-0.00702449	0.00125191	1.80E-08	452633	Daytime napping	TRUE	reported	2E2GmH
rs3935190	17	79084367	G	A	0.463748	-0.00801432	0.00122354	5.40E-11	452633	Daytime napping	TRUE	reported	2E2GmH
rs962247	18	23084118	G	A	0.523199	0.00796355	0.00122493	5.70E-11	452633	Daytime napping	TRUE	reported	2E2GmH
rs8092870	18	25562831	C	T	0.590175	-0.00699531	0.00125126	2.00E-08	452633	Daytime napping	TRUE	reported	2E2GmH
rs34728579	18	31610848	T	C	0.799798	-0.00839996	0.00152087	3.70E-08	452633	Daytime napping	TRUE	reported	2E2GmH
rs2861805	18	36105007	A	G	0.540036	0.0093112	0.00122068	2.00E-14	452633	Daytime napping	TRUE	reported	2E2GmH
rs9965170	18	44788274	G	A	0.576607	0.0136374	0.00122874	7.80E-29	452633	Daytime napping	TRUE	reported	2E2GmH
rs17265513	20	39832628	T	C	0.800599	-0.00913188	0.00151965	2.00E-09	452633	Daytime napping	TRUE	reported	2E2GmH
rs910187	20	45841052	G	A	0.626033	0.00731754	0.00125594	4.90E-09	452633	Daytime napping	TRUE	reported	2E2GmH
rs3810484	20	62194103	A	G	0.556304	0.00684147	0.001222	2.20E-08	452633	Daytime napping	TRUE	reported	2E2GmH
rs2836912	21	40506194	G	A	0.688305	-0.00729991	0.00131115	3.30E-08	452633	Daytime napping	TRUE	reported	2E2GmH
rs1883048	21	47397586	T	C	0.476439	-0.00787143	0.00122313	1.80E-10	452633	Daytime napping	TRUE	reported	2E2GmH
rs2284015	22	37096573	C	G	0.740828	-0.00771167	0.00138662	4.10E-08	452633	Daytime napping	TRUE	reported	2E2GmH
SNP	chr.exposure	pos.exposure	effect_allele.exposure	other_allele.exposure	eaf.exposure	beta.exposure	se.exposure	pval.exposure	exposure	mr_keep.exposure	pval_origin.exposure	id.exposure	samplesize.exposure
rs12140153	1	62579891	G	T	0.904572	0.015256	0.00179131	1.50E-17	Daytime Sleepiness	TRUE	reported	R9ve1g	452071
rs553314	1	90967306	T	C	0.365253	0.00635973	0.00107298	3.70E-09	Daytime Sleepiness	TRUE	reported	R9ve1g	452071
rs17131124	1	91127548	C	G	0.911524	-0.0107991	0.00183144	5.40E-09	Daytime Sleepiness	TRUE	reported	R9ve1g	452071
rs57746981	1	201885234	C	T	0.644468	0.0063061	0.00107274	3.80E-09	Daytime Sleepiness	TRUE	reported	R9ve1g	452071
rs825127	1	223506788	T	G	0.530794	0.00577086	0.00102973	2.00E-08	Daytime Sleepiness	TRUE	reported	R9ve1g	452071
rs4665972	2	27598097	T	C	0.393644	0.00702837	0.00105545	2.70E-11	Daytime Sleepiness	TRUE	reported	R9ve1g	452071
rs7598712	2	46660452	G	T	0.555254	0.00564357	0.00103885	4.40E-08	Daytime Sleepiness	TRUE	reported	R9ve1g	452071
rs6741951	2	58959112	G	A	0.710788	0.0076996	0.00113785	1.40E-11	Daytime Sleepiness	TRUE	reported	R9ve1g	452071
rs11123962	2	104157011	T	G	0.553234	-0.00760762	0.00103139	1.60E-13	Daytime Sleepiness	TRUE	reported	R9ve1g	452071
rs7607363	2	213402705	A	G	0.561234	-0.00654917	0.0010354	3.10E-10	Daytime Sleepiness	TRUE	reported	R9ve1g	452071
rs13010456	2	236792801	A	G	0.594693	0.00734809	0.00104819	2.90E-12	Daytime Sleepiness	TRUE	reported	R9ve1g	452071
rs13097760	3	82823561	A	C	0.638696	-0.00592699	0.0010746	4.10E-08	Daytime Sleepiness	TRUE	reported	R9ve1g	452071
rs9821366	3	84561063	A	G	0.271013	0.00771163	0.00115503	3.50E-11	Daytime Sleepiness	TRUE	reported	R9ve1g	452071
rs843372	3	183996213	C	T	0.230053	0.00762125	0.00122243	3.80E-10	Daytime Sleepiness	TRUE	reported	R9ve1g	452071
rs6897863	5	92512481	A	C	0.584415	0.00605866	0.00104426	6.90E-09	Daytime Sleepiness	TRUE	reported	R9ve1g	452071
rs12153518	5	138501494	A	G	0.47224	0.00679561	0.00102863	3.30E-11	Daytime Sleepiness	TRUE	reported	R9ve1g	452071
rs6923811	6	27289776	T	C	0.678973	0.00687507	0.0011019	4.50E-10	Daytime Sleepiness	TRUE	reported	R9ve1g	452071
rs55960940	6	38153146	T	C	0.822172	0.00755467	0.00134768	2.30E-08	Daytime Sleepiness	TRUE	reported	R9ve1g	452071
rs3122163	6	55056368	C	T	0.231114	0.00920223	0.00122324	3.40E-14	Daytime Sleepiness	TRUE	reported	R9ve1g	452071
rs641498	6	124911565	A	G	0.392184	-0.00587812	0.00105586	2.50E-08	Daytime Sleepiness	TRUE	reported	R9ve1g	452071
rs62519825	8	65479707	T	C	0.88741	-0.00893208	0.00162354	2.60E-08	Daytime Sleepiness	TRUE	reported	R9ve1g	452071
rs285793	8	106087862	G	A	0.461246	0.00657609	0.00103235	2.10E-10	Daytime Sleepiness	TRUE	reported	R9ve1g	452071
rs7837226	8	131235895	A	G	0.472852	-0.00570998	0.00102965	2.20E-08	Daytime Sleepiness	TRUE	reported	R9ve1g	452071
rs13284688	9	81727018	T	C	0.793399	-0.0101903	0.00126801	1.10E-15	Daytime Sleepiness	TRUE	reported	R9ve1g	452071
rs1566362	9	128163132	T	C	0.631731	0.00644926	0.00106555	1.60E-09	Daytime Sleepiness	TRUE	reported	R9ve1g	452071
rs7476897	10	92416402	G	A	0.679434	0.00732039	0.0010987	4.60E-11	Daytime Sleepiness	TRUE	reported	R9ve1g	452071
rs12253139	10	128899947	T	C	0.876007	-0.00851903	0.00155596	4.40E-08	Daytime Sleepiness	TRUE	reported	R9ve1g	452071
rs4765939	12	2582397	G	C	0.583272	-0.0062379	0.00104294	2.30E-09	Daytime Sleepiness	TRUE	reported	R9ve1g	452071
rs1846644	12	117938380	T	C	0.590849	-0.0113557	0.00104469	1.80E-27	Daytime Sleepiness	TRUE	reported	R9ve1g	452071
rs7982022	13	54049003	G	A	0.562517	-0.0057991	0.00105449	4.40E-08	Daytime Sleepiness	TRUE	reported	R9ve1g	452071
rs8015449	14	82161860	A	G	0.538612	0.00597228	0.0010316	6.20E-09	Daytime Sleepiness	TRUE	reported	R9ve1g	452071
rs2472297	15	75027880	C	T	0.734689	0.00637313	0.00116124	4.80E-08	Daytime Sleepiness	TRUE	reported	R9ve1g	452071
rs17356118	15	83237899	A	G	0.76879	-0.00759808	0.00121728	3.20E-10	Daytime Sleepiness	TRUE	reported	R9ve1g	452071
rs7162082	15	83896608	C	T	0.79542	0.0070162	0.00127055	3.50E-08	Daytime Sleepiness	TRUE	reported	R9ve1g	452071
rs11078398	17	17697099	G	A	0.743669	0.00731193	0.00123757	3.80E-09	Daytime Sleepiness	TRUE	reported	R9ve1g	452071
rs2911774	17	43579103	A	G	0.815525	0.00849671	0.00132412	1.60E-10	Daytime Sleepiness	TRUE	reported	R9ve1g	452071
rs147114641	17	43581015	C	A	0.773748	0.00807811	0.0012262	5.30E-11	Daytime Sleepiness	TRUE	reported	R9ve1g	452071
rs62066119	17	43695197	C	T	0.747851	0.00846368	0.00120973	2.80E-12	Daytime Sleepiness	TRUE	reported	R9ve1g	452071
rs55938136	17	43798360	A	G	0.775415	0.00816616	0.00122932	3.60E-11	Daytime Sleepiness	TRUE	reported	R9ve1g	452071
rs200787224	17	44141908	C	CA	0.7747	0.00799825	0.00122872	8.60E-11	Daytime Sleepiness	TRUE	reported	R9ve1g	452071
rs376781756	17	44355767	G	T	0.803841	0.0078134	0.00135392	9.50E-09	Daytime Sleepiness	TRUE	reported	R9ve1g	452071
SNP	chr.exposure	pos.exposure	effect_allele.exposure	other_allele.exposure	eaf.exposure	beta.exposure	se.exposure	pval.exposure	exposure	mr_keep.exposure	pval_origin.exposure	id.exposure	samplesize.exposure
rs4751	1	1686040	G	T	0.575309	-0.00772701	0.00139656	1.60E-08	Insomnia	TRUE	reported	LBDonK	453379
rs12405761	1	57850914	A	C	0.570539	0.00923926	0.00139853	2.60E-11	Insomnia	TRUE	reported	LBDonK	453379
rs11804386	1	87738947	G	A	0.666568	-0.00805764	0.00146423	2.60E-08	Insomnia	TRUE	reported	LBDonK	453379
rs11184946	1	107185225	C	T	0.582984	-0.00878653	0.00139968	2.90E-10	Insomnia	TRUE	reported	LBDonK	453379
rs6664467	1	151738403	G	A	0.86353	0.0112654	0.00201555	4.50E-08	Insomnia	TRUE	reported	LBDonK	453379
rs2644128	1	201793440	C	G	0.449554	-0.0097367	0.00138698	1.00E-12	Insomnia	TRUE	reported	LBDonK	453379
rs35881094	2	58922921	T	G	0.573415	-0.0110528	0.00140168	3.00E-15	Insomnia	TRUE	reported	LBDonK	453379
rs113851554	2	66750564	G	T	0.942793	-0.0413744	0.00307399	1.30E-41	Insomnia	TRUE	reported	LBDonK	453379
rs62158170	2	114082175	A	G	0.785192	0.0120517	0.00168292	5.70E-13	Insomnia	TRUE	reported	LBDonK	453379
rs4577309	2	191288833	A	G	0.46879	0.00811816	0.00138836	3.70E-09	Insomnia	TRUE	reported	LBDonK	453379
rs4688760	3	49980596	C	T	0.309505	-0.0107674	0.00149946	1.00E-12	Insomnia	TRUE	reported	LBDonK	453379
rs9845387	3	116425935	C	A	0.959258	0.0196529	0.00350129	2.10E-08	Insomnia	TRUE	reported	LBDonK	453379
rs1841625	4	91287477	A	G	0.568289	-0.00771112	0.00139836	3.60E-08	Insomnia	TRUE	reported	LBDonK	453379
rs11097861	4	105330133	A	G	0.284352	-0.00916107	0.00153452	1.70E-09	Insomnia	TRUE	reported	LBDonK	453379
rs1430205	5	87678585	C	T	0.541669	-0.00769252	0.0013907	3.60E-08	Insomnia	TRUE	reported	LBDonK	453379
rs28061	5	102543878	A	G	0.692321	0.00836258	0.00150548	2.10E-08	Insomnia	TRUE	reported	LBDonK	453379
rs1592757	5	103889998	G	C	0.644161	-0.00903752	0.00144358	4.60E-10	Insomnia	TRUE	reported	LBDonK	453379
rs7711696	5	135486536	G	T	0.695476	-0.0101773	0.00150096	9.90E-12	Insomnia	TRUE	reported	LBDonK	453379
rs10947690	6	37631768	A	G	0.738436	-0.00868595	0.0015737	3.50E-08	Insomnia	TRUE	reported	LBDonK	453379
rs6932158	6	101246010	T	C	0.509426	-0.00776019	0.00138432	2.80E-08	Insomnia	TRUE	reported	LBDonK	453379
rs314280	6	105400837	A	G	0.453284	-0.00863865	0.00138926	3.50E-10	Insomnia	TRUE	reported	LBDonK	453379
rs3824081	7	1024581	T	C	0.475638	0.00801355	0.0013892	1.10E-08	Insomnia	TRUE	reported	LBDonK	453379
rs6593005	7	52584625	A	G	0.258967	-0.00903102	0.00157978	8.60E-09	Insomnia	TRUE	reported	LBDonK	453379
rs10280045	7	114076394	C	G	0.427157	-0.00906781	0.00140818	1.00E-10	Insomnia	TRUE	reported	LBDonK	453379
rs17151854	8	10236559	G	T	0.846784	-0.010705	0.00192286	2.40E-08	Insomnia	TRUE	reported	LBDonK	453379
rs10156602	9	96345328	A	G	0.637771	0.0101122	0.00144819	3.40E-12	Insomnia	TRUE	reported	LBDonK	453379
rs2296580	10	104241683	G	T	0.702012	0.0103125	0.00151028	8.70E-12	Insomnia	TRUE	reported	LBDonK	453379
rs2297787	10	104680137	T	A	0.920608	0.0152861	0.00256133	2.10E-09	Insomnia	TRUE	reported	LBDonK	453379
rs79780963	10	104952499	C	T	0.922913	0.0148998	0.00257912	6.30E-09	Insomnia	TRUE	reported	LBDonK	453379
rs10838708	11	47441513	G	A	0.540565	0.00840241	0.00140024	2.50E-09	Insomnia	TRUE	reported	LBDonK	453379
rs324017	12	57487814	A	C	0.294978	0.00967624	0.00151903	1.10E-10	Insomnia	TRUE	reported	LBDonK	453379
rs2956278	12	84698234	A	G	0.785194	-0.00952347	0.00168476	1.30E-08	Insomnia	TRUE	reported	LBDonK	453379
rs68094047	12	109855201	C	T	0.749565	-0.00941756	0.00160041	3.30E-09	Insomnia	TRUE	reported	LBDonK	453379
rs1923770	13	53786568	T	A	0.382709	0.00996266	0.00142375	2.30E-12	Insomnia	TRUE	reported	LBDonK	453379
rs1031654	13	54382035	C	A	0.201391	0.0102622	0.00172522	2.00E-09	Insomnia	TRUE	reported	LBDonK	453379
rs4886140	13	59833519	A	G	0.332401	-0.00924246	0.00148079	3.10E-10	Insomnia	TRUE	reported	LBDonK	453379
rs11635495	15	67804682	T	C	0.485702	-0.00813358	0.00138626	6.80E-09	Insomnia	TRUE	reported	LBDonK	453379
rs4886860	15	74340336	G	C	0.234173	0.0112372	0.00163281	6.10E-12	Insomnia	TRUE	reported	LBDonK	453379
rs17139246	16	6106260	T	C	0.610884	-0.00777532	0.00142927	4.10E-08	Insomnia	TRUE	reported	LBDonK	453379
rs1544637	16	51484837	T	C	0.487251	0.00767466	0.00138704	3.00E-08	Insomnia	TRUE	reported	LBDonK	453379
rs3104778	16	52633652	A	G	0.589631	0.0078046	0.00141099	4.20E-08	Insomnia	TRUE	reported	LBDonK	453379
rs2062113	16	59476179	T	C	0.429456	0.00889979	0.00140332	1.90E-10	Insomnia	TRUE	reported	LBDonK	453379
rs17669584	17	28899614	A	G	0.80454	-0.00964647	0.00178391	3.60E-08	Insomnia	TRUE	reported	LBDonK	453379
rs9894577	17	43223292	G	A	0.682406	-0.0123638	0.00148745	1.50E-16	Insomnia	TRUE	reported	LBDonK	453379
rs1942262	18	52873317	G	A	0.707539	-0.0111508	0.00152288	1.10E-13	Insomnia	TRUE	reported	LBDonK	453379
rs11673344	19	37684966	A	G	0.619812	-0.00868506	0.00142888	9.00E-10	Insomnia	TRUE	reported	LBDonK	453379
SNP	chr.exposure	pos.exposure	effect_allele.exposure	other_allele.exposure	eaf.exposure	beta.exposure	se.exposure	pval.exposure	exposure	mr_keep.exposure	pval_origin.exposure	id.exposure	samplesize.exposure
rs7534398	1	7767464	T	A	0.798618	-0.00507915	0.000908499	2.10E-08	Long	TRUE	reported	M6bfXO	339926
rs6737318	2	114083120	A	G	0.778159	-0.00638002	0.000876506	3.40E-13	Long	TRUE	reported	M6bfXO	339926
rs10899257	11	76415209	G	A	0.855527	-0.00563809	0.00103128	4.60E-08	Long	TRUE	reported	M6bfXO	339926
rs75458655	11	118115331	C	T	0.977027	-0.0167308	0.00242295	5.40E-12	Long	TRUE	reported	M6bfXO	339926
rs3751046	11	122828342	A	G	0.852658	-0.00577495	0.00102722	2.00E-08	Long	TRUE	reported	M6bfXO	339926
rs17817288	16	53807764	A	G	0.518127	0.00418824	0.000726521	8.90E-09	Long	TRUE	reported	M6bfXO	339926
rs1724387	17	43674931	T	C	0.771884	0.0057488	0.00087115	3.70E-11	Long	TRUE	reported	M6bfXO	339926
rs55938136	17	43798360	A	G	0.775648	0.00569997	0.000869738	5.00E-11	Long	TRUE	reported	M6bfXO	339926
rs200787224	17	44141908	C	CA	0.77495	0.00566447	0.000869295	6.30E-11	Long	TRUE	reported	M6bfXO	339926
rs2532253	17	44256296	G	A	0.80321	0.00662745	0.000961786	4.80E-12	Long	TRUE	reported	M6bfXO	339926
rs2532353	17	44335867	G	A	0.775722	0.00574	0.000870538	3.80E-11	Long	TRUE	reported	M6bfXO	339926
rs376781756	17	44355767	G	T	0.804029	0.00590287	0.000957765	6.30E-10	Long	TRUE	reported	M6bfXO	339926
SNP	chr.exposure	pos.exposure	effect_allele.exposure	other_allele.exposure	eaf.exposure	beta.exposure	se.exposure	pval.exposure	exposure	mr_keep.exposure	pval_origin.exposure	id.exposure	samplesize.exposure
rs17374439	1	7888438	C	T	0.803508	-0.0555478	0.00566145	1.20E-22	Morning person	TRUE	reported	9QsxSd	403195
rs72645883	1	15928583	C	T	0.844043	-0.0353333	0.00621376	7.00E-09	Morning person	TRUE	reported	9QsxSd	403195
rs1883565	1	20008507	C	T	0.34341	0.0272483	0.00473645	1.70E-08	Morning person	TRUE	reported	9QsxSd	403195
rs3767240	1	21163921	T	C	0.6179	-0.0312547	0.00462739	7.30E-12	Morning person	TRUE	reported	9QsxSd	403195
rs12140153	1	62579891	G	T	0.904974	0.0484879	0.00787657	4.80E-10	Morning person	TRUE	reported	9QsxSd	403195
rs7547493	1	77775233	A	G	0.82194	-0.0582789	0.00587503	2.50E-23	Morning person	TRUE	reported	9QsxSd	403195
rs11588913	1	79963816	G	A	0.601488	0.0252376	0.00459169	2.50E-08	Morning person	TRUE	reported	9QsxSd	403195
rs72720396	1	91191582	A	G	0.769471	-0.0430894	0.00533877	5.30E-16	Morning person	TRUE	reported	9QsxSd	403195
rs4316403	1	96445333	G	A	0.636764	-0.0259009	0.00468504	4.40E-08	Morning person	TRUE	reported	9QsxSd	403195
rs6593604	1	96956769	G	A	0.475882	0.0272344	0.0045089	1.20E-09	Morning person	TRUE	reported	9QsxSd	403195
rs1709409	1	97602726	T	C	0.798628	0.0321744	0.00563015	1.10E-08	Morning person	TRUE	reported	9QsxSd	403195
rs17575798	1	110086451	G	A	0.806886	0.0392352	0.00568886	7.80E-12	Morning person	TRUE	reported	9QsxSd	403195
rs6537834	1	115108870	T	C	0.385278	-0.0252152	0.00462393	3.50E-08	Morning person	TRUE	reported	9QsxSd	403195
rs11587758	1	150327106	G	A	0.604254	-0.0346078	0.00459127	3.80E-14	Morning person	TRUE	reported	9QsxSd	403195
rs72992015	1	150842696	C	T	0.790697	-0.0324541	0.00555303	5.00E-09	Morning person	TRUE	reported	9QsxSd	403195
rs75650221	1	174421994	C	T	0.961665	-0.0646983	0.011744	3.30E-08	Morning person	TRUE	reported	9QsxSd	403195
rs11580135	1	179417780	T	C	0.317102	-0.0275921	0.00483431	1.30E-08	Morning person	TRUE	reported	9QsxSd	403195
rs1144566	1	182569626	T	C	0.0301951	0.170568	0.0131392	2.80E-38	Morning person	TRUE	reported	9QsxSd	403195
rs10196909	2	4651199	C	A	0.481825	0.0321137	0.00450856	6.20E-13	Morning person	TRUE	reported	9QsxSd	403195
rs75120545	2	44271496	C	T	0.969822	-0.0974698	0.013897	2.90E-12	Morning person	TRUE	reported	9QsxSd	403195
rs786406	2	44735953	A	G	0.297822	-0.0327319	0.00492279	1.00E-11	Morning person	TRUE	reported	9QsxSd	403195
rs12713014	2	48986474	A	G	0.942058	0.0577481	0.00972354	2.50E-09	Morning person	TRUE	reported	9QsxSd	403195
rs10495976	2	49750698	A	T	0.609803	-0.0280661	0.00464114	8.00E-10	Morning person	TRUE	reported	9QsxSd	403195
rs7602425	2	50460480	C	T	0.924493	-0.0536212	0.00854512	3.20E-10	Morning person	TRUE	reported	9QsxSd	403195
rs778147	2	61606566	C	A	0.366787	0.0296842	0.00466945	4.60E-10	Morning person	TRUE	reported	9QsxSd	403195
rs6744983	2	76346385	G	T	0.62256	-0.0253244	0.00464336	4.90E-08	Morning person	TRUE	reported	9QsxSd	403195
rs10520176	2	77217310	T	C	0.501844	0.0333663	0.00451125	2.00E-13	Morning person	TRUE	reported	9QsxSd	403195
rs28380327	2	144232491	A	T	0.629168	0.0289857	0.0046586	7.80E-10	Morning person	TRUE	reported	9QsxSd	403195
rs1947198	2	149390435	C	T	0.87783	-0.0396714	0.00686993	3.20E-09	Morning person	TRUE	reported	9QsxSd	403195
rs11679484	2	198921604	C	A	0.626068	-0.0298469	0.00467197	1.60E-10	Morning person	TRUE	reported	9QsxSd	403195
rs184033703	2	206956138	G	A	0.942004	-0.061814	0.00966021	2.20E-10	Morning person	TRUE	reported	9QsxSd	403195
rs114769095	2	239140025	G	C	0.973274	-0.112803	0.0142967	1.60E-15	Morning person	TRUE	reported	9QsxSd	403195
rs960783	2	239149570	C	T	0.955432	0.0930914	0.0109798	3.20E-17	Morning person	TRUE	reported	9QsxSd	403195
rs77008212	2	239307113	A	G	0.912884	0.0825698	0.00798588	1.10E-24	Morning person	TRUE	reported	9QsxSd	403195
rs34581681	3	2435647	G	A	0.839317	0.034486	0.00617986	4.20E-08	Morning person	TRUE	reported	9QsxSd	403195
rs149611468	3	8817423	T	C	0.98802	0.119935	0.0211151	1.30E-08	Morning person	TRUE	reported	9QsxSd	403195
rs9853258	3	18154519	T	C	0.450075	0.0297334	0.00455373	6.40E-11	Morning person	TRUE	reported	9QsxSd	403195
rs7428484	3	23330101	A	G	0.673726	0.0264805	0.00479571	2.40E-08	Morning person	TRUE	reported	9QsxSd	403195
rs13059636	3	24935790	A	G	0.528035	-0.0317863	0.0045329	3.50E-12	Morning person	TRUE	reported	9QsxSd	403195
rs115774037	3	36834668	T	C	0.973872	-0.078752	0.0140393	3.50E-08	Morning person	TRUE	reported	9QsxSd	403195
rs11712056	3	49914397	T	C	0.556466	0.031956	0.0045329	3.70E-12	Morning person	TRUE	reported	9QsxSd	403195
rs60194061	3	77301121	G	A	0.73032	-0.0326372	0.00511118	8.60E-11	Morning person	TRUE	reported	9QsxSd	403195
rs957501	3	85791657	T	A	0.336033	0.0263167	0.00477453	3.00E-08	Morning person	TRUE	reported	9QsxSd	403195
rs13065394	3	132971327	G	T	0.711759	0.0297983	0.00497014	2.20E-09	Morning person	TRUE	reported	9QsxSd	403195
rs2102506	3	156342849	G	A	0.3605	0.0293301	0.00472786	8.30E-10	Morning person	TRUE	reported	9QsxSd	403195
rs9831488	3	160755062	A	G	0.649417	-0.0278255	0.00476992	4.90E-09	Morning person	TRUE	reported	9QsxSd	403195
rs3850174	3	172364093	T	A	0.742864	0.0318495	0.00519636	1.40E-09	Morning person	TRUE	reported	9QsxSd	403195
rs2239626	3	185998884	T	C	0.695468	-0.0335827	0.00490904	5.00E-12	Morning person	TRUE	reported	9QsxSd	403195
rs73182221	3	186079261	G	A	0.886379	-0.045697	0.0071821	3.00E-10	Morning person	TRUE	reported	9QsxSd	403195
rs34627176	4	1006200	G	A	0.786135	-0.0298813	0.00551285	3.60E-08	Morning person	TRUE	reported	9QsxSd	403195
rs231398	4	2832189	G	A	0.837855	0.0354509	0.00615616	5.80E-09	Morning person	TRUE	reported	9QsxSd	403195
rs12498561	4	21860763	C	G	0.495316	-0.0246853	0.00451296	4.60E-08	Morning person	TRUE	reported	9QsxSd	403195
rs17292170	4	62881399	C	A	0.751201	0.0298781	0.00521233	1.20E-08	Morning person	TRUE	reported	9QsxSd	403195
rs7691121	4	83274208	C	G	0.767925	0.0340511	0.00532954	1.00E-10	Morning person	TRUE	reported	9QsxSd	403195
rs4339281	4	118315560	A	G	0.872986	0.0394057	0.00677257	5.30E-09	Morning person	TRUE	reported	9QsxSd	403195
rs4241964	4	137053959	T	G	0.525037	-0.0326407	0.00453345	7.20E-13	Morning person	TRUE	reported	9QsxSd	403195
rs10067113	5	26016363	C	T	0.380528	-0.0280609	0.00465694	2.50E-09	Morning person	TRUE	reported	9QsxSd	403195
rs7701529	5	63861475	A	T	0.237701	-0.0300077	0.00532109	1.70E-08	Morning person	TRUE	reported	9QsxSd	403195
rs34875688	5	88182026	T	A	0.767268	0.0320299	0.00534073	1.10E-09	Morning person	TRUE	reported	9QsxSd	403195
rs1976423	5	104042643	A	C	0.503965	-0.0290428	0.00451308	1.70E-10	Morning person	TRUE	reported	9QsxSd	403195
rs1027742	5	115904410	A	G	0.738161	0.0306919	0.00514017	3.60E-09	Morning person	TRUE	reported	9QsxSd	403195
rs2910032	5	152540354	C	T	0.482294	-0.0336963	0.00451163	1.10E-13	Morning person	TRUE	reported	9QsxSd	403195
rs10074911	5	175337736	C	A	0.232273	0.0321941	0.0057679	3.00E-08	Morning person	TRUE	reported	9QsxSd	403195
rs9369915	6	13191447	G	A	0.694286	-0.0371942	0.00491617	3.00E-14	Morning person	TRUE	reported	9QsxSd	403195
rs2842638	6	41583576	T	G	0.576697	0.0306548	0.0046083	1.90E-11	Morning person	TRUE	reported	9QsxSd	403195
rs3857599	6	50938247	C	A	0.836792	-0.0342886	0.00609809	1.20E-08	Morning person	TRUE	reported	9QsxSd	403195
rs73434034	6	54418958	A	G	0.890543	-0.0440083	0.00724376	1.40E-09	Morning person	TRUE	reported	9QsxSd	403195
rs2653343	6	55133326	T	A	0.214721	0.0613434	0.00549376	5.20E-29	Morning person	TRUE	reported	9QsxSd	403195
rs60616179	6	110244765	A	G	0.945063	0.0562524	0.009965	9.20E-09	Morning person	TRUE	reported	9QsxSd	403195
rs77280203	6	153110829	A	C	0.989973	-0.174368	0.0228214	1.50E-14	Morning person	TRUE	reported	9QsxSd	403195
rs520954	6	153135378	A	G	0.672827	-0.0422581	0.00479665	1.60E-18	Morning person	TRUE	reported	9QsxSd	403195
rs9365769	6	165186963	A	G	0.436791	0.0262836	0.00457414	7.30E-09	Morning person	TRUE	reported	9QsxSd	403195
rs7779115	7	24086346	T	C	0.725449	0.0287757	0.00505208	1.10E-08	Morning person	TRUE	reported	9QsxSd	403195
rs12669911	7	32262377	A	C	0.384427	-0.0294686	0.00466454	1.80E-10	Morning person	TRUE	reported	9QsxSd	403195
rs56049037	7	32947201	G	A	0.712681	0.0291023	0.00499918	1.00E-08	Morning person	TRUE	reported	9QsxSd	403195
rs6967481	7	50642701	C	T	0.50325	-0.029923	0.00452547	1.90E-11	Morning person	TRUE	reported	9QsxSd	403195
rs4729303	7	96474878	C	T	0.186957	-0.0424076	0.00577473	2.30E-13	Morning person	TRUE	reported	9QsxSd	403195
rs202157	7	101637753	C	T	0.29887	0.0397113	0.00493645	1.30E-15	Morning person	TRUE	reported	9QsxSd	403195
rs57307671	7	102086605	A	T	0.874122	0.0415585	0.00684982	8.60E-10	Morning person	TRUE	reported	9QsxSd	403195
rs4729854	7	102383663	T	A	0.516577	0.0443063	0.00461134	1.90E-21	Morning person	TRUE	reported	9QsxSd	403195
rs1524472	7	115676877	A	G	0.440956	0.0252952	0.00453478	2.10E-08	Morning person	TRUE	reported	9QsxSd	403195
rs13269289	8	4843924	G	A	0.673433	-0.0275791	0.00485097	5.50E-09	Morning person	TRUE	reported	9QsxSd	403195
rs11786306	8	8208759	G	C	0.646069	-0.0292175	0.00474691	7.00E-10	Morning person	TRUE	reported	9QsxSd	403195
rs13255030	8	31671330	A	G	0.58516	0.026079	0.00457953	1.50E-08	Morning person	TRUE	reported	9QsxSd	403195
rs7001604	8	33680979	T	C	0.613053	-0.0318374	0.00465169	7.30E-12	Morning person	TRUE	reported	9QsxSd	403195
rs2590466	8	34264770	C	T	0.192206	-0.0327751	0.00576679	1.40E-08	Morning person	TRUE	reported	9QsxSd	403195
rs769066	8	53145698	T	C	0.816231	-0.0329101	0.00583263	1.90E-08	Morning person	TRUE	reported	9QsxSd	403195
rs4321976	8	76614958	T	C	0.778752	0.0328285	0.00543138	8.70E-10	Morning person	TRUE	reported	9QsxSd	403195
rs1470764	8	101950038	G	A	0.387169	0.0285814	0.00463098	5.90E-10	Morning person	TRUE	reported	9QsxSd	403195
rs12682033	8	116701130	T	C	0.240285	0.034887	0.00529299	6.10E-11	Morning person	TRUE	reported	9QsxSd	403195
rs7023881	9	8146706	T	C	0.753087	-0.029056	0.00521899	3.90E-08	Morning person	TRUE	reported	9QsxSd	403195
rs10976942	9	8310813	C	A	0.917117	-0.0493619	0.00817039	1.00E-09	Morning person	TRUE	reported	9QsxSd	403195
rs10977117	9	8451755	T	C	0.533984	-0.0255406	0.00453687	1.20E-08	Morning person	TRUE	reported	9QsxSd	403195
rs308521	9	37367094	T	C	0.602903	0.0279091	0.00461881	1.50E-09	Morning person	TRUE	reported	9QsxSd	403195
rs62553781	9	76679777	C	T	0.965073	0.0828273	0.0123021	3.00E-11	Morning person	TRUE	reported	9QsxSd	403195
rs295268	9	86429305	T	C	0.744235	-0.0292561	0.0051628	7.00E-09	Morning person	TRUE	reported	9QsxSd	403195
rs10123584	9	115202610	G	A	0.292722	0.0270426	0.00495784	4.00E-08	Morning person	TRUE	reported	9QsxSd	403195
rs10818834	9	126317324	T	C	0.733086	0.0323011	0.00511442	4.20E-10	Morning person	TRUE	reported	9QsxSd	403195
rs10988239	9	131943440	C	T	0.487682	0.0282008	0.00458247	1.10E-09	Morning person	TRUE	reported	9QsxSd	403195
rs34619169	9	139327277	G	A	0.692101	-0.0272806	0.00488632	1.50E-08	Morning person	TRUE	reported	9QsxSd	403195
rs28458909	9	140257189	C	T	0.876461	0.0676497	0.00683765	4.70E-23	Morning person	TRUE	reported	9QsxSd	403195
rs2893787	10	60568697	G	A	0.2553	0.0288453	0.00516506	3.50E-08	Morning person	TRUE	reported	9QsxSd	403195
rs4752593	10	123536490	G	C	0.371575	0.0265676	0.00468542	9.80E-09	Morning person	TRUE	reported	9QsxSd	403195
rs6599694	10	125124884	G	T	0.656185	0.0266711	0.00476984	2.10E-08	Morning person	TRUE	reported	9QsxSd	403195
rs9664044	10	126710791	C	T	0.767248	0.0300498	0.00533885	2.80E-08	Morning person	TRUE	reported	9QsxSd	403195
rs72632979	11	16615883	A	G	0.828665	0.0346051	0.00599706	4.00E-09	Morning person	TRUE	reported	9QsxSd	403195
rs10501087	11	27670108	T	C	0.799452	-0.0346935	0.00562434	5.40E-10	Morning person	TRUE	reported	9QsxSd	403195
rs640245	11	30393360	C	T	0.516254	-0.0274758	0.00452333	1.00E-09	Morning person	TRUE	reported	9QsxSd	403195
rs11032362	11	33759092	G	A	0.909159	-0.0672744	0.0078366	7.30E-18	Morning person	TRUE	reported	9QsxSd	403195
rs11229543	11	58370698	G	A	0.759944	0.033727	0.00527519	1.10E-10	Morning person	TRUE	reported	9QsxSd	403195
rs4936291	11	114009982	A	G	0.610604	-0.0276435	0.00474862	9.40E-09	Morning person	TRUE	reported	9QsxSd	403195
rs7130530	11	115149728	T	C	0.807669	0.0327187	0.00571333	1.60E-08	Morning person	TRUE	reported	9QsxSd	403195
rs73606718	11	122814474	G	A	0.879064	0.040119	0.00691498	5.70E-09	Morning person	TRUE	reported	9QsxSd	403195
rs2467109	12	23064038	T	A	0.718089	-0.0276799	0.00501668	3.90E-08	Morning person	TRUE	reported	9QsxSd	403195
rs7302062	12	24065752	T	C	0.55303	0.0317771	0.00453388	5.20E-12	Morning person	TRUE	reported	9QsxSd	403195
rs7308565	12	34018179	C	T	0.599394	-0.0319509	0.00464272	6.60E-12	Morning person	TRUE	reported	9QsxSd	403195
rs11181153	12	38289817	C	T	0.413925	0.0384886	0.00457918	6.00E-17	Morning person	TRUE	reported	9QsxSd	403195
rs7313852	12	38908389	G	A	0.564644	0.0477061	0.00455758	3.80E-25	Morning person	TRUE	reported	9QsxSd	403195
rs247929	12	46294908	G	C	0.494236	-0.0247976	0.00451133	3.60E-08	Morning person	TRUE	reported	9QsxSd	403195
rs11834788	12	54595546	A	G	0.531322	0.0247273	0.00458435	4.10E-08	Morning person	TRUE	reported	9QsxSd	403195
rs11174781	12	63516126	T	C	0.877633	0.0475212	0.00687518	3.90E-12	Morning person	TRUE	reported	9QsxSd	403195
rs7959983	12	90452978	T	C	0.595837	-0.0318282	0.00457585	4.70E-12	Morning person	TRUE	reported	9QsxSd	403195
rs7304278	12	106989915	A	G	0.274962	-0.0327762	0.00506843	9.60E-11	Morning person	TRUE	reported	9QsxSd	403195
rs7298532	12	112510404	T	C	0.717936	0.0297328	0.0050014	3.10E-09	Morning person	TRUE	reported	9QsxSd	403195
rs9597241	13	56281271	A	C	0.811186	0.0322479	0.00579356	2.70E-08	Morning person	TRUE	reported	9QsxSd	403195
rs9573971	13	77568732	A	G	0.966149	0.108483	0.0124737	4.90E-18	Morning person	TRUE	reported	9QsxSd	403195
rs11841335	13	94105884	G	A	0.746028	0.0283759	0.00518535	4.00E-08	Morning person	TRUE	reported	9QsxSd	403195
rs10149448	14	84253271	A	G	0.603659	0.0272319	0.00461608	5.60E-09	Morning person	TRUE	reported	9QsxSd	403195
rs12927162	16	52684916	A	G	0.721652	0.0459823	0.00503141	2.00E-20	Morning person	TRUE	reported	9QsxSd	403195
rs1421085	16	53800954	T	C	0.59687	-0.0416273	0.00459528	1.80E-19	Morning person	TRUE	reported	9QsxSd	403195
rs2398144	16	56352854	C	A	0.604904	0.0361429	0.00464362	5.90E-15	Morning person	TRUE	reported	9QsxSd	403195
rs8063159	16	60627491	C	A	0.610647	-0.0307897	0.00462	2.10E-11	Morning person	TRUE	reported	9QsxSd	403195
rs11645898	16	72229532	T	C	0.832897	0.0356701	0.00604233	1.80E-09	Morning person	TRUE	reported	9QsxSd	403195
rs2518022	17	8057367	T	C	0.0835266	0.0626693	0.00813849	7.60E-15	Morning person	TRUE	reported	9QsxSd	403195
rs3760185	17	17401736	C	T	0.751597	0.0371222	0.00527711	2.10E-12	Morning person	TRUE	reported	9QsxSd	403195
rs72829936	17	34060115	G	A	0.836049	-0.0352298	0.00610979	5.50E-09	Morning person	TRUE	reported	9QsxSd	403195
rs35653190	17	46077967	C	T	0.771778	0.0303562	0.00540798	2.00E-08	Morning person	TRUE	reported	9QsxSd	403195
rs117987711	17	57111269	A	C	0.6945	-0.0275361	0.00490408	2.40E-08	Morning person	TRUE	reported	9QsxSd	403195
rs17682747	17	61181112	G	A	0.767481	-0.0305453	0.00534573	8.70E-09	Morning person	TRUE	reported	9QsxSd	403195
rs2949923	17	65475228	A	G	0.538655	-0.0265539	0.0045291	4.20E-09	Morning person	TRUE	reported	9QsxSd	403195
rs2580160	18	1816036	A	G	0.553193	0.0267857	0.00458234	6.80E-09	Morning person	TRUE	reported	9QsxSd	403195
rs62082401	18	5186019	C	G	0.809399	-0.0433691	0.00574168	1.20E-14	Morning person	TRUE	reported	9QsxSd	403195
rs1013987	18	22630836	T	C	0.405487	-0.0269902	0.00460117	4.50E-09	Morning person	TRUE	reported	9QsxSd	403195
rs4239386	18	31664710	T	A	0.665044	0.0343168	0.00478623	4.20E-13	Morning person	TRUE	reported	9QsxSd	403195
rs12969848	18	38152835	C	T	0.470293	-0.0362004	0.00452995	2.60E-15	Morning person	TRUE	reported	9QsxSd	403195
rs9962650	18	53574043	C	G	0.576796	-0.0263905	0.00457013	5.80E-09	Morning person	TRUE	reported	9QsxSd	403195
rs9964420	18	56824041	C	A	0.695679	0.0406117	0.0049227	2.40E-16	Morning person	TRUE	reported	9QsxSd	403195
rs11152350	18	60240352	A	C	0.470982	-0.0279869	0.00451706	6.40E-10	Morning person	TRUE	reported	9QsxSd	403195
rs9636202	19	18449238	G	A	0.733926	0.0281132	0.00512565	2.70E-08	Morning person	TRUE	reported	9QsxSd	403195
rs11670534	19	47003906	C	T	0.834403	0.0328734	0.00608396	4.70E-08	Morning person	TRUE	reported	9QsxSd	403195
rs78095690	20	16239683	T	C	0.564251	-0.0275925	0.00456112	1.10E-09	Morning person	TRUE	reported	9QsxSd	403195
rs2072727	20	43538733	T	C	0.4361	0.0278367	0.0045507	5.60E-10	Morning person	TRUE	reported	9QsxSd	403195
rs139911	22	40704052	C	T	0.42411	0.0377065	0.00457846	2.20E-16	Morning person	TRUE	reported	9QsxSd	403195
rs4822107	22	42704834	G	A	0.494268	-0.0278435	0.00453576	8.70E-10	Morning person	TRUE	reported	9QsxSd	403195
SNP	chr.exposure	pos.exposure	effect_allele.exposure	other_allele.exposure	eaf.exposure	beta.exposure	se.exposure	pval.exposure	exposure	mr_keep.exposure	pval_origin.exposure	id.exposure	samplesize.exposure
rs7524118	1	34736052	T	C	0.291624	-0.0057622	0.00105376	4.90E-08	Short	TRUE	reported	JTYXGj	411934
rs2186122	1	66470206	A	T	0.438434	-0.0056704	0.000972376	4.80E-09	Short	TRUE	reported	JTYXGj	411934
rs12567114	1	98527951	G	A	0.7246	0.00632465	0.00107682	4.10E-09	Short	TRUE	reported	JTYXGj	411934
rs2820313	1	201870221	A	G	0.658888	-0.00600641	0.00101025	2.30E-09	Short	TRUE	reported	JTYXGj	411934
rs1380703	2	57941287	A	G	0.616469	-0.00676427	0.0010046	1.60E-11	Short	TRUE	reported	JTYXGj	411934
rs75539574	2	58871658	A	C	0.914664	0.0111573	0.00172712	8.40E-11	Short	TRUE	reported	JTYXGj	411934
rs2863957	2	114089551	C	A	0.781508	0.0101896	0.0011609	2.60E-18	Short	TRUE	reported	JTYXGj	411934
rs2014830	3	50172397	C	T	0.698128	0.00578646	0.00105038	2.70E-08	Short	TRUE	reported	JTYXGj	411934
rs17005118	4	82288564	G	A	0.735064	-0.00648249	0.00108701	2.50E-09	Short	TRUE	reported	JTYXGj	411934
rs13107325	4	103188709	C	T	0.925472	-0.0132682	0.0018282	2.50E-13	Short	TRUE	reported	JTYXGj	411934
rs12518468	5	7249696	T	C	0.671544	-0.00588504	0.00102098	8.50E-09	Short	TRUE	reported	JTYXGj	411934
rs3776864	5	102327868	A	C	0.66721	0.00572403	0.00101927	1.70E-08	Short	TRUE	reported	JTYXGj	411934
rs4585442	5	135508381	A	G	0.688977	-0.00634685	0.00103625	8.10E-10	Short	TRUE	reported	JTYXGj	411934
rs2517827	6	29832846	C	A	0.688933	-0.00592859	0.00103615	5.70E-09	Short	TRUE	reported	JTYXGj	411934
rs12661667	6	41792545	C	T	0.736505	-0.00602193	0.00108717	2.80E-08	Short	TRUE	reported	JTYXGj	411934
rs9367621	6	55040290	T	A	0.43104	0.00544503	0.000969917	1.60E-08	Short	TRUE	reported	JTYXGj	411934
rs9321171	6	129848635	C	T	0.540122	0.00535425	0.000966062	4.20E-08	Short	TRUE	reported	JTYXGj	411934
rs11763750	7	2080114	G	A	0.814346	0.00721224	0.00123366	5.10E-09	Short	TRUE	reported	JTYXGj	411934
rs1229762	7	114218582	C	T	0.335499	-0.00723933	0.00101656	1.00E-12	Short	TRUE	reported	JTYXGj	411934
rs60882754	8	52886619	A	T	0.938985	0.011304	0.00200145	1.80E-08	Short	TRUE	reported	JTYXGj	411934
rs1607227	11	28808617	G	T	0.704938	0.0063693	0.00105472	1.50E-09	Short	TRUE	reported	JTYXGj	411934
rs7939345	11	47980568	T	G	0.207569	0.00649836	0.0011818	4.00E-08	Short	TRUE	reported	JTYXGj	411934
rs17388803	15	48027204	A	C	0.894352	-0.00982621	0.00158706	6.50E-10	Short	TRUE	reported	JTYXGj	411934
rs59779556	16	56227965	T	G	0.553827	0.00549133	0.000965961	2.00E-08	Short	TRUE	reported	JTYXGj	411934
rs205024	17	11227352	C	T	0.616724	0.00551041	0.000986026	2.70E-08	Short	TRUE	reported	JTYXGj	411934
rs12963463	18	53099093	C	T	0.299425	0.00711437	0.0010601	1.90E-11	Short	TRUE	reported	JTYXGj	411934
rs5757675	22	39838892	G	T	0.259528	0.00645515	0.00109902	2.70E-09	Short	TRUE	reported	JTYXGj	411934
chr.exposure	pos.exposure	SNP	effect_allele.exposure	other_allele.exposure	pval.exposure	samplesize.exposure	eaf.exposure	beta.exposure	se.exposure	exposure	mr_keep.exposure	pval_origin.exposure	id.exposure
1	226805124	rs4653454	A	G	1.90E-07	8433	0.3301	-0.177379389	0.034052387	Single item chronotype	TRUE	reported	HceN2H
4	147764387	rs10029061	T	C	1.17E-06	8433	0.3305	0.165650023	0.034082452	Single item chronotype	TRUE	reported	HceN2H
16	22920604	rs80946	G	A	2.17E-07	8433	0.2531	-0.195390991	0.037692206	Single item chronotype	TRUE	reported	HceN2H
18	4876831	rs62082539	T	C	2.60E-06	8433	0.08158	0.265919476	0.05657614	Single item chronotype	TRUE	reported	HceN2H
chr.exposure	pos.exposure	SNP	effect_allele.exposure	other_allele.exposure	pval.exposure	samplesize.exposure	eaf.exposure	beta.exposure	se.exposure	exposure	mr_keep.exposure	pval_origin.exposure	id.exposure
1	226805124	rs4653454	A	G	1.90E-07	8433	0.3301	-0.177379389	0.034052387	Single item chronotype	TRUE	reported	HceN2H
4	147764387	rs10029061	T	C	1.17E-06	8433	0.3305	0.165650023	0.034082452	Single item chronotype	TRUE	reported	HceN2H
16	22920604	rs80946	G	A	2.17E-07	8433	0.2531	-0.195390991	0.037692206	Single item chronotype	TRUE	reported	HceN2H
18	4876831	rs62082539	T	C	2.60E-06	8433	0.08158	0.265919476	0.05657614	Single item chronotype	TRUE	reported	HceN2H
SNP	chr.exposure	pos.exposure	effect_allele.exposure	other_allele.exposure	eaf.exposure	beta.exposure	se.exposure	pval.exposure	exposure	mr_keep.exposure	pval_origin.exposure	id.exposure	samplesize.exposure
rs915416	1	34731984	C	G	0.289947	0.0192587	0.00249452	9.90E-15	Sleep duration	TRUE	reported	4aVdJN	446118
rs269054	1	57864304	T	A	0.577924	-0.0136431	0.00229327	2.10E-09	Sleep duration	TRUE	reported	4aVdJN	446118
rs61796569	1	66476437	C	T	0.730417	-0.0154442	0.00256443	1.50E-09	Sleep duration	TRUE	reported	4aVdJN	446118
rs12567114	1	98527951	G	A	0.724198	-0.0148307	0.0025403	4.30E-09	Sleep duration	TRUE	reported	4aVdJN	446118
rs62120041	2	9185564	T	C	0.933902	0.0261113	0.00457497	9.60E-09	Sleep duration	TRUE	reported	4aVdJN	446118
rs374153	2	40382712	C	T	0.158085	0.0176119	0.00310308	9.10E-09	Sleep duration	TRUE	reported	4aVdJN	446118
rs2717076	2	58061127	C	T	0.372584	-0.0184107	0.00234091	3.10E-15	Sleep duration	TRUE	reported	4aVdJN	446118
rs75539574	2	58871658	A	C	0.914208	-0.0362482	0.00406522	6.90E-19	Sleep duration	TRUE	reported	4aVdJN	446118
rs72817946	2	59032860	T	C	0.9111	0.0230753	0.00398843	7.10E-09	Sleep duration	TRUE	reported	4aVdJN	446118
rs7556815	2	114085785	G	A	0.780856	-0.0407248	0.00274023	1.30E-49	Sleep duration	TRUE	reported	4aVdJN	446118
rs12611523	2	139195328	A	G	0.545244	0.0126347	0.00227627	3.10E-08	Sleep duration	TRUE	reported	4aVdJN	446118
rs35662245	2	147583187	T	A	0.661256	-0.0145968	0.00239263	1.40E-09	Sleep duration	TRUE	reported	4aVdJN	446118
rs4538155	2	157040773	C	T	0.352574	-0.0129753	0.00237383	3.60E-08	Sleep duration	TRUE	reported	4aVdJN	446118
rs11885663	2	166944004	C	T	0.752191	-0.0162176	0.00261811	8.60E-10	Sleep duration	TRUE	reported	4aVdJN	446118
rs10173260	2	210377845	T	C	0.393765	-0.0128368	0.00231322	2.90E-08	Sleep duration	TRUE	reported	4aVdJN	446118
rs112230981	3	55879269	A	G	0.94984	0.0315275	0.00522787	2.20E-09	Sleep duration	TRUE	reported	4aVdJN	446118
rs17732997	3	70470834	C	G	0.569098	0.0129345	0.0022882	1.20E-08	Sleep duration	TRUE	reported	4aVdJN	446118
rs7644809	3	107564459	T	C	0.421606	0.0130621	0.00230148	1.60E-08	Sleep duration	TRUE	reported	4aVdJN	446118
rs13088093	3	135838598	T	G	0.663683	-0.0162722	0.00240235	7.00E-12	Sleep duration	TRUE	reported	4aVdJN	446118
rs2192528	4	18327896	A	G	0.480065	0.0133687	0.0022692	2.70E-09	Sleep duration	TRUE	reported	4aVdJN	446118
rs17427571	4	82254908	A	G	0.684313	0.0138255	0.00243544	1.30E-08	Sleep duration	TRUE	reported	4aVdJN	446118
rs35531607	4	92533225	T	C	0.525917	-0.0128402	0.00227278	1.50E-08	Sleep duration	TRUE	reported	4aVdJN	446118
rs13109404	4	102896591	T	G	0.928024	0.0312035	0.00440829	1.40E-12	Sleep duration	TRUE	reported	4aVdJN	446118
rs365663	5	1428883	A	G	0.545963	0.0146291	0.00227857	1.00E-10	Sleep duration	TRUE	reported	4aVdJN	446118
rs460692	5	3126584	C	T	0.137484	0.0210557	0.00333164	3.60E-10	Sleep duration	TRUE	reported	4aVdJN	446118
rs56372231	5	102321905	C	T	0.665907	-0.0169439	0.00239981	2.20E-12	Sleep duration	TRUE	reported	4aVdJN	446118
rs11567976	5	137654218	C	T	0.429092	-0.0128042	0.00228516	2.10E-08	Sleep duration	TRUE	reported	4aVdJN	446118
rs151014368	5	176751059	G	A	0.793742	-0.0160924	0.00281958	9.10E-09	Sleep duration	TRUE	reported	4aVdJN	446118
rs34556183	6	28584775	A	G	0.719606	0.0169228	0.00252323	2.30E-11	Sleep duration	TRUE	reported	4aVdJN	446118
rs80193650	6	33464363	A	G	0.837534	-0.0168389	0.00306723	4.10E-08	Sleep duration	TRUE	reported	4aVdJN	446118
rs113113059	6	43160375	T	C	0.78	0.0161406	0.00273732	8.40E-09	Sleep duration	TRUE	reported	4aVdJN	446118
rs9382445	6	54937974	T	C	0.62305	0.014536	0.00233387	4.80E-10	Sleep duration	TRUE	reported	4aVdJN	446118
rs2231265	6	89790201	A	G	0.227711	-0.014955	0.00269917	2.70E-08	Sleep duration	TRUE	reported	4aVdJN	446118
rs9345234	6	93162639	A	C	0.421984	-0.0130117	0.00229893	1.80E-08	Sleep duration	TRUE	reported	4aVdJN	446118
rs34731055	7	2106928	C	T	0.81911	-0.0194603	0.00294778	3.70E-11	Sleep duration	TRUE	reported	4aVdJN	446118
rs2079070	7	114126432	C	G	0.264613	0.0175475	0.00256638	7.50E-12	Sleep duration	TRUE	reported	4aVdJN	446118
rs7806045	7	132610266	T	C	0.754703	0.0147916	0.00262577	1.40E-08	Sleep duration	TRUE	reported	4aVdJN	446118
rs330088	8	9149746	T	C	0.452988	-0.0144687	0.00227678	2.70E-10	Sleep duration	TRUE	reported	4aVdJN	446118
rs73219758	8	14279446	G	A	0.708064	0.0164012	0.00249526	5.60E-11	Sleep duration	TRUE	reported	4aVdJN	446118
rs10973207	9	37100525	G	T	0.842323	-0.0204339	0.00312394	6.00E-11	Sleep duration	TRUE	reported	4aVdJN	446118
rs1776776	9	140497072	T	C	0.873832	0.019963	0.00341071	4.90E-09	Sleep duration	TRUE	reported	4aVdJN	446118
rs12246842	10	21830580	A	G	0.459815	0.0133949	0.00227425	3.90E-09	Sleep duration	TRUE	reported	4aVdJN	446118
rs10761674	10	64618340	C	T	0.477334	0.0123329	0.00226591	4.20E-08	Sleep duration	TRUE	reported	4aVdJN	446118
rs11190970	10	103128332	G	A	0.798661	0.015379	0.00282318	4.60E-08	Sleep duration	TRUE	reported	4aVdJN	446118
rs7915425	10	125016501	T	C	0.174682	0.0190638	0.00298959	2.00E-10	Sleep duration	TRUE	reported	4aVdJN	446118
rs1517572	11	28829882	A	C	0.419464	-0.0146443	0.00229467	1.50E-10	Sleep duration	TRUE	reported	4aVdJN	446118
rs4592416	11	43800474	A	G	0.535593	-0.0146828	0.0022701	9.30E-11	Sleep duration	TRUE	reported	4aVdJN	446118
rs11039544	11	48173412	G	A	0.837779	0.018389	0.00307361	1.90E-09	Sleep duration	TRUE	reported	4aVdJN	446118
rs174560	11	61581764	T	C	0.685785	-0.0135751	0.00243675	2.80E-08	Sleep duration	TRUE	reported	4aVdJN	446118
rs12791153	11	80685181	A	T	0.918911	-0.0235481	0.0042169	1.90E-08	Sleep duration	TRUE	reported	4aVdJN	446118
rs1553132	11	88297740	A	G	0.741567	-0.0145068	0.00258447	2.50E-08	Sleep duration	TRUE	reported	4aVdJN	446118
rs1939455	11	101520886	G	T	0.879446	0.0204253	0.00356118	1.20E-08	Sleep duration	TRUE	reported	4aVdJN	446118
rs1079727	11	113289182	T	C	0.842182	-0.0182922	0.00310347	5.30E-09	Sleep duration	TRUE	reported	4aVdJN	446118
rs7115462	11	113408517	G	A	0.92647	-0.0265639	0.00435799	1.70E-09	Sleep duration	TRUE	reported	4aVdJN	446118
rs7951019	11	118358027	T	G	0.967773	-0.0368792	0.00652085	1.20E-08	Sleep duration	TRUE	reported	4aVdJN	446118
rs3751046	11	122828342	A	G	0.853507	-0.0194129	0.00320818	1.10E-09	Sleep duration	TRUE	reported	4aVdJN	446118
rs34354917	12	38764559	C	A	0.710472	0.0137464	0.00250081	3.90E-08	Sleep duration	TRUE	reported	4aVdJN	446118
rs11067359	12	110025557	G	A	0.748608	0.0142791	0.0026059	4.30E-08	Sleep duration	TRUE	reported	4aVdJN	446118
rs4767550	12	117951150	A	G	0.585862	-0.0143001	0.0023096	6.30E-10	Sleep duration	TRUE	reported	4aVdJN	446118
rs6575005	14	26954078	T	C	0.757854	0.0155637	0.00264172	4.40E-09	Sleep duration	TRUE	reported	4aVdJN	446118
rs10483350	14	29816155	A	G	0.804582	-0.017369	0.0028676	1.50E-09	Sleep duration	TRUE	reported	4aVdJN	446118
rs61985058	14	60233841	C	T	0.856824	-0.0185938	0.00322893	1.30E-08	Sleep duration	TRUE	reported	4aVdJN	446118
rs55658675	14	65554638	C	T	0.644938	0.0131415	0.00236892	2.00E-08	Sleep duration	TRUE	reported	4aVdJN	446118
rs11621908	14	78495761	C	T	0.917141	0.0240951	0.00416298	5.60E-09	Sleep duration	TRUE	reported	4aVdJN	446118
rs8038326	15	47989799	A	G	0.72691	0.0159204	0.0025407	2.80E-10	Sleep duration	TRUE	reported	4aVdJN	446118
rs3095508	16	6550400	C	A	0.593529	0.0153518	0.00230437	3.10E-11	Sleep duration	TRUE	reported	4aVdJN	446118
rs11643715	16	23909538	C	G	0.709058	-0.013895	0.00249658	3.20E-08	Sleep duration	TRUE	reported	4aVdJN	446118
rs9937053	16	53799507	G	A	0.576695	0.0169348	0.00229041	1.20E-13	Sleep duration	TRUE	reported	4aVdJN	446118
rs8050478	16	56120461	G	A	0.500253	0.0160009	0.00226476	1.70E-12	Sleep duration	TRUE	reported	4aVdJN	446118
rs7503199	17	8134275	C	T	0.734267	0.0147449	0.00256383	1.00E-08	Sleep duration	TRUE	reported	4aVdJN	446118
rs205024	17	11227352	C	T	0.616265	-0.0138261	0.00232711	3.90E-09	Sleep duration	TRUE	reported	4aVdJN	446118
rs8072993	17	21335627	T	G	0.363492	-0.0174776	0.00282169	4.20E-10	Sleep duration	TRUE	reported	4aVdJN	446118
rs147114641	17	43581015	C	A	0.773722	0.0159801	0.00270379	3.10E-09	Sleep duration	TRUE	reported	4aVdJN	446118
rs7209792	17	43665090	C	T	0.801655	0.0184074	0.00289468	1.70E-10	Sleep duration	TRUE	reported	4aVdJN	446118
rs55938136	17	43798360	A	G	0.775395	0.0156301	0.00271072	7.20E-09	Sleep duration	TRUE	reported	4aVdJN	446118
rs200787224	17	44141908	C	CA	0.774682	0.0155802	0.00270941	8.10E-09	Sleep duration	TRUE	reported	4aVdJN	446118
rs200412621	17	45464200	T	TA	0.510729	0.0133059	0.0022667	5.10E-09	Sleep duration	TRUE	reported	4aVdJN	446118
rs112252397	17	45506912	A	AG	0.511143	0.013492	0.00227203	3.40E-09	Sleep duration	TRUE	reported	4aVdJN	446118
rs12607679	18	53059748	T	C	0.737717	0.0201387	0.00259284	8.30E-15	Sleep duration	TRUE	reported	4aVdJN	446118
rs10421649	19	9942262	T	A	0.44303	-0.0132975	0.00229462	6.90E-09	Sleep duration	TRUE	reported	4aVdJN	446118
rs2072727	20	43538733	T	C	0.43617	0.0132425	0.00228506	7.90E-09	Sleep duration	TRUE	reported	4aVdJN	446118
chr.exposure	pos.exposure	effect_allele.exposure	other_allele.exposure	samplesize.exposure	beta.exposure	se.exposure	pval.exposure	SNP	exposure	mr_keep.exposure	pval_origin.exposure	id.exposure	eaf.exposure
1	62604065	C	T	336320	0.0064034	0.00116269	3.64E-08	rs2457816	Snoring	TRUE	reported	EOZSpM	NA
1	87772615	G	A	336320	0.00673791	0.00120723	2.39E-08	rs12044462	Snoring	TRUE	reported	EOZSpM	NA
1	96878072	G	A	336320	-0.0125303	0.00205555	1.09E-09	rs12119849	Snoring	TRUE	reported	EOZSpM	NA
2	156999024	C	A	336320	-0.0120009	0.00169202	1.32E-12	rs6732887	Snoring	TRUE	reported	EOZSpM	NA
3	48721040	A	G	336320	0.00810445	0.00147653	4.05E-08	rs990211	Snoring	TRUE	reported	EOZSpM	NA
3	77593064	A	G	336320	0.00696994	0.00115958	1.85E-09	rs9309771	Snoring	TRUE	reported	EOZSpM	NA
4	25408838	G	A	336320	0.00884843	0.00136604	9.34E-11	rs34811474	Snoring	TRUE	reported	EOZSpM	NA
4	42468919	T	G	336320	-0.00807032	0.00135653	2.70E-09	rs78166400	Snoring	TRUE	reported	EOZSpM	NA
5	75003678	T	C	336320	0.00716144	0.00118257	1.40E-09	rs2307111	Snoring	TRUE	reported	EOZSpM	NA
5	90052289	G	A	336320	0.00655156	0.00120086	4.88E-08	rs10062026	Snoring	TRUE	reported	EOZSpM	NA
5	134452529	C	T	336320	0.00684638	0.00123084	2.66E-08	rs4976268	Snoring	TRUE	reported	EOZSpM	NA
6	100827834	A	G	336320	-0.00781563	0.00137762	1.40E-08	rs17060460	Snoring	TRUE	reported	EOZSpM	NA
8	71474355	A	G	336320	-0.00800152	0.00117873	1.14E-11	rs13251292	Snoring	TRUE	reported	EOZSpM	NA
8	78215352	A	G	336320	-0.00741077	0.00127602	6.34E-09	rs7829639	Snoring	TRUE	reported	EOZSpM	NA
9	97567327	A	G	336320	-0.00642789	0.00117004	3.94E-08	rs356131	Snoring	TRUE	reported	EOZSpM	NA
10	9062283	C	T	336320	-0.00847384	0.00147523	9.25E-09	rs2589563	Snoring	TRUE	reported	EOZSpM	NA
11	8579081	T	A	336320	0.0072211	0.00116163	5.09E-10	rs11041980	Snoring	TRUE	reported	EOZSpM	NA
11	27694241	G	C	336320	0.00851046	0.00148006	8.93E-09	rs2049045	Snoring	TRUE	reported	EOZSpM	NA
11	88861590	A	T	336320	0.00683174	0.00121343	1.80E-08	rs11018488	Snoring	TRUE	reported	EOZSpM	NA
12	24022160	C	A	336320	-0.00835637	0.00139481	2.09E-09	rs10505911	Snoring	TRUE	reported	EOZSpM	NA
12	39147635	G	T	336320	0.00796232	0.00140417	1.43E-08	rs34010376	Snoring	TRUE	reported	EOZSpM	NA
12	65791463	C	T	336320	-0.0075484	0.00119985	3.16E-10	rs10878269	Snoring	TRUE	reported	EOZSpM	NA
13	50822363	G	C	336320	-0.00887209	0.0011831	6.44E-14	rs2762049	Snoring	TRUE	reported	EOZSpM	NA
13	51340315	A	G	336320	-0.00911026	0.00116166	4.43E-15	rs592333	Snoring	TRUE	reported	EOZSpM	NA
13	111566732	C	G	336320	0.00672766	0.00119402	1.76E-08	rs7325244	Snoring	TRUE	reported	EOZSpM	NA
14	99743113	G	A	336320	0.00740405	0.00116834	2.34E-10	rs2614464	Snoring	TRUE	reported	EOZSpM	NA
16	1740691	T	C	336320	-0.0121917	0.00219968	2.98E-08	rs9933881	Snoring	TRUE	reported	EOZSpM	NA
16	31054607	C	T	336320	-0.00799837	0.00119375	2.08E-11	rs1108431	Snoring	TRUE	reported	EOZSpM	NA
16	53799279	G	T	336320	-0.00757867	0.00117094	9.66E-11	rs57292959	Snoring	TRUE	reported	EOZSpM	NA
17	1909793	C	T	336320	0.00764884	0.00125101	9.72E-10	rs112176793	Snoring	TRUE	reported	EOZSpM	NA
17	2090341	C	A	336320	0.00675421	0.00117432	8.85E-09	rs4362428	Snoring	TRUE	reported	EOZSpM	NA
17	7559677	G	A	336320	0.00787626	0.00136255	7.45E-09	rs1641511	Snoring	TRUE	reported	EOZSpM	NA
17	43798360	A	G	336320	-0.00777807	0.00138008	1.74E-08	rs55938136	Snoring	TRUE	reported	EOZSpM	NA
17	44866602	T	C	336320	-0.00995638	0.00159169	3.97E-10	rs199497	Snoring	TRUE	reported	EOZSpM	NA
17	46129762	G	A	336320	0.0094467	0.00160241	3.74E-09	rs17680229	Snoring	TRUE	reported	EOZSpM	NA
17	67944404	A	G	336320	-0.00722849	0.00122285	3.40E-09	rs2285569	Snoring	TRUE	reported	EOZSpM	NA

**Table 4:** Forward MR analysis summary table - Association between 10 sleep phenotypes and COPD risk: Mendelian randomization analysis This table presents the Mendelian randomization (MR) analysis results for 10 sleep phenotypes and their association with COPD risk. The analysis used multiple methods: MR Egger, weighted median, weighted mode, simple mode, and inverse variance weighted (multiplicative random effects). For each phenotype, the odds ratio (OR), standard error (SE), 95% confidence intervals (CI), p-values, and the number of SNPs (nsnp) are provided. FDR: False Discovery Rate, a statistical method for correcting for multiple testing to control the proportion of false positive findings.

exposure	outcome	method	nsnp	or	se	or_lci95	or_uci95	pval	FDR
Chronotype	COPD	MR Egger	196	1.082448085	0.183566847	0.755356242	1.551180479	0.666520229	0.674451507
		Weighted median		0.980261771	0.072005392	0.85123572	1.128844946	0.781885296	
		Weighted mode		1.062107714	0.200486502	0.716986548	1.573352806	0.764081174	
		Simple mode		0.740900912	0.234433547	0.467957343	1.173043165	0.202345231	
		Inverse variance weighted (multiplicative random effects)		1.00097187	0.056236142	0.896504972	1.117611966	0.986218383	
Daytime napping	COPD	MR Egger	107	1.214350843	0.665079865	0.32977427	4.471688977	0.770855521	0.03266594
		Weighted median		1.485659942	0.225857648	0.954257326	2.312987706	0.079654748	
		Weighted mode		1.353171522	0.586264124	0.428859221	4.269636932	0.607001723	
		Simple mode		1.666387671	0.617967201	0.496308534	5.595003275	0.410460813	
		Inverse variance weighted (multiplicative random effects)		1.577336689	0.189322506	1.08835314	2.286014473	0.016075213	
Daytime Sleepiness	COPD	MR Egger	38	0.665907795	1.789509354	0.019959653	22.21647808	0.821541689	0.662133792
		Weighted median		1.25429142	0.447692914	0.521572337	3.016354306	0.612796762	
		Weighted mode		0.773350819	0.947229242	0.120803284	4.950788328	0.787636683	
		Simple mode		1.004187292	1.005250188	0.140000145	7.202793366	0.996705753	
		Inverse variance weighted (multiplicative random effects)		1.032412746	0.378905151	0.49127287	2.16962129	0.932908495	
Insomnia	COPD	MR Egger	41	2.075598426	0.824997788	0.41199414	10.45672356	0.381499019	0.000309246
		Weighted median		1.508345898	0.302220286	0.834153249	2.727445287	0.173836175	
		Weighted mode		1.198550933	0.574487812	0.388725111	3.695476049	0.754202827	
		Simple mode		1.681953336	0.744497335	0.390917414	7.2367383	0.488968701	
		Inverse variance weighted (fixed effects)		2.288191929	0.203038522	1.53696002	3.406609303	4.56457E-05	
Long sleep duration	COPD	MR Egger	11	0.327990265	5.732376303	4.32876E-06	24851.81834	0.850127252	0.580262419
		Weighted median		0.773258272	1.4157615	0.04821743	12.40066835	0.85587454	
		Weighted mode		0.793644633	1.703151015	0.028175697	22.35514522	0.894750371	
		Simple mode		0.68861168	2.044895312	0.012511931	37.89870886	0.85888027	
		Inverse variance weighted (multiplicative random effects)		0.519968574	1.477759185	0.028713298	9.416101039	0.658089017	
Morning person	COPD	MR Egger	148	0.964814812	0.11228218	0.774225446	1.202321141	0.750175136	0.574255226
		Weighted median		0.953490487	0.049068512	0.866060843	1.049746234	0.331748189	
		Weighted mode		0.911809561	0.103860152	0.743869233	1.117665094	0.375494466	
		Simple mode		0.966494295	0.132393037	0.745596854	1.252836861	0.797218816	
		Inverse variance weighted (multiplicative random effects)		0.981965452	0.039156219	0.909422641	1.060294856	0.642086715	
Short sleep duration	COPD	MR Egger	25	21.64433925	2.628568209	0.125274276	3739.613876	0.254084638	0.032452701
		Weighted median		2.442641161	0.660685425	0.669072044	8.917568587	0.176456092	
		Weighted mode		0.857956474	1.599515538	0.037319012	19.72424409	0.924490427	
		Simple mode		27.52575468	1.730755679	0.92574262	818.4425718	0.067428509	
		Inverse variance weighted (multiplicative random effects)		3.661743223	0.537413641	1.277121334	10.4988955	0.015728348	
Single item chronotype	COPD	MR Egger	4	0.929000429	0.225465233	0.597167321	1.445226097	0.774955028	0.655661124
		Weighted median		1.009822624	0.041061756	0.93173546	1.094454141	0.811843351	
		Weighted mode		1.031806659	0.061002896	0.915527961	1.162853595	0.643166709	
		Simple mode		1.030267054	0.058061223	0.919447849	1.154443076	0.642988793	
		Inverse variance weighted (fixed effects)		1.004139265	0.035140067	0.937307562	1.075736187	0.906424068	
Sleep duration	COPD	MR Egger	74	1.22307067	0.548305598	0.417565303	3.582438135	0.714510598	0.545499284
		Weighted median		0.999029031	0.173964372	0.710389684	1.404945803	0.995544529	
		Weighted mode		0.891867023	0.299745135	0.495623507	1.604901252	0.703729847	
		Simple mode		0.672996804	0.433726505	0.287618984	1.574738539	0.364221082	
		Inverse variance weighted (multiplicative random effects)		0.922957678	0.141628137	0.699238743	1.218254686	0.571343993	
Snoring	COPD	MR Egger	31	440.3605582	2.295041439	4.900379356	39571.92028	0.012821598	0.030848821
		Weighted median		2.18263103	0.466424132	0.874887252	5.445133872	0.09424145	
		Weighted mode		1.924204422	0.914682919	0.320374093	11.55699771	0.479794365	
		Simple mode		2.168522896	0.97986848	0.317748218	14.79942699	0.435757327	
		Inverse variance weighted (multiplicative random effects)		2.580354847	0.383406907	1.217073722	5.470688439	0.013421739	

**Table 5:** Summary of reverse MR analysis - Association between COPD and 10 sleep phenotypes: reverse Mendelian randomization analysis This table presents the results of reverse Mendelian randomization (MR) analysis to investigate the causal effect of chronic obstructive pulmonary sisease (COPD) on 10 distinct sleep phenotypes (including chronotype, daytime napping, daytime sleepiness, insomnia, long and short sleep durations, and others). The analysis utilized multiple statistical methods: MR Egger, weighted median, weighted mode, simple mode, and inverse variance weighted (fixed or multiplicative random effects). For each sleep phenotype, the odds ratio (OR), standard error (SE), 95% confidence intervals (CI), p-values, and the number of SNPs (nsnp) used are provided. These results assess the potential causal influence of COPD on various sleep behaviors and patterns.

exposure	outcome	method	nsnp	or	se	or_lci95	or_uci95	pval
COPD	Chronotype	MR Egger	9	0.989051436	0.058408321	0.882065247	1.109014041	0.855846405
		Weighted median		1.039047746	0.021302428	0.996557844	1.083349276	0.07215544
		Weighted mode		1.037992766	0.028045878	0.982474288	1.096648529	0.220322462
		Simple mode		1.03745813	0.031304499	0.975716504	1.103106658	0.273890363
		Inverse variance weighted (fixed effects)		1.018853006	0.015458055	0.988446958	1.050194389	0.226943754
COPD	Daytime napping	MR Egger	14	1.02502301	0.010191186	1.00475159	1.045703418	0.032015457
		Weighted median		1.012462596	0.005202849	1.002190388	1.022840091	0.017287404
		Weighted mode		1.010730809	0.005077616	1.000721776	1.020839951	0.055600526
		Simple mode		1.010730809	0.009412226	0.992255821	1.029549786	0.277266039
		Inverse variance weighted (fixed effects)		1.009613813	0.003695197	1.002328014	1.016952571	0.009617726
COPD	Daytime sleepiness	MR Egger	11	0.997534666	0.017789659	0.963352289	1.032929927	0.892700025
		Weighted median		0.987933987	0.006285783	0.975837177	1.000180753	0.053452546
		Weighted mode		0.987295485	0.006444667	0.97490285	0.999845652	0.075375539
		Simple mode		0.986284412	0.013140107	0.961207352	1.01201571	0.31798452
		Inverse variance weighted (multiplicative random effects)		0.996800134	0.005968339	0.985207559	1.008529115	0.59126775
COPD	Insomnia	MR Egger	10	1.021623589	0.021826102	0.978841017	1.066276076	0.35571375
		Weighted median		1.005745422	0.008405597	0.989311541	1.022452293	0.495512652
		Weighted mode		1.003812846	0.009389465	0.985508275	1.022457402	0.694718751
		Simple mode		1.018205179	0.016816882	0.985191107	1.052325564	0.31127489
		Inverse variance weighted (fixed effects)		1.009689919	0.005889011	0.998102614	1.021411745	0.101525422
COPD	Long sleep duration	MR Egger	11	0.994853465	0.008614494	0.978196966	1.011793587	0.563963251
		Weighted median		0.99746504	0.003972538	0.989728759	1.005261791	0.522867681
		Weighted mode		0.99708236	0.004382503	0.988554394	1.005683895	0.520036503
		Simple mode		0.996480996	0.005320369	0.986143761	1.006926591	0.522572164
		Inverse variance weighted (fixed effects)		1.000015267	0.003045564	0.994063652	1.006002515	0.996000322
COPD	Morning person	MR Egger	9	0.933386661	0.095093166	0.774669849	1.124621876	0.49201121
		Weighted median		1.032573364	0.036508213	0.961268031	1.109168012	0.379945408
		Weighted mode		1.027541502	0.053961271	0.924414205	1.142173641	0.628186977
		Simple mode		1.018110395	0.053009452	0.917639956	1.129581128	0.743635468
		Inverse variance weighted (fixed effects)		1.015281907	0.025911216	0.965007247	1.068175762	0.55833355
COPD	Short sleep duration	MR Egger	11	1.014279518	0.017370488	0.980328431	1.049406412	0.435432578
		Weighted median		1.001369611	0.005398634	0.990829658	1.012021683	0.799864605
		Weighted mode		1.00145779	0.005998115	0.989753282	1.013300711	0.813019428
		Simple mode		1.002540166	0.010043017	0.982998812	1.022469989	0.805687801
		Inverse variance weighted (multiplicative random effects)		1.007024211	0.005890173	0.995465229	1.01871741	0.234690973
COPD	Single-item chronotype	MR Egger	43	0.809005622	0.230818997	0.514605524	1.271828745	0.363858858
		Weighted median		0.906484863	0.114891756	0.72370691	1.135424846	0.392799367
		Weighted mode		0.9016828	0.104529039	0.734643891	1.106702012	0.32780321
		Simple mode		1.201265224	0.241084335	0.748899906	1.926877179	0.451128917
		Inverse variance weighted (multiplicative random effects)		1.001492644	0.092346924	0.835680898	1.200203951	0.987113619
COPD	Sleep duration	MR Egger	10	1.005566809	0.03922308	0.93115841	1.085921145	0.890947873
		Weighted median		0.991346311	0.013978826	0.964553626	1.018883223	0.534105557
		Weighted mode		0.993315696	0.013616426	0.967156517	1.020182416	0.634124262
		Simple mode		0.980940355	0.022893458	0.937897323	1.025958765	0.422356237
		Inverse variance weighted (fixed effects)		0.984605161	0.00995402	0.965581808	1.004003301	0.119085501
COPD	Snoring	MR Egger	12	0.999451113	0.014831143	0.970816239	1.028930591	0.971198236
		Weighted median		0.999838095	0.006071901	0.988009621	1.01180818	0.978725525
		Weighted mode		0.999026716	0.00658619	0.986213229	1.012006683	0.885137643
		Simple mode		1.00045098	0.009363606	0.982257496	1.018981446	0.962458062
		Inverse variance weighted (fixed effects)		1.003769661	0.004623067	0.994715376	1.012906362	0.41571959

**Table 6:** Summary of the sensitivity analysis of the causal effect of COPD on 10 sleep phenotypes This table presents the results of a sensitivity analysis for the reverse Mendelian randomization (MR) examining the causal effect of chronic obstructive pulmonary disease (COPD) on 10 distinct sleep phenotypes (including chronotype, daytime napping, daytime sleepiness, insomnia, sleep duration, and others). The analysis assesses heterogeneity and horizontal pleiotropy for each sleep phenotype using two methods: IVW and MR Egger. The table reports heterogeneity statistics (Q, Q_df, P, I²) and the intercept, standard error (Se), and p-value for horizontal pleiotropy to assess the potential for confounding bias. FDR: False Discovery Rate, a statistical method for correcting for multiple testing to control the proportion of false positive findings

exposure	outcome	method	nsnp	or	se	or_lci95	or_uci95	pval	FDR
COPD	Chronotype	MR Egger	9	0.989051436	0.058408321	0.882065247	1.109014041	0.855846405	0.372084628
		Weighted median		1.039047746	0.021302428	0.996557844	1.083349276	0.07215544	
		Weighted mode		1.037992766	0.028045878	0.982474288	1.096648529	0.220322462	
		Simple mode		1.03745813	0.031304499	0.975716504	1.103106658	0.273890363	
		Inverse variance weighted (fixed effects)		1.018853006	0.015458055	0.988446958	1.050194389	0.226943754	
COPD	Daytime napping	MR Egger	14	1.02502301	0.010191186	1.00475159	1.045703418	0.032015457	0.06923657
		Weighted median		1.012462596	0.005202849	1.002190388	1.022840091	0.017287404	
		Weighted mode		1.010730809	0.005077616	1.000721776	1.020839951	0.055600526	
		Simple mode		1.010730809	0.009412226	0.992255821	1.029549786	0.277266039	
		Inverse variance weighted (fixed effects)		1.009613813	0.003695197	1.002328014	1.016952571	0.009617726	
COPD	Daytime Sleepiness	MR Egger	11	0.997534666	0.017789659	0.963352289	1.032929927	0.892700025	0.603101554
		Weighted median		0.987933987	0.006285783	0.975837177	1.000180753	0.053452546	
		Weighted mode		0.987295485	0.006444667	0.97490285	0.999845652	0.075375539	
		Simple mode		0.986284412	0.013140107	0.961207352	1.01201571	0.31798452	
		Inverse variance weighted (multiplicative random effects)		0.996800134	0.005968339	0.985207559	1.008529115	0.59126775	
COPD	Insomnia	MR Egger	10	1.021623589	0.021826102	0.978841017	1.066276076	0.35571375	0.272795442
		Weighted median		1.005745422	0.008405597	0.989311541	1.022452293	0.495512652	
		Weighted mode		1.003812846	0.009389465	0.985508275	1.022457402	0.694718751	
		Simple mode		1.018205179	0.016816882	0.985191107	1.052325564	0.31127489	
		Inverse variance weighted (fixed effects)		1.009689919	0.005889011	0.998102614	1.021411745	0.101525422	
COPD	Long sleep duration	MR Egger	11	0.994853465	0.008614494	0.978196966	1.011793587	0.563963251	0.719076179
		Weighted median		0.99746504	0.003972538	0.989728759	1.005261791	0.522867681	
		Weighted mode		0.99708236	0.004382503	0.988554394	1.005683895	0.520036503	
		Simple mode		0.996480996	0.005320369	0.986143761	1.006926591	0.522572164	
		Inverse variance weighted (fixed effects)		1.000015267	0.003045564	0.994063652	1.006002515	0.996000322	
COPD	Morning person	MR Egger	9	0.933386661	0.095093166	0.774669849	1.124621876	0.49201121	0.589304929
		Weighted median		1.032573364	0.036508213	0.961268031	1.109168012	0.379945408	
		Weighted mode		1.027541502	0.053961271	0.924414205	1.142173641	0.628186977	
		Simple mode		1.018110395	0.053009452	0.917639956	1.129581128	0.743635468	
		Inverse variance weighted (fixed effects)		1.015281907	0.025911216	0.965007247	1.068175762	0.55833355	
COPD	Short sleep duration	MR Egger	11	1.014279518	0.017370488	0.980328431	1.049406412	0.435432578	0.376226922
		Weighted median		1.001369611	0.005398634	0.990829658	1.012021683	0.799864605	
		Weighted mode		1.00145779	0.005998115	0.989753282	1.013300711	0.813019428	
		Simple mode		1.002540166	0.010043017	0.982998812	1.022469989	0.805687801	
		Inverse variance weighted (multiplicative random effects)		1.007024211	0.005890173	0.995465229	1.01871741	0.234690973	
COPD	Single-item chronotype	MR Egger	43	0.809005622	0.230818997	0.514605524	1.271828745	0.363858858	0.717262168
		Weighted median		0.906484863	0.114891756	0.72370691	1.135424846	0.392799367	
		Weighted mode		0.9016828	0.104529039	0.734643891	1.106702012	0.32780321	
		Simple mode		1.201265224	0.241084335	0.748899906	1.926877179	0.451128917	
		Inverse variance weighted (multiplicative random effects)		1.001492644	0.092346924	0.835680898	1.200203951	0.987113619	
COPD	Sleep duration	MR Egger	10	1.005566809	0.03922308	0.93115841	1.085921145	0.890947873	0.285759571
		Weighted median		0.991346311	0.013978826	0.964553626	1.018883223	0.534105557	
		Weighted mode		0.993315696	0.013616426	0.967156517	1.020182416	0.634124262	
		Simple mode		0.980940355	0.022893458	0.937897323	1.025958765	0.422356237	
		Inverse variance weighted (fixed effects)		0.984605161	0.00995402	0.965581808	1.004003301	0.119085501	
COPD	Snoring	MR Egger	12	0.999451113	0.014831143	0.970816239	1.028930591	0.971198236	0.516530763
		Weighted median		0.999838095	0.006071901	0.988009621	1.01180818	0.978725525	
		Weighted mode		0.999026716	0.00658619	0.986213229	1.012006683	0.885137643	
		Simple mode		1.00045098	0.009363606	0.982257496	1.018981446	0.962458062	
		Inverse variance weighted (fixed effects)		1.003769661	0.004623067	0.994715376	1.012906362	0.41571959	

**Table 7:** Colocalization analysis results This table presents the results of the colocalization analysis for various sleep phenotypes and COPD, reporting the posterior probabilities (PP) for different hypotheses, along with the number of SNPs (nsnps) and chromosomal positions. These results help assess the genetic overlap between sleep traits and COPD.

coloc_res										
p1	p2	p12	nsnps	PP.H0.abf	PP.H1.abf	PP.H2.abf	PP.H3.abf	PP.H4.abf		
1.00E-04	1.00E-04	1.00E-05	982	2.65E-07	2.50E-07	0.040359295	0.037084999	0.922555191		
Supplementary coloc-result-Insomnia										
p1	p2	p12	nsnps	PP.H0.abf	PP.H1.abf	PP.H2.abf	PP.H3.abf	PP.H4.abf	Chromosome	Position
1.00E-04	1.00E-04	1.00E-05	975	1.37E-05	5.50E-07	0.955224726	0.0384327	0.006328367	9	96345328
1.00E-04	1.00E-04	1.00E-05	606	0.000182549	7.40E-06	0.914846509	0.03704248	0.047921061	7	114076394
1.00E-04	1.00E-04	1.00E-05	720	0.005580291	0.001099854	0.78953341	0.155565847	0.048220598	13	54382035
1.00E-04	1.00E-04	1.00E-05	736	0.000466793	0.00660386	0.064226446	0.908609935	0.020092965	11	47441513
1.00E-04	1.00E-04	1.00E-05	1172	0.188324931	0.015670855	0.721558432	0.060027754	0.014418028	6	37631768
1.00E-04	1.00E-04	1.00E-05	645	0.001328992	4.44E-05	0.944400398	0.031562553	0.022663608	4	105330133
1.00E-04	1.00E-04	1.00E-05	842	0.000207101	1.72E-05	0.916416241	0.076225284	0.007134145	1	107185225
1.00E-04	1.00E-04	1.00E-05	726	2.81E-34	2.04E-35	0.920345723	0.066859745	0.012794531	2	66750564
1.00E-04	1.00E-04	1.00E-05	634	0.004445204	0.000171141	0.952634073	0.03667043	0.006079152	15	67804682
1.00E-04	1.00E-04	1.00E-05	634	0.001986681	7.21E-05	0.945649824	0.034280355	0.018011084	19	37684966
1.00E-04	1.00E-04	1.00E-05	1048	3.67E-05	1.58E-06	0.952243745	0.041084189	0.006633798	1	57850914
1.00E-04	1.00E-04	1.00E-05	606	0.021084547	0.000505221	0.944942596	0.022631578	0.010836058	5	87678585
1.00E-04	1.00E-04	1.00E-05	878	0.070958744	0.003817632	0.866622084	0.046612912	0.011988627	16	51484837
1.00E-04	1.00E-04	1.00E-05	878	0.000802008	3.55E-05	0.920325379	0.040707199	0.038129906	5	103889998
1.00E-04	1.00E-04	1.00E-05	1431	0.09047393	0.008335345	0.819639692	0.075507193	0.006043841	16	6106260
1.00E-04	1.00E-04	1.00E-05	720	0.299672858	0.014210888	0.648941992	0.030767291	0.00640697	17	28899614
1.00E-04	1.00E-04	1.00E-05	1035	0.037759816	0.002095681	0.901392991	0.050018845	0.008732667	4	91287477
1.00E-04	1.00E-04	1.00E-05	776	1.92E-06	3.15E-07	0.448885466	0.073064682	0.478047614	13	53786568
1.00E-04	1.00E-04	1.00E-05	769	2.21E-06	1.80E-07	0.80363722	0.065333518	0.131026872	18	52873317
1.00E-04	1.00E-04	1.00E-05	748	0.000199918	6.34E-06	0.963397403	0.030532655	0.005863687	16	59476179
1.00E-04	1.00E-04	1.00E-05	647	1.39E-05	4.50E-07	0.955113757	0.030876422	0.013995443	10	104241683
1.00E-04	1.00E-04	1.00E-05	719	0.00083686	2.87E-05	0.956511903	0.032846587	0.009775904	10	104680137
1.00E-04	1.00E-04	1.00E-05	688	4.06E-06	2.01E-07	0.91977376	0.045584349	0.034637633	1	201793440
1.00E-04	1.00E-04	1.00E-05	664	0.006263562	0.000210746	0.952517539	0.032039737	0.008968417	5	102543878
1.00E-04	1.00E-04	1.00E-05	918	0.009789366	0.000891151	0.839931784	0.076388129	0.07299957	12	84698234
1.00E-04	1.00E-04	1.00E-05	862	0.007960891	0.000404274	0.93454822	0.047449071	0.009637544	16	52633652
1.00E-04	1.00E-04	1.00E-05	530	0.000522385	1.33E-05	0.967740038	0.024706676	0.007017561	6	105400837
1.00E-04	1.00E-04	1.00E-05	569	0.001816197	4.90E-05	0.961831656	0.025932033	0.010371127	12	57487814
1.00E-04	1.00E-04	1.00E-05	708	9.00E-09	2.77E-10	0.964372205	0.029668737	0.005959049	2	58922921
1.00E-04	1.00E-04	1.00E-05	1499	0.004442421	0.000441775	0.898985224	0.089392554	0.006738025	7	1024581
1.00E-04	1.00E-04	1.00E-05	715	0.022022699	0.000611875	0.933943293	0.025931025	0.017491109	2	191288833
1.00E-04	1.00E-04	1.00E-05	487	7.24E-06	4.82E-07	0.923271569	0.061420749	0.015299959	3	49980596
1.00E-04	1.00E-04	1.00E-05	529	0.037861567	0.002097544	0.902564224	0.049994887	0.007481779	1	1686040
1.00E-04	1.00E-04	1.00E-05	1013	0.001461848	5.73E-05	0.951703839	0.037306645	0.00947035	13	59833519
1.00E-04	1.00E-04	1.00E-05	587	1.80E-05	4.99E-07	0.964445275	0.02670777	0.008828454	15	74340336
1.00E-04	1.00E-04	1.00E-05	811	1.28E-06	4.31E-08	0.960862989	0.032342411	0.006793278	2	114082175
1.00E-04	1.00E-04	1.00E-05	641	0.024669405	0.000857992	0.923739423	0.032108652	0.018624528	1	151738403
1.00E-04	1.00E-04	1.00E-05	988	0.001150983	0.000105704	0.874042915	0.080226014	0.044474384	12	109855201
1.00E-04	1.00E-04	1.00E-05	914	0.003700244	0.000121929	0.95894259	0.031593123	0.005642115	6	101246010
1.00E-04	1.00E-04	1.00E-05	717	1.90E-06	6.59E-08	0.939939572	0.032560563	0.027497898	5	135486536
1.00E-04	1.00E-04	1.00E-05	661	0.000951941	5.10E-05	0.938013866	0.050207579	0.010775651	10	104952499
1.00E-04	1.00E-04	1.00E-05	1010	0.050757173	0.002750112	0.77533847	0.041879914	0.129274331	3	116425935
1.00E-04	1.00E-04	1.00E-05	792	2.37E-10	1.34E-11	0.903132396	0.050847897	0.046019708	17	43223292
Supplementary -coloc-result-Short										
p1	p2	p12	nsnps	PP.H0.abf	PP.H1.abf	PP.H2.abf	PP.H3.abf	PP.H4.abf	Chromosome	Position
1.00E-04	1.00E-04	1.00E-05	1268	0.003594403	0.000377427	0.876657498	0.092025375	0.027345296	7	2080114
1.00E-04	1.00E-04	1.00E-05	609	3.19E-06	1.79E-07	0.910083202	0.050912681	0.039000746	7	114218582
1.00E-04	1.00E-04	1.00E-05	936	0.022822069	0.00107812	0.923665811	0.043625405	0.008808594	5	7249696
1.00E-04	1.00E-04	1.00E-05	751	0.004472393	0.034959639	0.107950167	0.843812052	0.008805749	1	98527951
1.00E-04	1.00E-04	1.00E-05	668	0.020588533	0.000640174	0.940439778	0.02923268	0.009098835	6	41792545
1.00E-04	1.00E-04	1.00E-05	608	6.69E-05	3.03E-06	0.804487895	0.03631407	0.159128111	18	53099093
1.00E-04	1.00E-04	1.00E-05	949	2.79E-06	1.91E-07	0.769398128	0.052504364	0.178094525	4	103188709
1.00E-04	1.00E-04	1.00E-05	822	2.42E-05	1.06E-06	0.940138015	0.041306286	0.018530448	2	57941287
1.00E-04	1.00E-04	1.00E-05	846	0.006532067	0.000243052	0.951065525	0.035381501	0.006777855	11	28808617
1.00E-04	1.00E-04	1.00E-05	761	0.001453432	0.000185648	0.800138148	0.102106232	0.09611654	4	82288564
1.00E-04	1.00E-04	1.00E-05	826	0.002640893	0.000141045	0.8948476	0.04773748	0.054632981	15	48027204
1.00E-04	1.00E-04	1.00E-05	475	0.247632808	0.020421814	0.658723725	0.054304797	0.018916855	3	50172397
1.00E-04	1.00E-04	1.00E-05	1093	0.097534399	0.005804067	0.809335272	0.048122636	0.039203626	17	11227352
1.00E-04	1.00E-04	1.00E-05	780	0.015433322	0.000812741	0.771304614	0.040446027	0.172003296	1	66470206
1.00E-04	1.00E-04	1.00E-05	3219	0.005917568	0.003676355	0.606017372	0.376487065	0.00790164	6	29832846
1.00E-04	1.00E-04	1.00E-05	760	0.001369526	7.05E-05	0.860957631	0.044251713	0.09335059	1	201870221
1.00E-04	1.00E-04	1.00E-05	787	7.85E-12	2.57E-13	0.961801681	0.031483152	0.006715167	2	114089551
1.00E-04	1.00E-04	1.00E-05	670	0.010463525	0.000305279	0.955349759	0.027866841	0.006014596	5	102327868
1.00E-04	1.00E-04	1.00E-05	662	0.000184598	6.32E-06	0.940226797	0.032162778	0.027419507	5	135508381
1.00E-04	1.00E-04	1.00E-05	724	0.00296822	0.000133177	0.939725072	0.042148136	0.015025395	22	39838892
1.00E-04	1.00E-04	1.00E-05	860	0.012240394	0.000436819	0.94627561	0.033762147	0.00728503	16	56227965
1.00E-04	1.00E-04	1.00E-05	971	0.010509648	0.000369237	0.936054409	0.032866346	0.020200361	8	52886619
1.00E-04	1.00E-04	1.00E-05	1044	0.04437758	0.002840622	0.889666887	0.056941677	0.006173234	1	34736052
1.00E-04	1.00E-04	1.00E-05	735	0.000505694	1.77E-05	0.953984217	0.033297373	0.012195059	2	58871658
1.00E-04	1.00E-04	1.00E-05	580	0.060301956	0.239204272	0.137687136	0.546157192	0.016649444	11	47980568
1.00E-04	1.00E-04	1.00E-05	846	0.241149372	0.008729506	0.719357576	0.026035713	0.004727833	6	129848635
1.00E-04	1.00E-04	1.00E-05	1020	0.067494965	0.003439217	0.873829348	0.044515396	0.010721074	6	55040290
Supplementary -coloc-result-Daytime napping										
p1	p2	p12	nsnps	PP.H0.abf	PP.H1.abf	PP.H2.abf	PP.H3.abf	PP.H4.abf	Chromosome	Position
1.00E-04	1.00E-04	1.00E-05	706	0.000424077	2.04E-05	0.939956032	0.045268437	0.014331024	3	183995341
1.00E-04	1.00E-04	1.00E-05	599	3.58E-06	2.35E-07	0.926402509	0.060768828	0.012824849	14	29721044
1.00E-04	1.00E-04	1.00E-05	979	0.026205042	0.001052743	0.927433657	0.037250018	0.00805854	15	93510243
1.00E-04	1.00E-04	1.00E-05	450	0.000543562	1.72E-05	0.841386085	0.026560074	0.131493035	7	99188589
1.00E-04	1.00E-04	1.00E-05	842	0.005444515	0.000161775	0.959265429	0.02849638	0.0066319	9	20962282
1.00E-04	1.00E-04	1.00E-05	847	0.000143091	4.71E-06	0.954950836	0.031426821	0.013474541	11	28866669
1.00E-04	1.00E-04	1.00E-05	1203	0.013516896	0.000588566	0.935179051	0.040710494	0.010004993	11	8335754
1.00E-04	1.00E-04	1.00E-05	602	0.016374239	0.000688584	0.932463447	0.039201495	0.011272236	5	143769614
1.00E-04	1.00E-04	1.00E-05	1083	1.26E-10	5.49E-12	0.952808455	0.041437061	0.005754484	5	146693062
1.00E-04	1.00E-04	1.00E-05	850	0.020799975	0.001230181	0.90912078	0.053753384	0.015095681	15	63793936
1.00E-04	1.00E-04	1.00E-05	829	6.34E-05	1.12E-05	0.829939617	0.146699998	0.023285744	2	59478517
1.00E-04	1.00E-04	1.00E-05	1267	0.000692559	4.70E-05	0.882784326	0.059857188	0.056618925	11	101479583
1.00E-04	1.00E-04	1.00E-05	988	0.015171885	0.000632636	0.938977234	0.03914731	0.006070936	17	64308310
1.00E-04	1.00E-04	1.00E-05	1182	5.40E-07	2.73E-08	0.945326464	0.047863202	0.006809766	10	13868855
1.00E-04	1.00E-04	1.00E-05	812	1.52E-42	5.75E-44	0.954596874	0.036125057	0.009278069	12	117942076
1.00E-04	1.00E-04	1.00E-05	835	9.78E-07	3.38E-08	0.957713417	0.033050051	0.00923552	16	56193408
1.00E-04	1.00E-04	1.00E-05	761	0.000686762	2.39E-05	0.958025447	0.033321095	0.007942804	6	28199764
1.00E-04	1.00E-04	1.00E-05	1096	0.004724099	0.000209111	0.943504986	0.041754261	0.009807543	1	162876200
1.00E-04	1.00E-04	1.00E-05	1144	2.05E-24	1.37E-25	0.924517649	0.061936016	0.013546336	1	62579891
1.00E-04	1.00E-04	1.00E-05	696	0.004230635	0.000146465	0.951824486	0.032941324	0.01085709	9	131840856
1.00E-04	1.00E-04	1.00E-05	539	5.43E-07	1.40E-08	0.967823918	0.024965967	0.007209557	17	35595368
1.00E-04	1.00E-04	1.00E-05	1063	0.00029285	2.11E-05	0.82465209	0.059256273	0.115777703	5	62760828
1.00E-04	1.00E-04	1.00E-05	1023	0.000469009	1.59E-05	0.959544361	0.032608225	0.007362462	2	49413860
1.00E-04	1.00E-04	1.00E-05	982	2.65E-07	2.50E-07	0.040359295	0.037084999	0.922555191	2	23611438
1.00E-04	1.00E-04	1.00E-05	665	0.000233289	7.60E-06	0.958087297	0.031221356	0.010450453	4	85408000
1.00E-04	1.00E-04	1.00E-05	2453	0.020255389	0.0019584	0.881962538	0.085262305	0.010561369	8	1175131
1.00E-04	1.00E-04	1.00E-05	901	6.16E-17	3.27E-18	0.942332732	0.050024153	0.007643115	9	81727018
1.00E-04	1.00E-04	1.00E-05	681	0.002006575	0.000116103	0.869630637	0.050239921	0.078006764	16	69402831
1.00E-04	1.00E-04	1.00E-05	692	0.049010739	0.001715135	0.892394455	0.031203742	0.025675928	6	155129280
1.00E-04	1.00E-04	1.00E-05	1109	0.010504837	0.000369693	0.946231791	0.033290828	0.009602852	9	73504992
1.00E-04	1.00E-04	1.00E-05	635	4.28E-06	1.46E-07	0.960941459	0.032785664	0.006268453	6	84314423
1.00E-04	1.00E-04	1.00E-05	906	3.95E-06	2.57E-06	0.587079559	0.381862016	0.031051908	3	84665393
1.00E-04	1.00E-04	1.00E-05	771	0.00290489	0.000102014	0.943900077	0.033127919	0.0199651	2	58905715
1.00E-04	1.00E-04	1.00E-05	543	0.000342243	8.05E-06	0.968816247	0.02278545	0.008048008	15	83235408
1.00E-04	1.00E-04	1.00E-05	643	0.005157125	0.00017563	0.941985222	0.032059445	0.020622578	20	39832628
1.00E-04	1.00E-04	1.00E-05	617	2.54E-08	1.42E-09	0.913823292	0.050809375	0.035367306	11	61565908
1.00E-04	1.00E-04	1.00E-05	999	0.022972376	0.001163463	0.858099433	0.043385088	0.074379639	9	37441650
1.00E-04	1.00E-04	1.00E-05	791	0.006436745	0.013177981	0.317114708	0.64921652	0.014054045	1	98589715
1.00E-04	1.00E-04	1.00E-05	1287	0.00072847	3.80E-05	0.943933463	0.049229383	0.006070687	21	47397586
1.00E-04	1.00E-04	1.00E-05	672	2.43E-09	7.59E-11	0.963922304	0.030146193	0.005931501	5	102293380
1.00E-04	1.00E-04	1.00E-05	982	0.000301016	1.45E-05	0.94714905	0.04554437	0.006991087	1	96928273
1.00E-04	1.00E-04	1.00E-05	552	3.66E-06	1.97E-07	0.935424424	0.050424585	0.014147136	1	174316633
1.00E-04	1.00E-04	1.00E-05	811	0.024130932	0.0009175	0.931395106	0.035405129	0.008151333	8	67381858
1.00E-04	1.00E-04	1.00E-05	1195	0.000321587	1.69E-05	0.919801776	0.048210342	0.031649429	5	112030173
1.00E-04	1.00E-04	1.00E-05	969	0.000232471	1.06E-05	0.92373318	0.041987072	0.034036702	10	64552010
1.00E-04	1.00E-04	1.00E-05	768	2.19E-18	1.14E-19	0.843395723	0.043683403	0.112920875	1	201860626
1.00E-04	1.00E-04	1.00E-05	692	0.042312017	0.001258923	0.900328287	0.026758412	0.029342361	22	37096573
1.00E-04	1.00E-04	1.00E-05	1033	0.002144242	7.32E-05	0.958453636	0.032716632	0.006612282	12	13493906
1.00E-04	1.00E-04	1.00E-05	964	5.40E-17	3.92E-18	0.924140532	0.067066304	0.008793164	5	103947968
1.00E-04	1.00E-04	1.00E-05	1219	1.08E-22	6.04E-24	0.936819462	0.052249772	0.010930766	6	55142337
1.00E-04	1.00E-04	1.00E-05	884	0.015728529	0.000617581	0.934240409	0.03667024	0.012743242	3	133032892
1.00E-04	1.00E-04	1.00E-05	933	0.000413764	1.82E-05	0.944796168	0.041434673	0.013337243	11	92769748
1.00E-04	1.00E-04	1.00E-05	909	7.44E-05	4.54E-06	0.936964521	0.057187079	0.005769482	13	107820389
1.00E-04	1.00E-04	1.00E-05	637	3.09E-06	4.68E-07	0.442706973	0.066477075	0.49081239	1	33342304
1.00E-04	1.00E-04	1.00E-05	900	0.033128028	0.001903055	0.859340213	0.049308868	0.056319836	21	40506194
1.00E-04	1.00E-04	1.00E-05	804	0.003333752	0.000106114	0.959189395	0.030524289	0.00684645	8	106108303
1.00E-04	1.00E-04	1.00E-05	889	5.11E-08	2.45E-09	0.947810628	0.045446465	0.006742854	18	36105007
1.00E-04	1.00E-04	1.00E-05	908	0.103496015	0.003696511	0.834059764	0.029760677	0.028987032	1	95783037
1.00E-04	1.00E-04	1.00E-05	661	0.008018023	0.000339175	0.944759318	0.039957848	0.006925637	5	89597733
1.00E-04	1.00E-04	1.00E-05	776	0.002114989	9.88E-05	0.941749432	0.043979429	0.012057354	6	36224315
1.00E-04	1.00E-04	1.00E-05	655	0.108732618	0.012256845	0.784542749	0.088431263	0.006036525	18	31610848
1.00E-04	1.00E-04	1.00E-05	760	0.000522487	2.84E-05	0.935486689	0.050834596	0.013127829	12	38616581
1.00E-04	1.00E-04	1.00E-05	811	0.000187326	9.91E-06	0.940538776	0.049724513	0.00953948	1	171516863
1.00E-04	1.00E-04	1.00E-05	1368	0.000103564	4.87E-06	0.943050051	0.04429698	0.012544539	2	52934600
1.00E-04	1.00E-04	1.00E-05	1034	0.024110033	0.000944348	0.926905198	0.03629351	0.011746912	2	169092428
1.00E-04	1.00E-04	1.00E-05	750	0.004290048	0.000137284	0.956673143	0.030605759	0.008293766	8	28191306
1.00E-04	1.00E-04	1.00E-05	905	0.176338649	0.008706742	0.763567285	0.037687526	0.013699798	7	31330785
1.00E-04	1.00E-04	1.00E-05	868	0.01225047	0.000424735	0.937230512	0.032476999	0.017617284	2	206831860
1.00E-04	1.00E-04	1.00E-05	1238	0.022037756	0.011599448	0.544387042	0.286399462	0.135576291	20	62194103
1.00E-04	1.00E-04	1.00E-05	688	2.34E-41	2.53E-42	0.786900621	0.084948583	0.128150796	17	43686419
1.00E-04	1.00E-04	1.00E-05	820	0.025122884	0.000818911	0.928178407	0.030239458	0.01564034	5	106861892
1.00E-04	1.00E-04	1.00E-05	1362	0.000563278	4.82E-05	0.876091808	0.07493894	0.048357762	17	79084367
1.00E-04	1.00E-04	1.00E-05	318	0.016263555	0.000256607	0.947544996	0.014929411	0.021005431	16	28350059
1.00E-04	1.00E-04	1.00E-05	1052	2.07E-24	1.12E-25	0.935550053	0.050741961	0.013707987	1	62483623
1.00E-04	1.00E-04	1.00E-05	802	0.001276652	4.35E-05	0.956935472	0.032624555	0.009119784	6	38470087
1.00E-04	1.00E-04	1.00E-05	821	0.078458015	0.002586306	0.808920966	0.026581982	0.083452732	4	164216739
1.00E-04	1.00E-04	1.00E-05	1032	0.032271163	0.00176501	0.903929023	0.04942607	0.012608735	5	106311039
1.00E-04	1.00E-04	1.00E-05	1189	0.065359867	0.003425599	0.878654659	0.046044958	0.006514917	9	120555845
1.00E-04	1.00E-04	1.00E-05	877	5.64E-05	2.74E-06	0.808555109	0.039154668	0.152231096	17	43127715
1.00E-04	1.00E-04	1.00E-05	661	0.004066708	0.000147011	0.941582744	0.034018008	0.020185529	14	29218473
1.00E-04	1.00E-04	1.00E-05	1152	1.70E-41	2.40E-42	0.775395397	0.109702389	0.114902214	17	43798360
1.00E-04	1.00E-04	1.00E-05	688	0.027701648	0.000909146	0.925310585	0.030352237	0.015726384	2	25337155
1.00E-04	1.00E-04	1.00E-05	950	0.000115719	8.19E-06	0.92527077	0.065464668	0.009140655	12	108334477
1.00E-04	1.00E-04	1.00E-05	1500	0.002650629	0.00015193	0.937580009	0.053734666	0.005882766	16	10134637
1.00E-04	1.00E-04	1.00E-05	763	9.59E-12	1.84E-12	0.833521163	0.160247882	0.006230955	6	124920871
1.00E-04	1.00E-04	1.00E-05	711	0.001594301	6.25E-05	0.951468734	0.037267443	0.009607059	2	162573586
1.00E-04	1.00E-04	1.00E-05	875	0.073163377	0.00280418	0.881227457	0.033766335	0.009038651	9	34081331
1.00E-04	1.00E-04	1.00E-05	654	0.000369586	9.73E-06	0.962611207	0.025323247	0.011686233	5	87712831
1.00E-04	1.00E-04	1.00E-05	1080	9.45E-10	6.30E-11	0.931564726	0.06211196	0.006323313	6	37699156
1.00E-04	1.00E-04	1.00E-05	1201	0.005795353	0.000223243	0.941768756	0.036262025	0.015950623	5	159042652
1.00E-04	1.00E-04	1.00E-05	1016	0.006031493	0.000187703	0.957348346	0.02978647	0.006645989	2	208941004
1.00E-04	1.00E-04	1.00E-05	680	0.045302991	0.001473066	0.48007217	0.015151962	0.45799981	3	62459819
1.00E-04	1.00E-04	1.00E-05	624	0.010188977	0.000283342	0.954429509	0.026532831	0.008565342	3	19377311
1.00E-04	1.00E-04	1.00E-05	1102	0.000314416	1.61E-05	0.886306844	0.045351614	0.068011014	6	54773639
1.00E-04	1.00E-04	1.00E-05	909	0.070811273	0.00592092	0.749865287	0.062589548	0.110812972	8	142213997
1.00E-04	1.00E-04	1.00E-05	660	0.025020188	0.000757436	0.938639828	0.028408275	0.007174273	6	114650655
1.00E-04	1.00E-04	1.00E-05	559	0.000176623	4.53E-06	0.965019207	0.024766296	0.01003334	2	164574344
1.00E-04	1.00E-04	1.00E-05	657	0.091321909	0.002985772	0.870032717	0.028438516	0.007221086	18	25562831
1.00E-04	1.00E-04	1.00E-05	874	1.12E-10	6.83E-12	0.888360148	0.054124201	0.057515651	2	236800163
1.00E-04	1.00E-04	1.00E-05	684	0.022027105	0.000595345	0.94447031	0.025519609	0.00738763	20	45841052
1.00E-04	1.00E-04	1.00E-05	807	0.118926552	0.008417517	0.809768204	0.057309107	0.00557862	6	51829890
1.00E-04	1.00E-04	1.00E-05	776	0.003916975	0.000174898	0.922104404	0.04114038	0.032663344	2	151500518
1.00E-04	1.00E-04	1.00E-05	869	0.01843796	0.00098812	0.924212396	0.049523214	0.006838309	2	44557919
1.00E-04	1.00E-04	1.00E-05	612	0.007275586	0.000189952	0.95752298	0.024989127	0.010022355	3	44385814
1.00E-04	1.00E-04	1.00E-05	1072	0.00179768	8.88E-05	0.903866986	0.044607289	0.049639228	6	100079774
1.00E-04	1.00E-04	1.00E-05	1043	0.0118168	0.006986635	0.613696263	0.362840735	0.004659567	6	170621249
1.00E-04	1.00E-04	1.00E-05	857	0.000178787	6.41E-06	0.958184864	0.034361383	0.007268552	18	23084118
1.00E-04	1.00E-04	1.00E-05	818	0.004491866	0.000369812	0.911921533	0.075069665	0.008147124	9	108846445
1.00E-04	1.00E-04	1.00E-05	857	0.000429387	1.97E-05	0.941504433	0.043235092	0.014811364	3	82774831
1.00E-04	1.00E-04	1.00E-05	1063	0.003874404	0.000268629	0.846443204	0.058596645	0.090817118	16	23837048
1.00E-04	1.00E-04	1.00E-05	935	5.12E-23	1.79E-23	0.228589007	0.078977429	0.692433564	18	44788274
1.00E-04	1.00E-04	1.00E-05	776	0.000153095	4.67E-06	0.963165529	0.029356029	0.00732068	4	79550425

## References

[1] Orozco RJ, Rodriguez D, Hunter K, Roy S (2024). The 2021 Global Initiative for Chronic Obstructive Pulmonary Disease (GOLD) guidelines and the outpatient management: examining physician adherence and its effects on patient outcome. J Family Med Prim Care.

[2] Budhiraja R, Siddiqi TA, Quan SF (2015). Sleep disorders in chronic obstructive pulmonary disease: etiology, impact, and management. J Clin Sleep Med.

[3] McNicholas WT, Verbraecken J, Marin JM (2013). Sleep disorders in COPD: the forgotten dimension. Eur Respir Rev.

[4] Vaidya S, Gothi D, Patro M (2020). Prevalence of sleep disorders in chronic obstructive pulmonary disease and utility of global sleep assessment questionnaire: an observational case-control study. Ann Thorac Med.

[5] Holmes MV, Ala-Korpela M, Smith GD (2017). Mendelian randomization in cardiometabolic disease: challenges in evaluating causality. Nat Rev Cardiol.

[6] Lu T, Forgetta V, Greenwood CM, Zhou S, Richards JB (2023). Circulating proteins influencing psychiatric disease: a Mendelian randomization study. Biol Psychiatry.

[7] Davey Smith G, Holmes MV, Davies NM, Ebrahim S (2020). Mendel's laws, Mendelian randomization and causal inference in observational data: substantive and nomenclatural issues. Eur J Epidemiol.

[8] Riemann D, Krone LB, Wulff K, Nissen C (2020). Sleep, insomnia, and depression. Neuropsychopharmacology.

[9] Jansen PR, Watanabe K, Stringer S (2019). Genome-wide analysis of insomnia in 1,331,010 individuals identifies new risk loci and functional pathways. Nat Genet.

[10] Hjetland GJ, Pallesen S, Thun E, Kolberg E, Nordhus IH, Flo E (2020). Light interventions and sleep, circadian, behavioral, and psychological disturbances in dementia: a systematic review of methods and outcomes. Sleep Med Rev.

[11] Dashti HS, Daghlas I, Lane JM (2021). Genetic determinants of daytime napping and effects on cardiometabolic health. Nat Commun.

[12] Bowden J, Del Greco MF, Minelli C, Davey Smith G, Sheehan N, Thompson J (2017). A framework for the investigation of pleiotropy in two-sample summary data Mendelian randomization. Stat Med.

[13] Sudlow C, Gallacher J, Allen N (2015). UK Biobank: an open access resource for identifying the causes of a wide range of complex diseases of middle and old age. PLoS Med.

[14] Wang H, Lane JM, Jones SE (2019). Genome-wide association analysis of self-reported daytime sleepiness identifies 42 loci that suggest biological subtypes. Nat Commun.

[15] Lane JM, Jones SE, Dashti HS (2019). Biological and clinical insights from genetics of insomnia symptoms. Nat Genet.

[16] Dashti HS, Jones SE, Wood AR (2019). Genome-wide association study identifies genetic loci for self-reported habitual sleep duration supported by accelerometer-derived estimates. Nat Commun.

[17] Arnold M, Raffler J, Pfeufer A, Suhre K, Kastenmüller G (2015). SNiPA: an interactive, genetic variant-centered annotation browser. Bioinformatics.

[18] Zhang H (2023). Pros and cons of Mendelian randomization. Fertil Steril.

[19] Boehm FJ, Zhou X (2022). Statistical methods for Mendelian randomization in genome-wide association studies: a review. Comput Struct Biotechnol J.

[20] Giambartolomei C, Vukcevic D, Schadt EE, Franke L, Hingorani AD, Wallace C, Plagnol V (2014). Bayesian test for colocalisation between pairs of genetic association studies using summary statistics. PLoS Genet.

[21] Wijesooriya K, Jadaan SA, Perera KL, Kaur T, Ziemann M (2022). Urgent need for consistent standards in functional enrichment analysis. PLoS Comput Biol.

[22] Li SQ, Sun XW, Zhang L (2021). Impact of insomnia and obstructive sleep apnea on the risk of acute exacerbation of chronic obstructive pulmonary disease. Sleep Med Rev.

[23] Leng Y, Wainwright NW, Cappuccio FP (2016). Daytime napping and increased risk of incident respiratory diseases: symptom, marker, or risk factor?. Sleep Med.

[24] Ruan Z, Li D, Cheng X, Jin M, Liu Y, Qiu Z, Chen X (2023). The association between sleep duration, respiratory symptoms, asthma, and COPD in adults. Front Med (Lausanne).

[25] Hong PY, Liu D, Liu A, Su X, Zhang XB, Zeng YM (2024). Causal associations of obstructive sleep apnea with chronic respiratory diseases: a Mendelian randomization study. BMC Pulm Med.

[26] Mohamed NA, Ahmed AL, Elayari OS (2022). Quality of sleeping among patients with chronic obstructive pulmonary disease. Int Egypt J Nurs Sci Res.

[27] Clímaco DC, Lustosa TC, Silva MV (2022). Sleep quality in COPD patients: correlation with disease severity and health status. J Bras Pneumol.

[28] Pande B, Sinha M, Sinha R, Behera AK, Parganiha A, Nanda R, Singh LK (2024). Cognitive correlates of circadian rhythm and sleep-wake behaviour in chronic obstructive pulmonary disease patients. Chronobiol Int.

[29] Hynninen MJ, Pallesen S, Hardie J, Eagan TM, Bjorvatn B, Bakke P, Nordhus IH (2013). Insomnia symptoms, objectively measured sleep, and disease severity in chronic obstructive pulmonary disease outpatients. Sleep Med.

[30] Kapella M, Steffen A, Prasad B (2022). Therapy for insomnia with chronic obstructive pulmonary disease: a randomized trial of components. J Clin Sleep Med.

